# Stereochemical engineering yields a multifunctional peptide macrocycle inhibitor of Akt2 by fine-tuning macrocycle-cell membrane interactions

**DOI:** 10.1038/s42004-023-00890-w

**Published:** 2023-05-18

**Authors:** Arundhati Nag, Amirhossein Mafi, Samir Das, Mary Beth Yu, Belen Alvarez-Villalonga, Soo-Kyung Kim, Yapeng Su, William A. Goddard, James R. Heath

**Affiliations:** 1 California Institute of Technology, Pasadena, CA USA; 2 Present Address: Materials and Process Simulation Center (139-74), California Institute of Technology, Pasadena, CA USA; 3 Present Address: Clark University, Worcester, MA USA; 4 Present Address: Institute for Systems Biology, Seattle, WA USA

**Keywords:** Peptides, Screening, Kinases, Chemical tools, Computational chemistry

## Abstract

Macrocycle peptides are promising constructs for imaging and inhibiting extracellular, and cell membrane proteins, but their use for targeting intracellular proteins is typically limited by poor cell penetration. We report the development of a cell-penetrant high-affinity peptide ligand targeted to the phosphorylated Ser474 epitope of the (active) Akt2 kinase. This peptide can function as an allosteric inhibitor, an immunoprecipitation reagent, and a live cell immunohistochemical staining reagent. Two cell penetrant stereoisomers were prepared and shown to exhibit similar target binding affinities and hydrophobic character but 2-3-fold different rates of cell penetration. Experimental and computational studies resolved that the ligands’ difference in cell penetration could be assigned to their differential interactions with cholesterol in the membrane. These results expand the tool kit for designing new chiral-based cell-penetrant ligands.

## Abstract (web summary)

Macrocycle peptides are promising constructs for imaging and inhibiting extracellular and cell membrane proteins, but their use for targeting intracellular proteins is typically limited by poor cell penetration. Here, the authors report the development of a cell-penetrant high-affinity peptide ligand targeted to the phosphorylated Ser474 epitope of the (active) Akt2 kinase.

## Introduction

Phospho-kinase signaling is the primary method of regulating trans-membrane and intracellular signal transduction pathways^1^. Genetically encoded hyper-activation of such pathways within many cancer cell types is reflected in elevated phosphorylation levels of the individual protein kinase pathway elements relative to that seen in healthy cells^2^. Thus, phospho-kinases can serve as both important drug and diagnostic targets. Small molecules are the standard tools for kinase inhibition, while phospho-specific antibodies are diagnostic tools to, for example, stain fixed tumor tissues to help define tumor margins^3^. Similar imaging in live tissues would be valuable but is impractical because phospho-specific antibodies are not cell-penetrant^4^. One molecule class that might simultaneously enable both small molecule and antibody-like applications is that of epitope-specific^5^ macrocyclic peptides^6^. While some naturally occurring peptide macrocycles are cell-penetrant^7^, most are not. Synthetic toolkits for optimizing cyclic peptides for cell penetration^8^ have been reported^9^; however, for targeted macrocycles, such modifications need to be balanced with the need to retain target-specific characteristics^10^ such as binding and inhibition. This balancing act presents a formidable challenge. Consequently, cell-penetrating peptide sequences (CPPs) are often appended to targeted moieties to promote cell penetration^11^, but CPPs can limit therapeutic applications^12^. Here we explore multiple synthetic and computational approaches to develop a cell-penetrant, peptide macrocycle inhibitor targeted to the phosphorylated (active) Protein Kinase B (PKB/Akt2). Akt2 can function as an oncoprotein^13^ and is hyperactivated via phosphorylation in multiple cancers^14,15^, so it is an important therapeutic and diagnostic target.

We used the overall objective of developing a cell-penetrating Akt2 inhibitor to explore a novel chemical algorithm in which the tasks of binding, inhibition, and cell penetration are each addressed separately and sequentially. This algorithm drew inspiration from both monoclonal antibody (mAb) development and small molecule drug development while circumventing certain drawbacks of both. Similar to mAb development, our approach does not require the presence of a hydrophobic binding pocket at the targeted protein region. Removing this limitation freed us from many challenges associated with identifying small molecule inhibitors. We previously showed that the unstructured C-terminal domain of pAkt2 containing a kinase-activating phosphorylation site at Ser474 but no hydrophobic binding pocket could provide a target for peptide binding and allosteric inhibition of Akt2^16^. Once a cyclic ligand against this epitope was identified^5^, the chemical flexibility and relatively small size of the ligand allowed us to utilize the molecular tools of medicinal chemistry to optimize the ligand’s kinase inhibition and cell penetration properties. An intriguing result is that the stereochemical fine-tuning of the peptide macrocycle provided a powerful toolset for promoting cell penetration while allowing the macrocycle to retain both its pAkt2 binding affinity and its pAkt2 inhibition characteristics. We further explored the mechanism of cell penetration through experiments and large-scale molecular dynamics (MD) calculations to provide atomistic insight into this stereochemistry toolset. The optimized cell-penetrant macrocycle exhibits stability against proteases. It works well as a cell-penetrant inhibitor of Akt2, a reagent for imaging Akt2 activation in live cells, and a pAkt2 immunoprecipitation reagent.

## Results

**Fig. 1:**
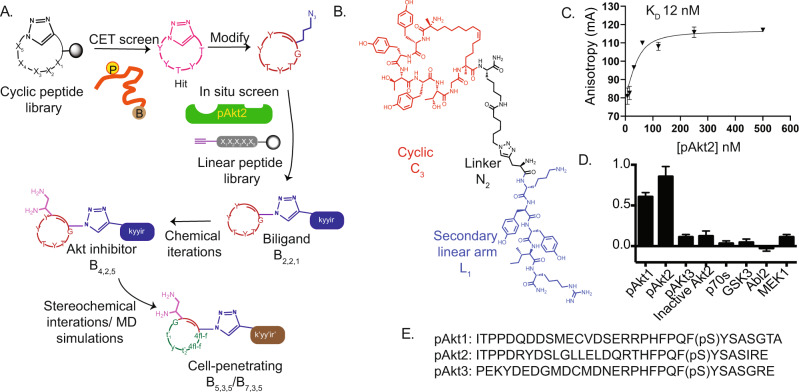
The workflow to produce a cell-penetrant biligand inhibitor of pAkt2 and the structure and affinity characterizations of the first generation (non-cell-penetrant) inhibitor B_3,2,1_. **A**. Following the chemical epitope targeting (CET) screen, the cyclic ligand **C**_**1**_ is modified for use in the in situ click biligand screen - which results in the isolation of the biligand **B**_**2,2,1**_. **B**. Appending diamino propanoic acid (Dap) at the N-terminal of **B**_**2,2,1**_ yielded **B**_**3,2,1**_, the first-generation pAkt2 inhibitor. **C**. Fluorescence anisotropy data (n = 3) yielded a 12 nM dissociation constant (K_D_) of **B**_**3,2,1**_ for pAkt2. Error bars indicate standard deviation. **D**. Selectivity ELISA assays with immobilized biotinylated **B**_**3,2,1**_, titrated with His_6_-tagged kinases, show that **B**_**3,2,1**_ selectively bound to pAkt2 and pAkt1 relative to p and non-phosphorylated Akt2. Values represent the mean A_450_ (n = 3), with standard deviation errors. **E**. Sequences of the targeted epitope of pAkt2 and the analogous C-terminal regions of isoform pAkt1 and pAkt3 proteins.

The development of the cell penetrant anti-pSer474 Akt2 biligand proceeded through four phases (Fig. 1A), starting with Chemical Epitope Targeting (CET). CET has been demonstrated for isolating peptide binders to specific epitopes of intracellular, membrane, or extracellular proteins^16–21^. CET starts with a chemically synthesized biotin-tagged protein fragment corresponding to a protein’s targeted region (epitope) (the SynEp). Next, a One-Bead-One-Compound (OBOC)^22^ cyclic library of peptides is screened in a multi-step process against possible interferents and then against the SynEp to identify candidate binders. Finally, hit compounds are validated against the full-length phosphoprotein to select a ‘best’ macrocycle.

In phase 2, that macrocycle is extended using an in situ click screen^23^ to yield a peptide macrocycle (**C**_**i**_)-linker (**Nj**)-peptide (**L**_**k**_) biligand (**B**_**i,j,k**_). The azide-modified macrocycle is screened against a comprehensive library of hexameric alkynyl-presenting peptides for the in situ click screen, in the presence of pAkt2. The basic idea is that the pAkt2 protein provides a scaffold for bringing together the two peptides in just the precise orientation to promote the cycloaddition reaction between the azide and alkyne to form a triazole linkage. The concept of the in situ click screen was first reported by Sharpless’ group^24^, and generalized by us over the past several years^25^. The primary motivation behind extending the macrocycle to a biligand is to increase the affinity of **B**_**i,j,k**_ for pAkt2. For the i, j, and k numerical indices, higher numbers represent later-generation biligands. In phase 3, **B**_**i,j,k**_ is optimized for pAkt2 inhibition while retaining affinity characteristics. In phase 4, **B**_**i,j,k**_ is optimized for cell penetration while retaining inhibition and affinity characteristics.

### Development of Ci

A nearly two million element OBOC macrocyclic peptide library was screened against a 32 amino acid long phosphopeptide representing the unstructured C-terminal region of pAkt2 (see Methods). This SynEp contained a hydrophobic motif (HF), the phosphorylated Ser474 residue, and an N-terminal biotin attached through a polyethylene glycol linker. Phosphorylation of Ser474 in the protein by mTORC2 results in an allosteric, activating influence over the kinase^26^. A non-phosphorylated form of the SynEp was also prepared, in which nine residues, including Ser474, were scrambled. The OBOC macrocyclic library was screened (Supplementary Scheme S1, Supplementary Table S1) against the non-phosphorylated^5^ scrambled SynEp, to remove false positives (roughly 10% of the library). The remaining OBOC library was then screened against the phosphorylated SynEp to yield four hits (Supplementary Table S2). These hits were sequenced using Edman degradation, synthesized, and tested for differential binding to the phosphorylated epitope and the scrambled epitope, and for binding against full length pAkt2 (see Methods, SI Figure S.1)^5^. The best macrocycle **C**_**1**_ (Supplementary Figure S1) exhibited an EC_50_ of 120 nM against pAkt2^5^.

Peptide macrocycles often exhibit improved binding relative to their linear counterparts – an attribute often ascribed to reduced conformational entropy^27^.We verified that the cyclization of **C**_**1**_ did indeed have a significant positive effect on binding (Supplementary Figure S2). We then sought to improve macrocycle-pAkt2 binding affinity by testing several macrocycle analogs varying in ring size (Supplementary Figure. S3, Supplementary Figure. S4) and the nature of the cyclization linker (Supplementary Figure. S4) for binding to the SynEp and full-length pAkt2. The best ligand **C**_**2**_ had the sequence Cy(YYTYTG-rcm) (Supplementary Figure S5). Here, Cy implies the cyclic nature of the ligand and rcm refers to alkenyl ring closing functionality synthesized by Ruthenium catalyzed Ring Closing Metathesis (RCM) reaction between unnatural amino acids (R)-α-(7-Octenyl)alanine (R_8_) and (S)−2-(4-pentenyl)alanine) (S_5_)^28^. **C**_**2**_ exhibited improved affinity (EC_50_ 44 nM) (Supplementary Figure S5) and selectivity relative to **C**_**1**_ (Supplementary Figure S6) against pAkt2.

### Extension of Ci into the B_i,j,k_ biligand

We sought to improve the binding performance of **C**_**2**_ to pAkt2 by using in situ click chemistry^23,25^ to extend **C**_**2**_ into a biligand. For this purpose, we appended an azide click handle and a biotin tag on **C**_**2**_ to form **C**_**2**_**-N**_**1**_ (Supplementary Figure S7). For the in situ screen, an OBOC linear peptide library was constructed from D-amino acids, to promote stability against proteases, and contained an alkyne on the N terminal. Thus, the goal of the in situ click screen was for **C**_**2**_**-N**_**1**_ to selectively react with specific library elements, catalyzed by pAkt2 protein, to form a biligand **B**_**i,j,k**_.

The OBOC library was first screened against **C**_**2**_**-N**_**1**_ to remove hits that were independent of the full-length pAkt2 protein (Supplementary Scheme S2 and Supplementary Table S3). The cleared library was then screened against pAkt2 incubated with **C**_**2**_**-N**_**1**_ to yield four potential biligand hits (Supplementary Table S4). These four biligands were synthesized (SI Figure S.8) and tested for binding to pAkt2 (Supplementary Figure S9). The best binder, **B**_**2,2,1**_, with linear **L**_**1**_ sequence kyyir, exhibited an EC_50_ of 13.6 nM (Supplementary Figure S10) and competed with the phospho-specific, commercial monoclonal antibody raised against the pS474 epitope for binding to the pAkt2 protein (Supplementary Methods, Supplementary Figure S11). **B**_**2,2,1**_ also exhibited selectivity for pAkt2 relative to inactive, non-phosphorylated Akt2. (Supplementary Figure S12).

### Chemical iteration of B_2,2,1_ to promote pAkt2 inhibition

Although phosphorylation of Ser474 can exert an allosteric influence on Akt2 kinase activity^29^, **B**_**2,2,1**_, designed to bind at the pSer474 site, did not inhibit pAkt2. We previously developed a linear ligand that, on binding to the Ser474 of pAkt2, modulated the kinase activity of pAkt2. Thus, we knew that affecting kinase modulation by ligand binding near this site was feasible^16^. We, therefore, explored chemical modifications of **B**_**2,2,1**_ that might promote pAkt2 inhibition while retaining high binding avidity. For this work, we drew from crystal structures of antibodies bound to phospho-peptide antigens^30^. That literature suggested that flexible amines, appropriately located, might increase binding to the phospho-serine residue. Appending diamino propanoic acid (Dap) at the N-terminal of **B**_**2,2,1**_ (to form **B**_**3,2,1**_, Fig. 1B) yielded a biligand with a similar affinity for pAkt2 (K_D_ = 12 nM by fluorescence anisotropy (Fig. 1C)), but also with an inhibitory influence over the kinase. An inhibition constant (IC_50_) of 8 µM was determined by monitoring the phosphorylation of GSK by pAkt2 (Supplementary Figure S13).

Virtually all envisioned uses of the target biligand require high target selectivity, so we tested for selectivity. **B**_**3,2,1**_ was shown to bind to pAkt2 and pAkt1, but not pAkt3 or inactive Akt2 (Fig. 1D). The sequence of the targeted pAkt2 epitope and the closely related pAkt1 and pAkt3 epitopes are provided in Fig. 1E. Notably, MK-2206, an allosteric inhibitor of Akt, is a similarly potent inhibitor of recombinant Akt1 and Akt2, but inhibits Akt3 five-fold less^31^. **B**_**3,2,1**_ also exhibited negligible binding to other kinases involved in the Akt pathway (GSK3 kinase) or related signaling pathways like the mTOR pathway (p70S6k kinase)^32^, BCR-ABL pathway (Abl2 kinase)^33^, and MAP kinase pathway (MEK1 kinase)^34^ (Fig. 1D).

### Chemical iteration of B_3,2,1_ to promote cell penetration

We next studied the cell-penetrating characteristics of the monoligand **C**_**2**_ and the biligand **B**_**3,2,1**_ in OVCAR3 cells. These cells are ovarian carcinoma cells that express high basal levels of Akt^35^. Cells were seeded into glass chambers, starved, and treated with 50 μM of Ly294002^36^ (a PI3K/Akt inhibitor^37^) to reduce the baseline level of pAkt2. Cells were then treated with epidermal growth factor (EGF) for 30 minutes at room temperature to induce phosphorylation of Akt. Non-induced cells were not treated with any growth factors. The cells were incubated with fluorescein-labeled **C**_**2**_ or fluorescein-labeled **B**_**3,2,1**_ and washed with buffer. Live imaging of stimulated and non-stimulated cells did not detect any fluorescence. Although stapled^38^ peptides have been reported to increase the cell penetration properties of certain helical peptides^39^, presumably due to the increased hydrophobicity of the alkenyl chain in the staple^40^, neither the dye-labeled stapled monoligand **C**_**2**_ nor the biligand **B**_**3,2,1**_ showed any evidence of cellular uptake. This negative result suggested that **C**_**2**_ and **B**_**3,2,1**_ were not cell-penetrant, and further modifications of **B**_**3,2,1**_ were required.

To optimize **B**_**3,2,1**_ for cell penetration, we first employed an alanine scan^41^ to identify the residues of **B**_**3,2,1**_ most essential for binding to pAkt. This was followed by evaluating a series of medicinal chemistry iterations designed to promote the rigidity and enhance the hydrophobic character of **B**_**i,j,k**_. The results of the alanine scan revealed that the three tyrosines and the glycine, all contained within the macrocycle moiety of **B**_**3,2,1**_, played essential roles in pAkt2 binding (Supplementary Figure S14). We also found that, while substituting the isosteric tyrosine analog 4-fluorophenylalanine (fl-F) for the tyrosines (Supplementary Figure S15) was well-tolerated (yielding the macrocycle moiety **C**_**4**_), N-methylation of the threonine and tyrosine residues to increase the rigidity of the cyclic peptide and promote passive cell penetration^8^ led to a total loss of binding to pAkt2 (Supplementary Figure S16).

There is no standard literature toolbox for inducing cell-penetrant characteristics into a non- penetrant peptide other than to add a cell-penetrating peptide (CPP) such as Tat^42^, which we previously demonstrated for a non-penetrating Akt ligand^43^. However, adding Tat can lead to issues such as the loss of membrane integrity and conversion of an innocuous fluorophore to a photolytic agent^44^. Thus, we chose not to pursue CPPs. Instead, we turned to techniques that have been reported for improving the cell-penetration properties of already cell-penetrant peptides^45^. Therefore, additional chemical modifications of the linker **N**_**j**_ designed to promote the helicity of **N**_**j**_ and potentially increase the cell penetration ability of **B**_**i,j,k**_^46^ were done by replacing **Nj** with a helical polyalanine linker^47^, *α/β* amino acids or an *α*, *α* -disubstituted *α*-amino acid^48^ containing linker (Supplementary Figure S17). These replacements and other established methods to increase cell permeability of peptides, such as PEGylation^49^, incorporating a positively charged arginine in **N**_**j**_^50^, and incorporating rigid aromatic residues in the linker^51,52^ (Supplementary Figure S18) led to significant loss of pAkt2 binding affinity and/or loss of pAkt2 inhibition (data not shown). Thus, the original linker from the in situ click screen (**N**_**1**_) appeared critical for binding, so the linker was retained almost intact. Therefore, it was only modified to incorporate the triazole formed during the in situ screen (**N**_**2**_) for the biligand syntheses.

**Fig. 2:**
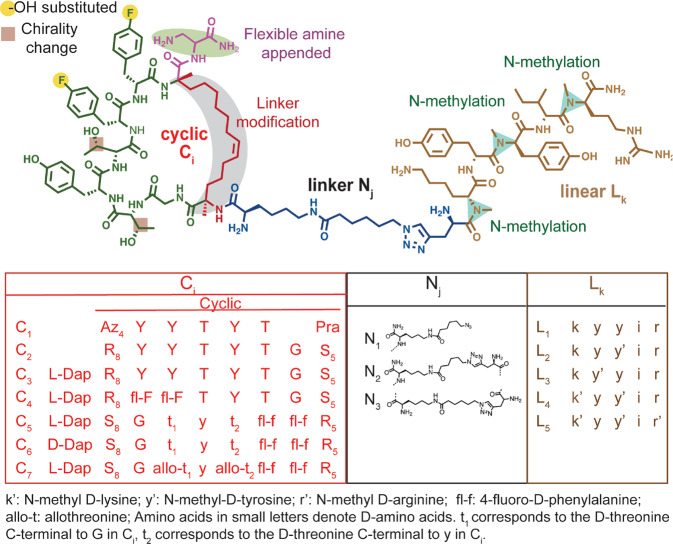
Biligand modifications to incorporate cell penetration characteristics. A. Structure of the optimized, cell penetrant biligand **B**_**7,3,5**_, with chemical iterations highlighted. **B**. List of the explored chemical iterations for the macrocycle (**C**_**i**_), the linker (**N**_**j**_) and the linear branch (**L**_**k**_). The original ring closing triazole linkage in **C**_**1**_ was replaced by a hydrophobic allyl linker (gray) in **C**_**2**_. Addition of the flexible amine (pink) at the N terminus of **C**_3_ increased phospho-selectivity and promoted pAkt2 kinase inhibition, while replacement of tyrosines by isosteric 4-fluorophenylalanine (4fl-F) (yellow) in **C**_**4**_ improved protease stability. **C**_**5-7**_ represent retro-inverso macrocycles.

The linear peptide arm **L**_**k**_ was modified using literature-guided approaches to increase rigidity and thus potentially improve passive cell penetration^53^. Double (**L**_**4**_) and triple (**L**_**5**_) N-methylated derivatives were found to be effective ligands (Supplementary Figures S19 and S20). For example, the biligand **B**_**4,2,5**_, which had three N-methylations in the linear L_k_ arm and two fl-F substitutions in the macrocycle (Supplementary Figure S21), retained much of the affinity for pAkt2 (EC_50_ = 60.6 nM, Supplementary Figure S22) that was shown by the original biligand **B**_**2,2,1**._ However, despite these modifications, **B**_**4,2,5**_, like its predecessors **B**_**2,2,1**_ and **B**_**3,2,1**_, was not cell-penetrant. A summary of the chemical modifications from **B**_**2,2,1**_ to **B**_**4,2,5**_ is presented in Fig. 2.

### Stereochemical modifications to promote cell penetration

MD simulations of **B**_**4,2,5**_ indicated a slightly hydrophilic character with a two-fold larger affinity to water (−15.9 ± 5.7 kcal/mol) relative to hexane (−8.3 ± 0.4 kcal/mol, Supplementary Figure S23). Thus, we turned to stereochemical modifications of the macrocycle, specifically partial and full retro-inversion, as a remaining toolkit for promoting cell penetration^44,45^. We further explored how the change in the stereochemistry of specific chiral centers in the ligand might influence cell penetration based on a report that chiral center addition was found to increase cell penetration^54^.

For cyclic peptides, retro-inversion, which involves inverting the sequence of the peptide and inverting the chirality of the amino acids of the peptide^55–57^ can maintain topochemical complementarity between the parent cyclic peptide’s side chains and those of its isomer^58^. A full retro-inversion converts the chirality of the amino acid backbone and the side chains and may improve protease stability^59,60^, while retaining the same similar binding site^56,57^. A partial retro-inversion converts the chirality of the backbone, but the side chains’ chiralities are selectively converted, thus providing an opportunity for tuning side-chain-solvent interactions. Retro-inversion of certain peptides has yielded improved cell penetration characteristics^61,62^, although partial retro-inversion also altered binding affinity^57,59,63,64^. Since biligand **B**_**4,2,5**_ had a disordered structure (from simulations), and binding activity was mainly associated with the side chains in the cyclic moiety (Supplementary Figure S14), **B**_**4,2,5**_ was a good candidate for retro-inversion. Based upon these precedents, we explored the retro-inversion of the cyclic moiety **C**_**4**_ to yield **C**_**5**_ (Supplementary Figure S24).

To accommodate this retro-inversion, the linker **N**_**2**_ was modified to **N**_**3**_ to link to the C terminal of **C**_**5**_ rather than the N terminal of **C**_**4**_. **B**_**5,3,5**_ contains the retro-inverso D cyclic component **C**_**5**._ It reproduces the correct topological orientation of the **C**_**4**_ macrocycle, presenting the side chains in the same spatial orientation relative to each other, although all the amino acids are of D chirality. MD simulations of **B**_**5,3,5**_ indicated a slight increase in hydrophobic character compared to **B**_**4,2,5**_, with a higher affinity (−9.1 ± 0.3 vs. −8.3 ± 0.4 kcal/mol) for hexane (Supplementary Figure S23), suggesting that retro-inversion might promote cell penetration.

A partial retro-inverso biligand, **B**_**7,3,5**_, differs from **B**_**5,3,5**_ in that the β-carbons on both macrocycle threonines have R rather than L chirality. (Supplementary Figure S25). Figure 2 summarizes all stereochemical modifications, starting from **B**_**4,2,5**_. Two additional retro-inverso biligands were prepared for cell penetration studies by modifying another chiral center at the C-terminal. **B**_**6,3,4**_ and **B**_**6,3,5**_ were designed with C terminal D-Dap rather than C-terminal L-Dap associated with **C**_**5**_. They also differ in the number of N-methylations in the linear component. All four retro-inverso biligands, consisting of D-amino acids, bound pAkt2 with EC_50_ values near 10 nM (Supplementary Figures S26, S27), and so, for this metric, all were superior to **B**_**4,2,5**_.

### Cell penetration assays

Visualization of cell penetration requires the addition of a dye to the ligand, which can influence the penetration itself. Thus, we first sought evidence of cell penetration through functional assays, in which live NIH-OVCAR-3 cells were treated with unmodified biligands and assayed for inhibition of pAkt2. Epidermal growth factor (EGF) stimulation of these cells leads to phosphorylation of Akt2. Inhibition of pAkt2 can be probed by assaying for the level of pGSK3, a downstream effector of pAkt2.

**Fig. 3:**
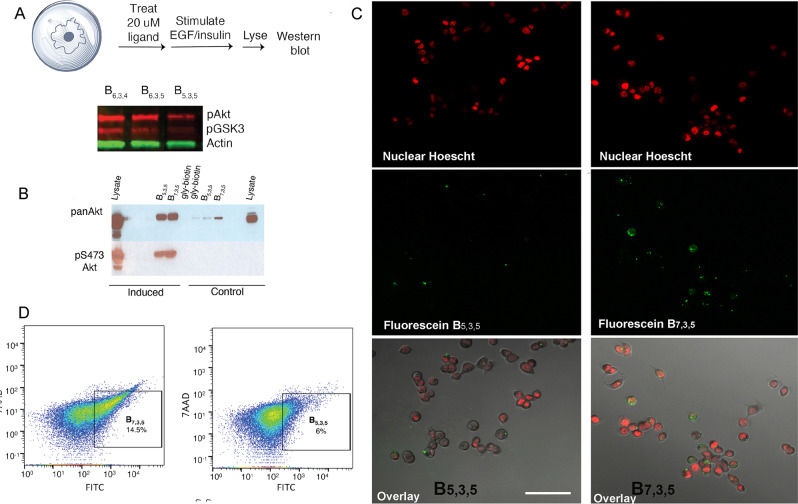
Cell penetration assays of selected retro-inverso biligands. **A**. Functional assay to interrogate for the inhibition of pAkt in stimulated NIH-OVCAR-3 cells by **B**_**6,3,4**_, **B**_**6,3,5**_, and **B**_**5,3,5**_. The levels of pAkt2 and its downstream effector, pGSK3 are assayed by western blotting of cell lysate. **B**. Use of **B**_**5,3,5**_ and **B**_**7,3,5**_ as phospho-specific antibody equivalents for pAkt2 immunoprecipitation. NIH-OVCAR-3 cells were serum starved, then induced with EGF or treated with a PI3K inhibitor Ly294002 (control). **C**. Fluorescence micrographs of stimulated NIH-OVCAR-3 cells treated with 100 nM of fluorescein-labeled **B**_**5,3,5**_ or **B**_**7,3,5**_, shown with 50-micron scale bar. The uppermost image shows the nuclear (Hoescht) stain, the middle images show treatment with fluorescein-labeled **B**_**5,3,5**_ or **B**_**7,3,5**_. Image overlay is shown at the bottom. **D**. Flow cytometry experiments of live, stimulated NIH-OVCAR-3 cells treated with **B**_**5,3,5**_ and **B**_**7,3,5**_.

Cells were serum starved and treated for 12 h at 37 °C with 20 μM unlabeled ligands **B**_**5,3,5**_**, B**_**6,3,4**_, and **B**_**6,3,5**_. The ligands were thoroughly washed off, and the cells were treated with EGF and lysed. Levels of pGSK3 were probed in the cell lysate using western blotting. Promising results were found only for the retro-inverso biligand **B**_**5,3,5**_ (Fig. 3A). Interestingly, **B**_**6,3,5**_, identical to **B**_**5,3,5**_ other than in the chirality of the C-terminal Dap, did not show any inhibition. These results suggested that the stereochemistry of each of the chiral centers could be functionally important.

We next explored how the chirality of the β-carbons of the two threonines in the cyclic component influenced cell penetration and pAkt2 inhibition by comparing **B**_**7,3,5**_ with **B**_**5,3,5**_. Recall that these biligands differ only by the R chirality in the β-carbons on both threonines. Both biligands exhibited excellent performance as the detection antibody equivalent in immunoprecipitation assays, selectively detecting pAkt2 in induced OVCAR3 cell lysate (Fig. 3B, Supplementary Figure S28). However, when dye-labeled variants of these biligands were tested, fluorescence micrographs showed that **B**_**7,3,5**_ exhibited significantly better penetration into stimulated OVCAR3 cells (Fig. 3C and 3D). Here, the cells were treated with 100 nM of dye-labeled **B**_**5,3,5**_ (left row) or **B**_**7,3,5**_. In addition to cytosolic penetration, distinct punctate formation for **B**_**7,3,5**_ was observed, suggesting that the peptide had penetrated the cytosol and had accumulated in endosomal vesicles, similar to what is seen for low concentrations of other cell-penetrating peptides^65^. For treated unstimulated cells, little penetration was observed, indicating low biligand retention within the cell when pAkt levels were low (Supplementary Figure S29).

### Mechanistic studies of cell penetration

**Fig. 4:**
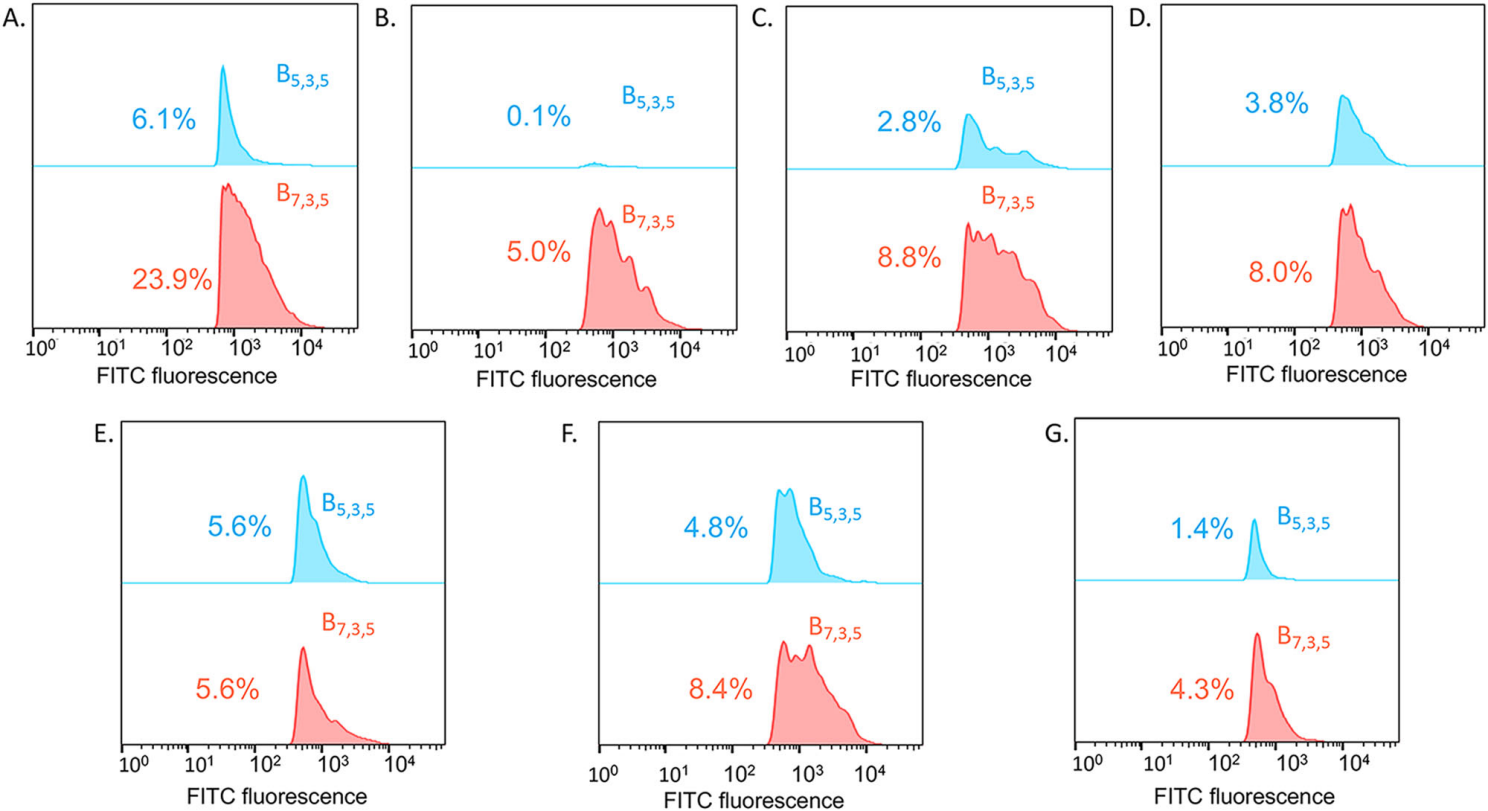
Determination of biligand cellular uptake mechanisms. **A** NIH-OVCAR-3 cells were treated with 200 nM fluorescein-labeled **B**_**7,3,5**_ and **B**_**5,3,5**_ at 37 °C. **B** Cells were treated with biligands at 4 °C to inhibit energy-dependent processes; **C** Cells were pre-treated with 2-deoxy-D-glucose to inhibit ATP uptake. **D**–**G**. NIH OVCAR3 cells were pre-treated with specific endocytosis inhibitors for 30 min. The inhibitors used were: 10 μM cytochalasin D (macropinocytosis, **D**), 4 μM filipin (caveolae-dependent endocytosis, **E**), chlorpromazine (clathrin-dependent endocytosis, **F**) or 5 mM methyl-β-cyclodextrin (lipid raft-mediated endocytosis, **G**). Cells were then treated for 8 h with 200 nM fluorecein-labeled **B**_**7,3,5**_ and **B**_**5,3,5**_. Following the incubation, cells were washed, trypsinized, and sorted using flow cytometry.

We explored the mechanism of cell penetration of **B**_**7,3,5**_ and **B**_**5,3,5**_ into NIH-OVCAR-3 cells using approaches designed to test for uptake by energy-independent passive-diffusion pathways and energy-dependent endocytosis processes^66^. To determine the role of energy-independent pathways, cells were either incubated with FITC-labeled ligands for 8 h at 4 °C or incubated with 2-deoxy-D-glucose (*deoxyG)*^67^ for 1 h, then treated with peptide for 8 h at 37 °C. Cellular uptake of both **B**_**7,3,5**_ and **B**_**5,3,5**_, which were 14% and 6%, respectively, at 37 °C (Fig. 4A), was inhibited at 4 °C (Fig. 4B), suggesting thermally activated transport into the cell is essential for both biligands. Interestingly, **B**_**7,3,5**_ uptake is reduced by 80%, whereas **B**_**5,3,5**_ uptake is almost entirely inhibited at 4 °C, suggesting that **B**_**7,3,5**_ cellular uptake may partly proceed via an energy-independent pathway or that the barrier for penetration of **B**_**7,3,5**_ is lower relative to that for **B**_**5,3,5**_. Similarly, for cells pretreated with *deoxyG* to inhibit ATP uptake, cell penetration by both biligands is significantly inhibited **(**Fig. 4C), confirming that endocytosis is involved in the cellular uptake process.

To identify the specific endocytosis mechanisms, NIHOVCAR-3 cells were treated with known inhibitors of macropinocytosis, caveolae-dependent endocytosis, clathrin-dependent endocytosis, and lipid-raft mediated endocytosis pathways before incubating the cells with ligands. Cells are treated with 10 μM cytochalasin D (*cyto D*), 4 μM filipin (*filip*), 10 μM chlorpromazine (*chlor*) or 5 mM methyl-β-cyclodextrin (*MβCD*), respectively, for 30 minutes^67^ and then incubated with 200 nM FITC-labeled ligand for 8 h at 37 °C. As shown in Figure 4D–F, **B**_**7,3,5**_ uptake is reduced to a larger extent than **B**_**5,3,5**_ by *cyto D* (71% vs. 46% reduction), *filip* (81% vs. 25% reduction) and *chlor* (67% vs. 33% reduction). The uptake of **B**_**7,3,5**_ and **B**_**5,3,5**_ was inhibited most on pretreatment of the cells with *MβCD*, a lipid-raft-dependent endocytosis inhibitor (Fig. 4G). This indicated that lipid-raft-mediated endocytosis is a significant endocytosis mechanism for **B**_**7,3,5**_ and **B**_**5,3,5**_. Thus, both **B**_**7,3,5**_ and **B**_**5,3,5**_ were uptaken through various endocytosis mechanisms, with lipid-raft-mediated endocytosis providing a significant mechanism for both biligands.

### Molecular dynamics simulations of biligand endocytosis

**Fig. 5:**
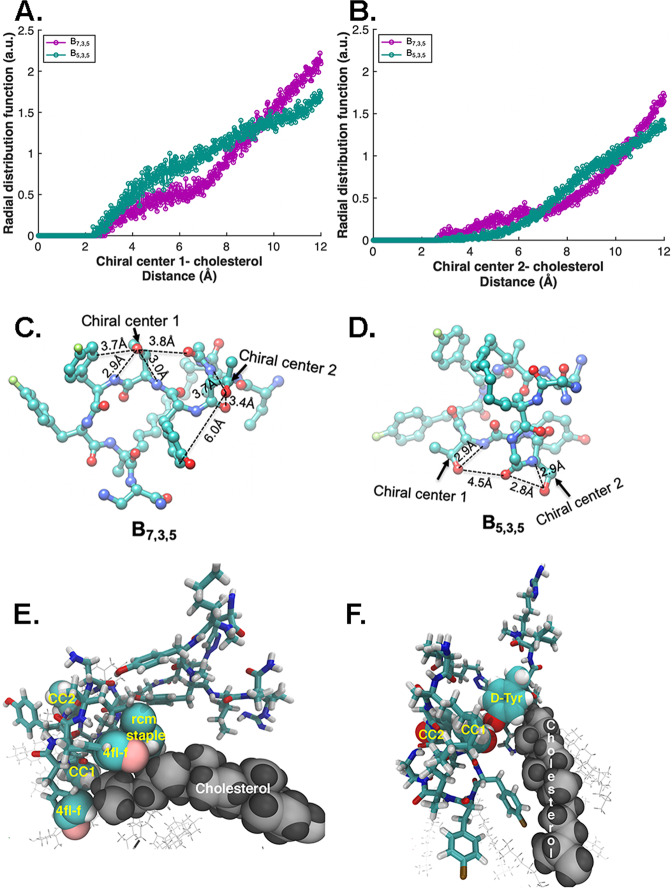
Cholesterol interactions with the enantiomeric threonine residues on B_7,3,5_ and B_5,3,5_. **A, B**. The integrated radial distribution function (RDF) analysis near the transition state of CC1 and CC2 with cholesterol for both biligands. **C, D**. The optimized conformation of chiral centers of **B**_**5,3,5**_ and **B**_**7,3,5**_ near the mid-membrane transition state obtained from long-timescale (an aggregate of ~34 μs) metaMD simulations. **E, F**. Additional B_5,3,5_-cholesterol interactions recruited by CC1. **E**. CC1, together with the two 4Fl-Phe, and the *rcm* staple in **C**_**5**_ form a nest where a cholesterol binds through hydrophobic interactions. **F**. The hydroxyl side chain of Thr in CC1 formed a hydrogen bond with the phenolic oxygen of D-Tyr in the linear **L**_**5**_ arm of **B**_**5,3,5**_, that oriented the D-Tyr to interact favorably with cholesterol. Molecular conformations are shown near the transition state during **B**_**5,3,5**_ cell penetration. **B**_**5,3,5**_ is shown in blue, with cholesterol interacting with B_5,3,5_  shown as (grey) van der Waals spheres. Cholesterol interacting with **B**_**5,3,5**_ is shown in (grey) VDW representation.

While the results of Fig. 4 indicate that multiple factors likely influence the cell penetration characteristics of these biligands, the importance of lipid-raft-mediated endocytosis suggested that specific biligand-membrane molecular interactions might provide a mechanism that is most easily influenced through the stereochemical tuning of the interactions. We thus conducted molecular dynamics (MD) simulations to probe for a molecular mechanistic understanding of this difference (Fig. 5)_._ Since these two biligands differ only in the stereochemistry of the β-carbons on the two threonine residues in the macrocycle, we paid particular attention to how these residues interacted with membrane components. In Fig. 5, the side chains of allo-t_1_ and allo-t_2_ of **C**_**7**_ (t_1_ and t_2_ of **C**_**5**_) are annotated as the chiral centers CC1 and CC2, respectively.

The differential cell penetrating characteristics of ligands **B**_**5,3,5**_ and **B**_**7,3,5**_ was explored through well-tempered metaMD simulations^68^ using periodic boxes containing one biligand, ~6500 water molecules, the biological level of NaCl (0.15 M ≅ 34 ions), a physiological pH of 7.4, and a lipid bilayer containing 35 POPC molecules in each leaflet with 20% (7 molecules) cholesterol. The total number of atoms in the simulation was ~30,000. Although this calculation addressed only the case of passive diffusion directly, we hypothesized that it might reveal how relatively minor changes in stereochemistry can influence energy barriers to cell penetration.

To prepare the system for free energy calculations, we first minimized the simulation box using the steepest descent algorithm with positional restraints on heavy atoms while allowing the POPC/cholesterol molecules to move freely along the xy-plane. We then gradually (over two ns) removed restraints on the biligand, POPC, and cholesterol and performed a ~ 100 ns equilibration to relax the simulation box. To evaluate the energetics associated with the solvation of **B**_**5,3,5**_ and **B**_**7,3,5**_ on the membrane surface, we employed metaMD free energy calculations for ~3 μs (Supplementary Figure S30). We found that the two biligands exhibited similar affinity for the membrane bilayer (ΔG = −5.0 ± 0.1 kcal/mol) suggesting that their differences in cell penetration  were exclusively associated with transmembrane diffusion.

For the transmembrane diffusion free energy calculations, we modified the protonation state of the residues to be appropriate for a low dielectric constant medium by adjusting the net charge of each biligand to its neutral state. We started with the optimum position of biligands on the membrane surface and determined from the free energy calculations at the solution surface, and applied a potential across the membrane to promote transmembrane diffusion (Supplementary Figure S30). Then, to examine transmembrane migration of either **B**_**5,3,5**_ or **B**_**7,3,5**_, we performed an aggregate of ~34 μs metaMD free energy calculations (Supplementary Figure S30c) to evaluate the energy barrier (ΔG^☨^) of diffusion across the membrane for this particular pathway (Supplementary Figure S30c), since that barrier controlled the diffusion rate (diffusion coefficient ∝ exp(-ΔG^☨^/RT)). We found that the free energy barrier to diffuse from one membrane surface slightly favored **B**_**7,3,5**_ (ΔG^☨^ = 22.2 ± 0.3 kcal/mol) relative to **B**_**5,3, 5**_ (ΔG^☨^ = 22.5 ± 0.1 kcal/mol), which, while barely different, was consistent with our independently determined flow cytometry results that indicated **B**_**7,3,5**_ penetrated 2-3 times faster than **B**_**5,3,5**_ at 37 °C.

To identify an atomistic mechanism by which the chirality might influence diffusion, we explored several possible descriptors (Supplementary Figure S31), with the only significant differential relating to the interactions between CC1 and cholesterol. In Fig. 5A, we present the integrated radial distribution surrounding CC1 out to 7.5 Å. We found that CC1 on **B**_**5,3,5**_ interacted with ~4.0 atoms of cholesterol molecules within the lipid membrane, whereas CC1 on **B**_**7,3,5**_ interacted with ~2.5 atoms of cholesterol molecules within the lipid membrane. CC2, by contrast, exhibited little differential effect (Fig. 5B). In fact, for CC1, analysis of the non-bonded interaction energies revealed a delicate balance in interactions between the macrocycle and cholesterol molecules, with **B**_**7,3,5**_ exhibiting ~3 kcal/mol stronger interactions with cholesterol (Supplementary Figure S31b). However, these stronger interactions arose mainly from the body of the macrocycle rather than the chiral centers.

We explored the atomistic details of the biligand-cholesterol interactions within the transmembrane region (Fig. 5C and 5D). This analysis revealed that **B**_**7,3,5**_ adopted conformations that allowed CC1 to enhance internal polar interactions, making them less available to interact with the cholesterol. On the other hand, **B**_**5,3,5**_ adopted conformations that exposed the polar residues to cholesterol, resulting in additional interactions, two of which are illustrated in Figs. 5E and 5F. The net result was a decrease in the diffusion rate of **B**_**5,3,5**_ relative to **B**_**7,3,5**_.

## Discussion

The pS474 region of Akt2 is disordered and not well-resolved in high-resolution crystal structures. Similarly, disordered loops are known to play critical allosteric roles in activating intracellular kinases^69^. However, targeting such disordered regions is challenging for both small molecules (no clear binding pocket) and antibodies (no cell penetration). While peptides have the potential to bridge this gap, clear guidelines have yet to be established for developing cell-penetrant, potent inhibitors. This contrasts with the use of Lipinski’s rules^70^, which provide such guidelines for small molecule drug development. In this article, we executed a workflow to demonstrate, for a single case, how a relatively large peptide comprised of a macrocyclic peptide linked to a linear peptide can be engineered into a cell-penetrant kinase inhibitor and can be utilized as an in situ analytic tool for probing specific kinase activity. While a single case study does not provide a general pathway, the approach described here can serve as an essential benchmark of what can be done when considering an expanded synthetic and computational toolset for peptide optimization.

Here we utilized the screening method of chemical epitope targeting, followed by iterative in situ click chemistry screens, to identify a peptide biligand with binding specificity for the phospho-Ser474 epitope of Akt2. The initially developed biligand comprised a peptide macrocycle linked to a linear peptide branch. It was a selective binder to pAkt2 but was neither an inhibitor nor cell-penetrant. Next, literature peptide techniques and phospho-specific antibody crystal structure data were mined to guide the strategic use of N-methylations and the addition of an N-terminal flexible Dap to the macrocycle moiety. These steps yielded the **B**_**4,2,5**_ biligand with improved inhibitory characteristics but lacked cell-penetrant character.

A significant finding of this work was that stereochemical modifications, guided by molecular dynamics simulations, could be harnessed to fine-tune the biligand for cell penetration while retaining its functional characteristics against pAkt2. The first stereochemical modification (yielding **B**_**5,3,5**_) involved retro-inversion of the macrocycle variable region. This change produced a calculated increase in the hydrophobic character of the biligand and led to measurable cell penetration. The second stereochemical modification was to switch the chirality of the β-carbons on the two threonine residues within the macrocycle, yielding **B**_**7,3,5**_. These seemingly slight chemical changes did not alter the hydrophobicity of the biligand but did yield an approximately 3-fold increase in cell-penetrant character. Our meta-molecular-dynamics simulations allowed us to trace much of this increase to a reduction of in-membrane biligand-cholesterol interactions, thus effectively lowering the barrier (by 0.03 eV) to transmembrane diffusion.

The optimized **B**_**7,3,5**_ biligand, and/or the 2-fold less cell-penetrant stereoisomer **B**_**5,3,5**_, performed well in applications traditionally designated exclusively for either antibodies or small molecules, including as capture agents for immunoprecipitation, as reagents for live cell imaging of kinase activation, and as kinase inhibitors. This provides the proof of principle that an approach that draws from relatively straightforward peptide chemistries and stereochemistries integrated with large-scale meta-molecular dynamics may ultimately yield a class of molecules that combine the best characteristics of both antibodies and small molecules.

## Methods

### Solid phase peptide synthesis

Peptides were synthesized on Rink Amide MBHA, Biotin Novatag, Biotin PEG Novatag, and Sieber Amide resin either manually or on the Titan 357 Automatic Peptide Synthesizer (AAPPTec, Louisville, KY). Amino acid solutions in NMP (2 equivalents), with 2 equivalents of HATU and 6 equivalents of DIEA, were used for the amino acid coupling reaction. For the removal of Nα-Fmoc protecting groups, a solution of 20% piperidine in DMF was used. Further synthesis protocol details are provided in Supplementary Methods.

### HPLC purification of peptides

All the peptides were purified using a gradient of double distilled water and HPLC grade acetonitrile and 0.1% TFA on the RP-HPLC (Beckman Coulter System Gold 126 Solvent Module and 168 Detector) using a C18 reversed phase semi-preparative column (Phenomenex Luna 10 µm, 250 × 10 mm).

### Comprehensive CuAAC cyclized peptide library synthesis^5^

Randomized OBOC libraries of hexameric peptides were synthesized using the Titan 357 Automated Peptide Synthesizer (AAPPTec) on 90 µm polyethylene glycol-grafted polystyrene beads (TentaGel-S NH2, 0.28 mmol/g, 2.86 ×106 beads/g). A comprehensive linear peptide library was synthesized on Tentagel-S NH2 resin using the standard SPPS library synthesis method. Each library was synthesized at a 10-fold excess to ensure adequate representation of each library element. After synthesis of the linear library, all the beads in the linear library were subjected to an on-bead CuAAC reaction for 6 h at room temperature with 1.5 equivalents of CuI, 2.5 equivalents of ascorbic acid in 20% piperidine in DMF. After washes to remove the adsorbed Cu, the library was washed with DMF, methanol, and DCM and dried. Random beads were picked from the library to be sequenced. The rest of the library was stored in NMP. The structure and details of the synthesized library A are provided in Supplementary Methods.

### Screening cyclic OBOC library with peptide epitope

For isolating cyclic ligands against the peptide epitope, the cyclic peptide library A was screened, first against the scrambled peptide (prescreen) and then against the target phosphopeptide (epitope screen). A schematic of the chemical epitope screen is provided in Supplementary Scheme S1, and the detailed protocol is provided in Supplementary Table S1.

### Screening: Prescreen^5^

Briefly, 500 mg of the library was dried with DCM and, afterward, swelled and shaken overnight in the binding buffer (0.1% BSA, 25 mM trisCl, pH = 7.5, 150 mM NaCl, 0.05% Tween20) at room temperature. Beads were incubated overnight, with a solution of 500 nM of biotin-tagged scrambled peptide in binding buffer at room temperature. The beads were washed three times with the binding buffer. Next, they were treated for 1 h at room temperature with a 1:10,000 dilution of mouse monoclonal Anti-biotin antibody conjugated with Alkaline Phosphatase (mAb-AP) (Sigma, A6561) in binding buffer. Following the screen, the beads were successively washed three times with each wash buffer 1(25 mM trisCl, pH = 7.5, 150 mM NaCl, 0.1% BSA, 0.05% Tween20); wash buffer 2 (25 mM trisCl, pH = 7.5, 150 mM NaCl, 0.05% Tween20) and AP buffer (100 mM Tris-HCl, pH 9.0, 150 mM NaCl, 1 mM MgCl_2_). The library was treated with a 5-bromo-4-chloro-3-indolyl phosphate (BCIP) solution (33 µL BCIP in 10 mL AP buffer) for fifteen minutes. The AP reaction was stopped using 0.1 M HCl to denature the alkaline phosphatase enzyme and stop the chemical reaction with BCIP. Blue background hits were removed from clear ones using a pipette manually. Clear beads were combined and transferred to a peptide synthesis vessel. The beads were shaken in 7.5 M of guanidine-HCl (GuHCl) pH = 2 to denature the antibody for 2 h at room temperature. Afterward, the library was washed with deionized water and treated with NMP to decolorize the blue beads. Following a wash with DCM, the clear beads were dried, then shaken overnight in the binding buffer.

### Screening: Epitope screen

The beads were screened overnight with a solution of 500 nM of the biotin-tagged target phospho-peptide in binding buffer at room temperature. Following washes with the binding buffer, the beads were treated with a solution of mAb-AP. The same procedure as described for  the prescreen was conducted to obtain blue hits. The beads were manually picked, washed with 7.5 M GuHCl and water, and sequenced using the Edman Sequencer. The sequences from the epitope screen are given in Supplementary Table S2.

### Screen for biligand

A schematic for the biligand screen is given in Supplementary Scheme S2, and further details are provided in Supplementary Table S3.

### Biligand screen: Prescreen

A total of 500 mg of library B (D-Pra -XXXXX- Met – TG) (Supplementary Scheme S2) were washed in water and swelled overnight in the binding buffer. A total of 50 μM solution of anchor (Supplementary Fig. S7) was added to the beads and shaken for 2 h at 4 °C. The screened beads were treated with 7.5 M guanidium hydrochloride (GuHCl), pH 2.0 for 2 h, washed with double distilled water (ddH_2_O). The beads are incubated with buffer for 8 h. The beads were then treated with mAb-AP and washed, following the protocol described in the prescreen above. The blue hit beads, background binders to the anchor peptide, or the detection antibody was picked up manually. The clear beads were stringently washed and used in the product screen.

### Biligand Screen: Product screen^5,16^

The washed beads from the prescreen were dried and swelled overnight in the binding buffer in an 8 ml fritted polypropylene solid-phase synthesis tube. In an Eppendorf tube, a 50 μM solution of anchor peptide was incubated for thirty minutes at 4 °C with 50 nM full-length Akt2 protein in the binding buffer. The solution was added to the beads and shaken at 4 °C for 2 h. The steps of the prescreen described above were followed. The sequences from the biligand screen are given in Supplementary Table S4.

### ELISA for peptides against full-length pAkt2 protein^5,16,71^

A total of 2 μM solution of each biotinylated peptide was prepared in binding buffer. A total of 100 μL of each solution was added to each well of a High-Capacity Streptavidin 96 well plate (Thermo Scientific), and the plate was shaken for 2 h at room temperature. The wells were blocked with 5% milk in 25 mM tris chloride, pH = 7.4, 150 mM NaCl and 0.05% Tween 20 (TBST). The wells were washed with binding buffer five times. Serial dilutions of active His6-tagged Akt2 were made in the binding buffer. A total of 100 μL of each solution was added per well, and the plate was shaken overnight at 4 °C. The wells were washed three times with the binding buffer, then treated for an hour with a 1:1000 dilution of anti 6X His mouse monoclonal [HIS.H8] antibody. A 1:10,000 dilution of goat anti-mouse antibody-Horse Radish Peroxide conjugate in binding buffer was added to the wells for an hour. The plate was washed three times with TBST and once with TBS (25 mM tris chloride, pH = 7.5, 150 mM NaCl), treated with TMB substrate (KPL), and quenched with sulfuric acid, and the absorbance at 450 nm wavelength measured. From the absorbance values at 450 nm wavelength (A450) for each protein concentration, the corresponding blank A450 (no ligand immobilized, same concentration of protein added) was subtracted. They were then fitted by non-linear regression in GraphPad Prism 6.

### Epitope/protein selectivity assay^5,16,71^

A total of 1.25 μM biotinylated ligand was prepared by diluting the 1 mM stock in binding buffer (0.1% BSA, 25 mM trisCl, pH = 7.5, 150 mM NaCl, 0.05% Tween20). The prepared ligand solution or 0.125% DMSO (buffer control) in binding buffer was immobilized on a High-Capacity Streptavidin 96 well plate (Thermo Scientific). After washing the excess ligand, the plate was blocked overnight with 1% BSA in TBST. 2.5 μM solutions of the His_6_-tagged target phosphopeptide epitope, Akt2 amino acids 450–481, or His_6_-tagged scrambled peptide was added to each of the wells and incubated overnight. After three washes with the binding buffer, the plate was treated for an hour with a 1:1000 dilution of anti-His6 mouse monoclonal antibody in the binding buffer. A 1:10,000 dilution of goat anti-mouse antibody conjugated to Horse Radish Peroxide in binding buffer was added to the wells. The plates were washed four times, five minutes each, with TBST and once with TBS. The color was developed by adding TMB substrate to each well. The reaction was quenched with 0.5 M H_2_SO_4_. The absorbance values at 450 nm (A450) were measured on a 96-well plate reader. The Net A450 was obtained by subtracting the absorbance value for the blank control (no immobilized ligand) from each triplicate value obtained for the ligand–epitope interaction. The selectivity assay with full-length His_6_ tagged active Akt1, Akt2, and Akt3, and inactive Akt2 was performed following the same protocol, using 25 nM protein instead of 2.5 μM His_6_ tagged peptide epitope.

### Fluorescence anisotropy binding assay^5,71^

A total of 50 nM fluorescein-conjugated peptide was incubated with serial dilutions of Akt2 protein in the binding buffer at room temperature. Binding activity at 40 min was measured by fluorescence anisotropy using Flexstation 3 Multi-mode Plate Reader (Molecular Devices). K_D_ values for FITC-labeled peptide binding to pAkt2 were determined by nonlinear regression analysis using the standard anisotropy equation in Prism software 6.0 (GraphPad).

### Inhibition experiments with unlabeled biligands

Approximately 1 million NIH-OVCAR-3 cells (ATCC, from passage 3) were plated in each well of an 8-well cell culture plate with RPMI-1640 media containing 10% fetal bovine serum and 1% penicillin/streptomycin. After 24 h for cell confluence, the wells were starved for 3 days by placing them in RPMI-1640 media with 1% penicillin/streptomycin. The wells were treated with 20 µM ligands RPMI-1640 with 1% penicillin/ streptomycin. After 24 h, cells were stimulated with EGF for 15 minutes, washed with PBS, and lysed. Cells were lysed with cell lysis buffer (10 mM Tris-Cl (pH = 7.5), 100 mM NaCl, 1% (v/v) Triton X-100, 0.1% SDS (w/v), 0.5% deoxycholate, 1 mM DTT, 1 mM EDTA, 1X PhosStop phosphatase inhibitors (Roche), 1X Complete protease inhibitors (Roche)). The amount of protein from each cell was measured using BCA Protein Assay Kit (Pierce Biotechnologies, Inc). The volume of each lysate was adjusted to contain 10 μg of total protein, and the volume was made up to 50 μl with Laemmli buffer. The solutions were denatured by heating at 95 °C for 5 min. After cooling to room temperature, lanes of a 12-well 12% gel (Biorad) were loaded with 10 µl of each sample. The electrophoresis was run for 1 h 40 min at 90 volts. The gel was transferred to a nitrocellulose membrane by the semidry transfer method at 15 volts for 30 min. The nitrocellulose membrane was blocked for 2 h at 4 °C with 5% non-fat milk/TBST and washed thrice with TBST. The nitrocellulose membrane was incubated and treated overnight at 4 °C with a 1:250 dilution of pGSK3B (Ser9) rabbit antibody (Cell signaling technology, 5B3), a 1:2000 dilution of pS473 Akt rabbit antibody (Cell signaling technology, D9E), and 1:1000 dilution of an actin mouse antibody (Cell signaling technology, 8H10D10) respectively in 0.5% non-fat milk in TBST. The membranes were washed and treated for an hour with a 1:10,000 dilution of Alexa 790 conjugated goat anti-rabbit antibody (Abcam, ab186697) and a 1:10,000 dilution of Alexa 700 conjugated goat anti-mouse antibody (Invitrogen, A21036) in 0.5% milk/TBST. After five washes of five minutes, each with TBST and one wash of five minutes with TBS, the nitrocellulose membrane was imaged using Typhoon FLA 9000 imager.

### Live cell imaging

For cell imaging experiments, 1 million NIH-OVCAR-3 cells (ATCC, from passage 3) were plated in each well of an 8-well cell culture plate with RPMI-1640 media containing 10% fetal bovine serum and 1% penicillin/streptomycin (P/S). Following confluence to 80%, cells were starved by placing them in RPMI-1640 media with 1% penicillin/ streptomycin and no phenol red for 3 days. The wells were treated with 50 μM PI3K/Akt inhibitor Ly2940002 inhibitor for 45 minutes to inhibit the Akt pathway in all cells. The cells were washed with PBS following the treatment. For EGF-stimulation of cells, specific wells were treated with 400 ng/ml EGF in RPMI media (no phenol red) containing 10% FBS and 1% P/S for 15 min. All wells were treated with 100 nM fluorescein-labeled peptides in RPMI medium with 1% P/S (no phenol red) for 16 h. On the day of imaging, the cells were washed in PBS two times, then treated with Hoechst 33342 (Invitrogen) following the manufacturer’s protocol. All cells were imaged in a 5% CO_2_ incubation chamber using Zeiss LSM 710 microscope.

### Using biligands as immunoprecipitation reagents^16^

NIH-OVCAR-3 cells, passage 4, were plated on a cell culture flask and grown to confluence over 24 h in RPMI-1640 with 1% P/S and 20% FBS. For EGF stimulation, one flask was treated with 50 ng/ml EGF in RPMI-1640 with 1% P/S and 20% FBS for 30 minutes. For non-stimulated control cells, OVCAR3 cells were treated with 50 μM Ly2940002 inhibitor in RPMI-1640 with 1% P/S for 2 h. The flasks were washed with PBS and lysed using a cell scraper and cell lysis buffer, and the total protein concentration of the lysate was measured using the BCA assay. A total of 300 μl of each lysate solution was made, adjusting the protein concentration so that the final concentration was 0.67 mg/ml.

To immobilize the ligands, streptavidin-agarose resin solution (EMD) was swelled in TBST (25 mM tris chloride, pH= 7.5, 150 mM NaCl, 0.05% Tween-20). Each biotinylated ligand was immobilized on the Streptavidin-agarose resin by adding 7.2 μL of 1 mM ligand stock (DMSO) to 10 μL of the swelled streptavidin-agarose resin. For the blank control, the resin was treated with 7.2 μL of 1 mM biotinylated acyl glycine. After shaking overnight at 4 °C, 50 μM D-biotin was added to the resin to block any remaining sites. The resin was washed with TBST five times, for fifteen minutes each. The resin was then swelled in the binding buffer for 2 h. A total of 300 µl lysate of EGF-stimulated NIH-OVCAR-3 cells or control non-stimulated cells was added to each immobilized ligand. The tubes were shaken at 4 °C for 18 hours. The beads were washed three times for fifteen minutes in binding buffer, three times (fifteen minutes) in TBST, and three times (fifteen minutes) in TBS to remove unbound proteins. The resin-bound proteins were eluted by adding 40 μL of 2X SDS-PAGE sample loading buffer (BioRad) and heating at 95 °C for five minutes. A 1:10 diluted lysate sample in sample loading buffer was prepared by mixing 1 μL of the sample with 9 μL sample loading buffer and denaturing the sample by heating it at 95 °C for five minutes. 10 μL of each sample was loaded on a 12% SDS-PAGE gels (BioRad) and run for 80 minutes at 110 volts. The gel was transferred to nitrocellulose membrane by the semidry transfer method, blocked for two hours at 4 °C with 5% non-fat milk, and treated overnight at 4 °C with a 1:1000 dilution of pan-Akt rabbit monoclonal antibody and a 1:2000 dilution of pS473 Akt rabbit monoclonal antibody, respectively, in 0.5% non-fat milk. The membranes were washed and treated for an hour with a 1:10,000 dilution of a secondary monoclonal mouse anti-rabbit- HRP antibody in 0.5% milk. After five washes of five minutes each with TBST and one wash of five minutes with TBS, the blots were developed with West Dura ECL substrate (Thermo Scientific) and imaged on film.

### Flow cytometry

1 million NIH OVCAR3 cells, passage 3, were plated on wells of an 8-well cell culture flask and grown to confluence over 24 hours in RPMI-1640 with 1% P/S and 20% FBS. The cells were stimulated by treatment with 50 ng/ml EGF in RPMI-1640 with 1% P/S and 20% FBS for 15 min. The flask was once washed with RPMI-1640 (no phenol red) with 1% P/S and 5% FBS, then treated with 200 nM fluorescein-conjugated peptide in RPMI-1640 (no phenol red) with 1% P/S and 5% FBS for 8 hours in 5% CO_2_ chamber at 37 °C. The wells were washed with PBS to remove excess peptides. The wells were treated with 0.05% trypsin and accutase in RPMI-1640 (no phenol red) containing 5% FBS and 1% P/S for 20 minutes at 37 °C. Following the dissociation of the cells from the flask, the cells were spun down and then suspended in 2 ml FACS buffer (1X PBS (Ca/Mg free), 1 mM EDTA, 25 mM HEPES, 1% FBS, 1 mM MgCl_2_ and DNAase I (Roche)) in FACS tubes with cell strainer cap. 7AAD solution was added to the buffer before the flow cytometry analysis. Using 7AAD as an indicator, live cells were separated from dead cells in flow cytometry, and the live cells were analyzed for fluorescent peptide uptake.

For the inhibition experiments, NIH-OVCAR-3, passage 2, cells were plated and made confluent in 2 separate 8-well cell plates. Following EGF treatment and wash with RPMI-1640 (no phenol red) with 1% P/S and 5% FBS, individual wells were treated for 30 min at 37 °C with either 10 μM cytochalasin D, or 4 μM filipin, or 10 μM chlorpromazine, or 5 mM methyl-β-cyclodextrin or treated for an hour with 6 mM 2-deoxy-D-glucose for 1 h. Fluorescein-labeled peptides were added to both plates. After washing with PBS, the plate with inhibitors was incubated at 37 °C for 8 h, while the other 8-well plate was kept at 4 °C for 8 h. Following the incubation, cells were washed with PBS and dissociated from the flask, as described earlier, with trypsin and accutase treatment, and suspended in 2 ml FACS buffer (diluted 10X Hanks balanced buffer without phenol red, 0.5% BSA fraction V, 25 mM HEPES, 1 mM MgCl_2_ and DNAse 1) in FACS tubes with cell strainer cap. 7AAD was added before flow cytometry analysis.

For the data analysis, following the gating of NIH OVCAR3 cells in FSC vs. SSC dot blot, the populations were further gated for the elimination of cell dimers and aggregates (Supplementary Figure S32). Gating of FITC+ cells was based on the staining of 7AAD only stained cells control and unstained cells control. The percentage of FITC+ cells was based on the frequency of the total.

### Metamolecular dynamics simulations

Using the CHARMM–GUI interface^72,73^, we built a periodic simulation box containing one biligand (**B**_**5,3,5**_, or **B**_**7,3,5**_), water ( ~ 6500 molecules), physiological NaCl (0.15 M ≅ 34 ions), and a lipid bilayer containing 35 POPC molecules in each leaflet with 20% cholesterol content (7 cholesterol molecules in each leaflet). For free energy calculations, POPC, cholesterol, and ions were described by the Charmm36m parameter set^74^. Water was described using the CHARMM TIP3P model. The ligand was parameterized using the ParamChem server^75,76^.

In our well-tempered metaMD simulations^68^, the temperature was maintained at 310 K using a velocity-rescale^77^ thermostat with a damping constant of 1.0 ps for temperature coupling while the pressure was controlled at 1 bar using a Parrinello-Rahman barostat algorithm^78^ with a 5.0 ps damping constant for the pressure coupling. Semi-isotropic pressure coupling was used during this calculation. The Lennard-Jones cutoff radius was 12 Å, where the truncated non-bonded forces at the cutoff distance was smoothly shifted to 0 after 10 Å using a force switch function. Periodic boundary conditions were applied to all three directions. The Particle Mesh Ewald algorithm^79^ with a real cutoff radius of 10 Å and a grid spacing of 1.2 Å was used to calculate the long-range coulombic interactions. A compressibility of 4.5 × 10^−5^ bar-1 was used to relax the box volume in the xy- plane and for the z-axis. In all the above simulations, water OH-bonds were constrained by the SETTLE algorithm^80^. The remaining H-bonds were constrained using the P-LINCS algorithm^81^. All simulations were carried out using GROMACS^82^ and metaMD calculations were done using PLUMED-2^83^.

### Solution-mechanism

To prepare the system for free energy calculations, we first minimized the simulation box using the steepest descent algorithm with positional restraints on heavy atoms while allowing the POPC/cholesterol molecules to move freely along the xy-plane. We then gradually removed restraints on the biligand, POPC, and cholesterol and performed a ~ 100 ns equilibration to relax the simulation box. To prepare 32 different configurations for our subsequent metaMD free energy calculations, we gradually pulled the biligands towards the membrane surface over 10 ns by applying the force constant of 0.48 kcal mol^-1^ Å^−2^ with a pull rate of 0.005 Å ps^-1^. The forces were exerted on the following two collective variables, which later were used to impose the metaMD biases forces on them as well.

CV1. The z-component of the distance between the center of mass of the macrocycle to the center of mass of the lipid membrane,

CV2. the distance between the center of mass of the linear branch to the center of mass of the lipid membrane.

Subsequently, we extracted 32 different windows/configurations and subjected them to 5 ns simulation to relax the simulation box. These relaxed 32 windows were later used as initial configurations for our well-tempered metaMD calculations in the multiple-walker scheme^84^ using 32 walkers. To perform free energy calculations, we imposed upper and lower walls on the z-component of CVs—diffusion direction (the same direction as the membrane surface normal and same di) as:

Upper wall1 = CV1 < 40.0 Å, with force constant=24000.0 kcal mol^-1^ Å^−2^

Lower wall1 = CV1 > 24.0 Å, with force constant=24000.0 kcal mol^-1^ Å^-2^

Upper wall2 = CV2 < 40.0 Å, with force constant=24000.0 kcal mol^-1^ Å^-2^

Lower wall2 = CV2 > 24.0 Å, with force constant=24000.0 kcal mol^-1^ Å^-2^

The metaMD bias was deposited with a Gaussian width of 1.0 Å, an initial Gaussian amplitude of 0.48 kcal/mol, a deposition period of 1.0 ps, and a bias factor of 20. We continued the metaMD simulation (aggregated ~3 μs) to attain convergence of the free energy profiles.

### Diffusion-mechanism

Starting from the optimized biligand conformations on the membrane surface, we gradually pulled each biligand to diffuse through the membrane to reach the other membrane surface. We extracted 32 different conformations of biligand throughout the lipid membrane and subjected each to 40 ns simulation (aggregated ~2.5 μs) to relax the simulation box. These 32 different conformations were fed into our subsequent multiple-walker metaMD free energy calculations to estimate the energy barrier of diffusion. The metaMD bias forces were imposed on the CVs (defined above). To expedite the sampling process, we imposed upper and lower walls on our chosen CVs as:

Upper wall1 = CV1 < 25.0 Å, with force constant=239.0 kcal mol-1 Å^-2^

Lower wall1 = CV1 > −25.0 Å, with force constant=239.0 kcal mol-1 Å^-2^

Upper wall2 = CV2 < 30.0 Å, with force constant=239.0 kcal mol-1 Å^-2^

Lower wall2 = CV2 > −25.0 Å, with force constant=239.0 kcal mol-1 Å^-2^

The metaMD bias was deposited with a Gaussian width of 1.0 Å, an initial Gaussian amplitude of 0.48 kcal/mol, a deposition period of 1.0 ps, and a bias factor of 25. We continued the metaMD simulation (aggregated ~37 μs) to attain convergence of the free energy profiles.

### Reporting summary

Further information on research design is available in the Nature Portfolio Reporting Summary linked to this article.

## Supplementary information

Supplementary Information

Reporting Summary

## Data Availability

All data generated or analyzed during this study are included in this published article (and its supplementary information files)
