# Supplementary material for: Stereochemical engineering yields a multifunctional peptide macrocycle inhibitor of Akt2 by fine-tuning macrocycle-cell membrane interactions
Source: Commun Chem. 2023 May 18;6:95. doi: 10.1038/s42004-023-00890-w (PMC10195864; doi:10.1038/s42004-023-00890-w)

SUPPLEMENTARY INFORMATION

Stereochemical engineering yields a multifunctional peptide macrocycle inhibitor of Akt2 by fine-tuning macrocycle-cell membrane interactions

Arundhati Nag[†], Amirhossein Mafi[††], Samir Das, Mary Beth Yu, Belen Alvarez-Villalonga, Soo-Kyung Kim[††], Yapeng Su[†††], William A. Goddard III[††], and James R. Heath[†††]*

California Institute of Technology, Pasadena, California 91125

TABLE OF CONTENT:

Materials: 4
Solid phase peptide synthesis: 4
HTRF assay: 5
GSK3 inhibition assay: 6
Phospho-antibody competition assay: 6
Scheme S1: Epitope targeting screen against target phosphopeptide Akt2 450-481 with pS474 using CuAAC cyclized OBOC peptide library yields four hit candidates. 8
Table S1: Detailed protocol of chemical epitope screen 9
Table S2: Sequence of hits from chemical epitope screen. 10
Figure S1: Single point ELISA for peptide candidates obtained from chemical epitope screen and structure of best ligand 11
Figure S2: Binding assay for linear and cyclic versions of C_1 to target phospho-peptide demonstrate positive effect of cyclization on binding. 12
Figure S3: Decreasing ring size of C_1 reduces binding to target chemical epitope. 13
Figure S4: Variation of chemical nature of ring closing functionalities and ring size of cyclic hit C_1 causes variation of its Akt2 binding properties. 14
Figure S5: Structure and affinity (EC_{50}) of best macrocyclic ligand C_2 as determined by ELISA against full length pAkt2 protein. 15
Figure S6: Comparison of selectivity of C_2 and C_1 to pAkt isoforms. 16
Figure S7: Molecular Structure of C_2-N_1 used in the in situ click screen. 17
Scheme S2: Screen of full length phosphorylated Akt2 protein for biligand development. 18
Table S3: Detailed protocol of biligand screen as shown in Scheme S2. 19
Table S4: Sequence of linear component hits from biligand screen 20
Figure S8: Structure of biligands corresponding to hits $B_{2,2,1}$ (A) and hits B, C, D from screen 21
Figure S9: Biligand $B_{2,2,1}$ is selected by comparing ELISA binding assays of biligands to pAkt2 protein in buffer and in 1% serum. 23
Figure S10: Binding affinity of biligand $B_{2,2,1}$ to pAkt2 protein determined by sandwich ELISA assay. 24
Biotinylated biligand $B_{2,2,1}$ was immobilized on 24
Figure S11: Biligand $B_{2,2,1}$ binds to p-S474 site on pAkt2. 25
Figure S12: Biligand $B_{2,2,1}$ binds to pAkt2, almost similarly to pAkt1 and minimally to pAkt3 and inactive Akt2. 26
Biotinylated biligand $B_{2,2,1}$ was immobilized on 26
Figure S13: Inhibition of Akt kinase activity by biligand $B_{3,2,1}$. 27
Figure S14: Alanine scan of cyclic monoligand C_2. 28
Figure S15: Effect of binding to Akt2 protein for 4-fluorophenylalanine substituted derivatives of C_2 (Cy(YYTYTG-rcm)) 29
Figure S16: N-methylation of C_2 negates binding of the ligands to pAkt2. 30
Figure S17: Replacement of linker Nj by polyalanine linker and α,α-disubstituted α-amino acid. 31
Figure S18: Molecular structures of biligands in which Nj is replaced by PEG linker or arginine is incorporated into Nj or rigid aromatic residues are incorporated in Nj. 32
Figure S19: Molecular structures of N-methylated biligands $B_{3,2,2}$ – $B_{3,2,5}$. 33
Figure S20: Biligands $B_{3,2,2}$ – $B_{3,2,5}$ which have N-methylated amino acids incorporated in the linear component, show binding to pAkt2. 34
Figure S21: Molecular structure of biligand $B_{4,2,5}$. 35
Figure S22: Biligand $B_{4,2,5}$ demonstrates good binding to pAkt2 protein (EC_{50} 60.6 nM) 36
Figure S23: MD simulations of $B_{4,2,5}$. 37
Figure S24: Understanding retro-inversion of the cyclic component C_5 to C_6 38
Figure S25: Molecular structures of retro-inverted biligands $B_{5,3,4}$, $B_{5,3,5}$, $B_{6,3,4}$ and $B_{6,3,5}$. 39

Figure S26: Retro-inverted biligands $B_{6,3,4}$ and $B_{6,3,5}$ demonstrate excellent binding characteristics (EC_{50} 10.8 and 7.05 nM) for pAkt2. 40
Figure S27: Biligand $B_{7,3,5}$, containing D-(2R,3R) threonines and biligand $B_{5,3,5}$, containing D-(2S,3R) threonines, have similar affinities (EC_{50} 11.80 nM and 15.06 nM respectively) for pAkt2 protein. 41
Figure S28: Pulldown using retro-inverted ligands $B_{5,3,5}$ and $B_{7,3,5}$ as immunoprecipitation reagents. 42
Figure S29: Fluorescence microscopy of non-stimulated OVCAR3 cells with fluorescein labeled $B_{7,3,5}$ 43
Figure S30: Solution-diffusion mechanism of the biligands, $B_{5,3,5}$ and $B_{7,3,5}$, cell penetrating characteristics. 44
Figure S31: The analysis of non-bonded interactions between the macrocycle of the biligands ($B_{5,3,5}$ and $B_{7,3,5}$) and the components of the lipid membrane (POPC and cholesterol). 45
Figure S32: Flow cytometry gating strategy of NIH OVCAR3 cells treated with FITC-$B_{7,3,5}$. 46
Mass spectroscopic characterization of peptides: 47

Materials:
Fmoc amino acids were purchased from Anaspec and AAPPTec and used as received. TentaGel S-NH_2 resin (diameter 90 µm, capacity 0.28 mmol/g) was obtained from Anaspec and utilized for OBOC library construction. Biotin NovaTag™ resin, Biotin – PEG NovaTag™ resin, Fmoc – NH - $(PEG)_2$ - OH (13 atoms) were obtained from EMD Chemicals, Inc. and used for synthesis of biotinylated peptides. Amide Sieber resin (capacity 0.3-0.6 mmol/g) purchased from Anaspec was used for synthesis of protected peptides. NMP (1-methyl-2 -pyrrolidinone), HATU ((2-(7-Aza-1H-benzotriazole-1-yl)-1,1,3,3-tetramethylammonium hexafluorophosphate) and DIEA (N,N′-diisopropylethylamine) used in peptide synthesis were bought from EMD Chemicals, Inc., ChemPep and Sigma-Aldrich respectively. DMF (N,N′-dimethylformamide), piperidine, TFA (trifluoroacetic acid, 98% min. titration), and TES (triethylsilane) were purchased from Sigma-Aldrich. 5-Azido-pentanoic acid was purchased from Bachem Americas, Inc. BCIP (5-Bromo-4-chloro-3-indolyl phosphate) was purchased from Promega.

Active Akt2 (with N terminal His_6 tag) was purchased from Abcam. Inactive Akt2 (with N terminal His_6 tag) was purchased from BPS Bioscience. Active Akt1 and Akt3 (with N terminal His_6 tag) used in ELISA assays were purchased from Sigma Aldrich. Mouse anti biotin antibody-Alkaline Phosphatase conjugate used in screens was purchased from Sigma Aldrich. Anti His_6 mouse antibody, goat anti mouse IgG - Alkaline Phosphatase conjugate used in screens were purchased from Abcam. Anti Akt (pan) rabbit antibody (11E7), PhosphoAkt (S473) antibody, pGSK3 antibody, beta-actin mouse antibody and mouse anti-rabbit antibody (HRP conjugate) used in Western blot were purchased from Cell Signaling Technology. Fluorescent dye labeled antibodies used for western blot were Alexa 790 conjugated goat anti-rabbit antibody (Abcam, ab186697) and Alexa 700 conjugated goat anti-mouse antibody (Invitrogen, A21036).

Solid phase peptide synthesis:

Acylation: The resin was treated with a solution of anhydrous acetic anhydride and 2,6- lutidine (Sigma) in DMF (acetic anhydride: 2,6-lutidine: DMF; 5:6:100), twice for ten minutes at room temperature. The excess reagents were removed by five washes with DMF.

Cleavage of side chain protected peptides: The peptides were synthesized on Sieber Amide resin. The resin was treated three times for one minute with 1% TFA/DCM and then washed with DCM. The peptide solution was neutralized by adding 2 equivalent DIEA and removed under vaccuam trap. The semisolid was dissolved in filtered DMSO, HPLC grade acetonitrile and double distilled water and purified on the HPLC.

Cleavage of side chain deprotected peptide: The peptides were synthesized on the Rink Amide MBHA, Biotin Novatag or Biotin PEG Novatag resin. The resin was treated with a TFA cleavage solution

(TFA: TES: ddH_2O; 95:2.5:2.5) for two hours at room temperature. The cleavage solution was filtered through a Gooch filter crucible and added dropwise to an ice cooled solution of diethyl ether.

Protocol for on bead copper (Cu) catalyzed azide alkyne cycloaddition (CuAAC) click reaction:

On bead Cu catalyzed click reactions were performed with the azide on bead and the alkyne in solution. The resin was treated with 2 equivalents of the relevant alkyne, 1.5 equivalents of CuI (Sigma) and 2.5 equivalents of ascorbic acid (Sigma), in a solution of 20% piperidine in DMF. The reaction was performed overnight at room temperature. The excess copper was removed from the resin by washing extensively with a Cu chelating solution (5% (w/v) sodium diethyl dithiocarbamate, 5% (v/v) DIEA in DMF).

Comprehensive CuAAC cyclized peptide library Synthesis:

The structure and details of the library is shown below:

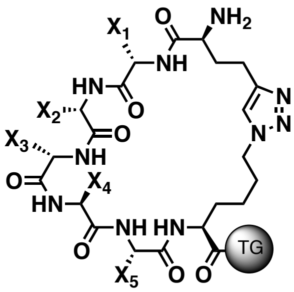

Xi =L-Ala, Gly, L-Leu, L-Ile, L-Val, L-Phe, L-Trp, L-Arg, L-His, L-Lys, L-Asp, L-Glu, L-Asn, L-Gln, L-Ser, L-Thr, L-Tyr, L-Pro.

Diversity: 1889568

Synthesis of scrambled phospho-epitope and target phospho-epitope:

Akt2 450-481 (ITPPDRYDSLGLLELDQRTHFPQF(pS)YSASIRE) is used as the target phospho-epitope. The scrambled non-phosphorylated epitope has sequence ITPPDRYDSLGLLELQRTH-YFFASQPSS-IRE, in which the amino acids 450-468 and 479-481 are kept unchanged and the hydrophobic motif (HM) amino acids 470-478 is scrambled. For screening both peptides are synthesized on Rink Amide MBHA resin with a polyethylene glycol spacer (PEG_2) and biotin on the N terminal.

HTRF assay:

HTRF assay for inhibition of pAkt2 was done following the manufacturer protocol (cisbio assays) for HTRF KINease-STK kit. Briefly, kinase, STK peptide substrate, ligand inhibitor and ATP solutions were made per the given protocol so that the final concentration in the assay was 12.5 nM Akt2, 125 uM ATP, 1.25 uM

STK substrate and the biligand series dilution concentration was done from 20 uM. Following incubation at room temperature for half hour, SaXL665 and STK Ab-cryptate was added, incubated for 1 hour at room temperature, and fluorescence was measured at 620 nm (cryptate) and 665 nM (XL665). A A665/A620 ratio was calculated for each well.

GSK3 inhibition assay:

The non–radioactive kinase assay kit for Akt2 was purchased from Cell Signaling. Ligand solutions were made in DMSO. Kinase reactions were set up in 1X kinase buffer (25 mM trisHCl (pH 7.5), 10 mM MgCl2, 0.01% Triton-X, 1X Complete protease inhibitor (Roche), 1X Phosstop phosphatase inhibitor (Roche)), each 25 μL reaction mixture containing 400 ng Akt2, 400 ng GST-GSK-3α/β fusion protein, 500 μM ATP and 2 μL of peptide solutions in DMSO or DMSO only. The peptide concentration series was made from 50 uM to 0.08 μM. Reactions were allowed to proceed for 30 minutes at 30°C. The reactions were quenched by adding12.5 μL of 2X SDS sample loading buffer and heating at 95° C for five minutes. 10 μL from each sample was loaded on an any kD SDS gel (Biorad) and run for an hour at 110 volts. Following semidry transfer of the gel to a nitrocellulose membrane, the membrane was blocked in 5% nonfat milk/TBST for an hour at 4°C and treated overnight at 4°C with a 1:200 dilution of Phospho GSK-3α/β Ser (21/9) rabbit antibody (Cell Signaling) in 0.5% milk/TBST. Following washes, the membrane was treated with a 1:2000 dilution of mouse anti-rabbit –HRP antibody for an hour at 4°C. After four five-minute washes with TBST, and one five-minute wash with TBS, the membrane was treated with West Dura ECL substrate (Thermo Scientific) and imaged on film.

Phospho-antibody competition assay:

2 μM biotinylated ligand or control Ac-gly-biotin was prepared by diluting the 1 mM stock in binding buffer (0.1% BSA, 25 mM trisCl, pH = 7.5, 150 mM NaCl, 0.05% Tween20) and added to High-Capacity Streptavidin 96 well plate (Thermo Scientific). After washing away the excess ligand, the plate was blocked for 3 hours with 2% BSA in TBST and washed 3 times with binding buffer. A series of solutions were made with 25 nM His_6-Akt2 and serial dilutions of the pS473 antibody (Cell Signaling) from 1/10,00 to 1/20,000 of the antibody stock and the solutions shaken for 1 hour at 4°C. After three washes with the binding buffer, the plate was treated for an hour with a 1:1000 dilution of anti-His6 mouse monoclonal antibody in binding buffer at 4°C. A 1:10,000 dilution of goat anti-mouse antibody conjugated to Horse Radish Peroxide in binding buffer was added to the wells at 4°C for an hour. The plates were washed four times, five minutes each, with TBST and once with TBS. Color was developed by adding TMB substrate to each well. The reaction was quenched with 0.5 M H_2SO_4. The absorbance values at 450 nm (A450) were measured on a 96-well plate reader. The Net A450 was obtained by subtracting the corresponding absorbance values for

the blank control (ac-gly-biotin, treated with same solutions of Akt and antibody dilutions) from the observed absorbance values.

Scheme S1: Epitope targeting screen against target phosphopeptide Akt2 450-481 with pS474 using CuAAC cyclized OBOC peptide library yields four hit candidates.

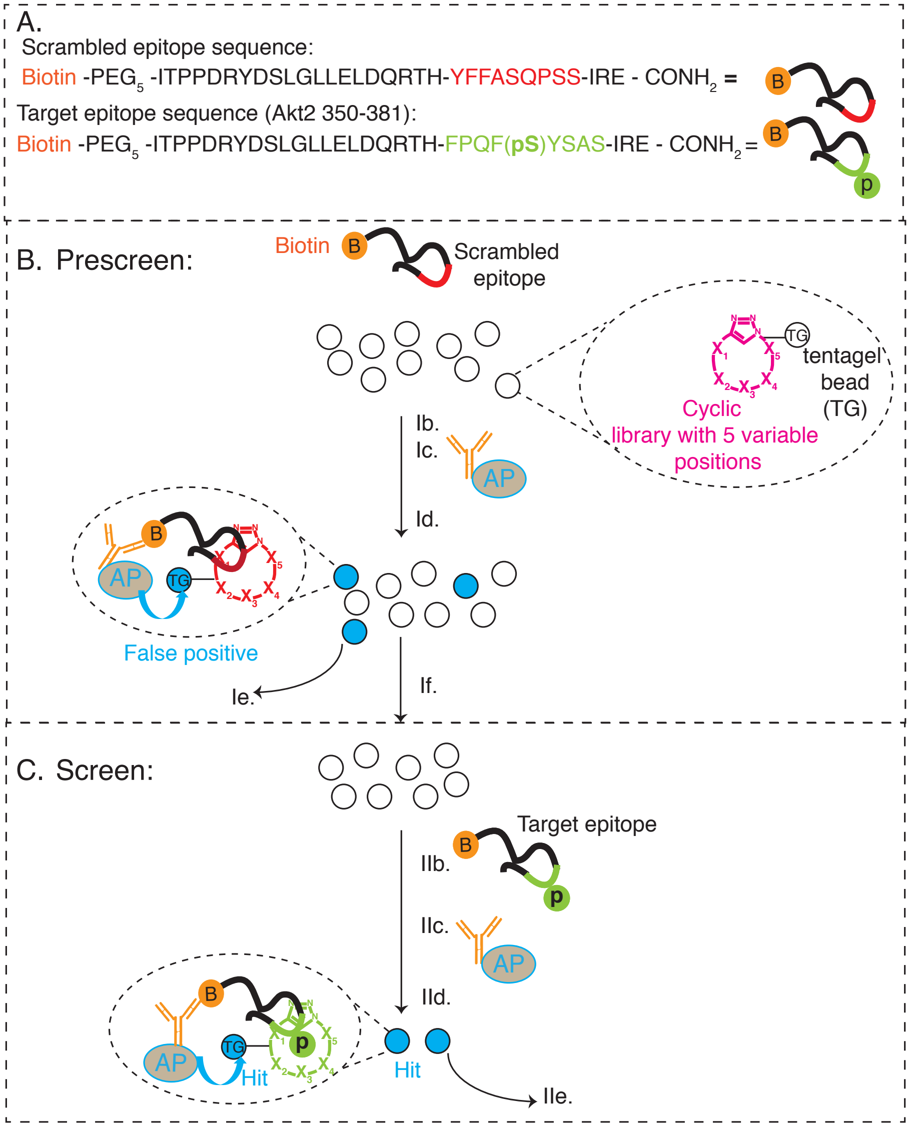

A. Sequences of the scrambled and target peptide epitopes. B. Prescreen: Approximately 1.145 million beads from a 'CuAAC' cyclized solid-phase heptamer library (denoted by triazole ring) on TentaGel (labeled TG) is screened against a His6 and biotin (orange circle labeled B) tagged non-phosphorylated peptide corresponding to Akt2 450-481 (black and red). The peptide is scrambled in the hydrophobic motif (HF) (red) containing S474. The library is probed with an anti- His6 antibody (orange) conjugated to alkaline phosphatase (tan circle labeled AP) and AP substrate BCIP. Hit beads (blue) are false positive binders to the antibody and scrambled peptide and are discarded. The remaining library is washed, stripped, and equilibrated in buffer; C. Screen: The His6 and biotin tagged target epitope (black and green) that contains the HF (green) with phospho-Ser474 is screened against the cleared library. Hit beads to the target epitope are determined by probing with an anti- His6 antibody conjugated to AP and AP substrate BCIP, which turn the beads turquoise. These are manually separated, washed, stripped of protein, and sequenced by the Edman Peptide Sequencer. The prescreen resulted in hit frequencies between 10% and 12% of the beads in the library while the screen resulted in 0.003 % hit rates for sequencing. Consult table S1 for details of screening protocols.

Table S1: Detailed protocol of chemical epitope screen

**Prescreen**	
Ia.	Incubate the cleared one bead one compound CuAAC cyclized peptide library with 500 nM biotinylated scrambled peptide (Biotin-PEG-ITPPDRYDSLGLLELQRTH-YFFASQPSS-IRE). Remove excess peptide by three five-minute washes with the binding buffer.
Ib. Ic.	Treat with 1:10,000 dilution of anti-biotin antibody conjugated with alkaline phosphate (AP). (Sigma, A6561). Remove excess antibody by three five-minute washes with binding buffer. Wash library three times for five minutes with wash buffer 1; three times for five minutes with wash buffer 2 and three times for five minutes with AP buffer.
Id.	Treat with BCIP, when the hit beads turn blue.
Ie.	Manually pick up blue hit beads. Wash remaining library with 7.5M guanidinium hydrochloride (pH 2) for two hours, then with DMF overnight, and then with double distilled water ten times for three minutes each. Equilibrate cleared beads by incubating with binding buffer overnight.
**Screen**	
IIa.	Incubate the cleared one bead one compound CuAAC cyclized peptide library with 500 nM biotinylated phosphorylated peptide that corresponding to Akt2 450-481 (Biotin-PEG-ITPPDRYDSLGLLELQRTH- FPQF(pS)YSAS -IRE), where pS corresponds to phosphoSer474.
IIb.	Remove excess peptide by three five-minute washes with the screening buffer.
IIc.	Treat with 1:10000 dilution of anti-biotin antibody conjugated with alkaline phosphate (AP). (Sigma, A6561). Remove excess antibody by three five-minute washes with binding buffer. Wash library three times for five minutes with wash buffer 1; three times for five minutes with wash buffer 2 and three times for five minutes with AP buffer.
IId.	Treat with BCIP, when the hit beads turn blue.
IIe.	Manually pick up blue hit beads. Wash the hit beads with 7.5M guanidinium hydrochloride (pH 2) for two hours, then with DMF overnight, and then with double distilled water ten times for three minutes each. Load each bead in one cartridge of the Edman Peptide Sequencer for sequencing.

Table S2: Sequence of hits from chemical epitope screen.

Cyclic Peptide Library: Cy (X1X2X3X4X5 –Tz4) -TG
D-Pra = D-Propargylglycine
Xi = All 16 D amino acids except D-Met, D-Cys
TG = tentagel S-NH2 bead

Tz4 =

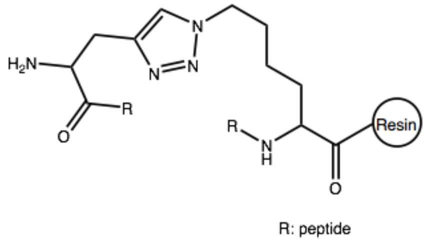

Name	Cyclic	(X_1	X_2	X_3	X_4	X_5	Tz4)
	Cy	(Y	V	Y	K	S	Tz4)
	Cy	(V	F	A	K	V	Tz4)
	Cy	(I	R	Y	Y	S	Tz4)
**C_1**	Cy	(Y	Y	T	Y	T	Tz4)

Figure S1: Single point ELISA for peptide candidates obtained from chemical epitope screen and structure of best ligand

Following the ELISA protocols detailed, 1.25 μM of the biotin conjugated cyclic peptide hits were immobilized onto Streptavidin ELISA plates. A PEG_5-biotin was used as the no-ligand blank. The wells are treated with the 2.5 μM solutions of target epitope His_6–Peg_2-ITPPDRYDSLGLLELDQRTH-FPQF(pS)YSAS-IRE and the scrambled epitope His_6–Peg_2-ITPPDRYDSLGLLELQRTH-YFFASQPSS-IRE overnight. The wells are treated consecutively with mouse His_6 antibody, anti-mouse IgG conjugated to horse radish peroxidase (HRP) and HRP substrate TMB. The absorbance at 450 nm is measured to quantitate the binding. The results demonstrate that $\mathbf{C_1}$, with sequence Cy(YYTYT-Tz4), has better binding affinity for target chemical epitope than other candidate; B. Molecular structure of biotin-tagged $\mathbf{C_1}$ used in assays.

A.

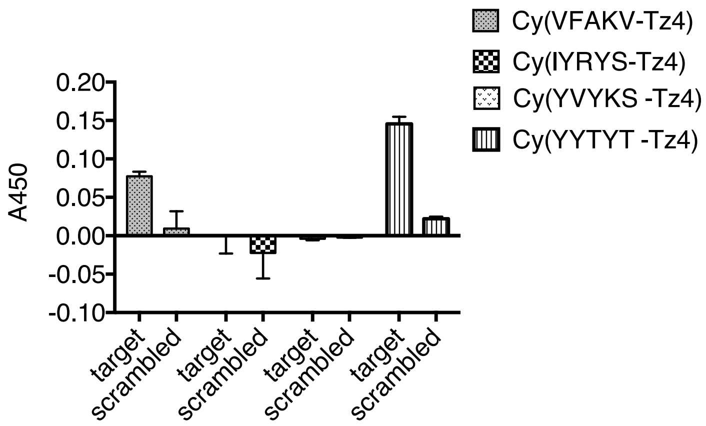

B.

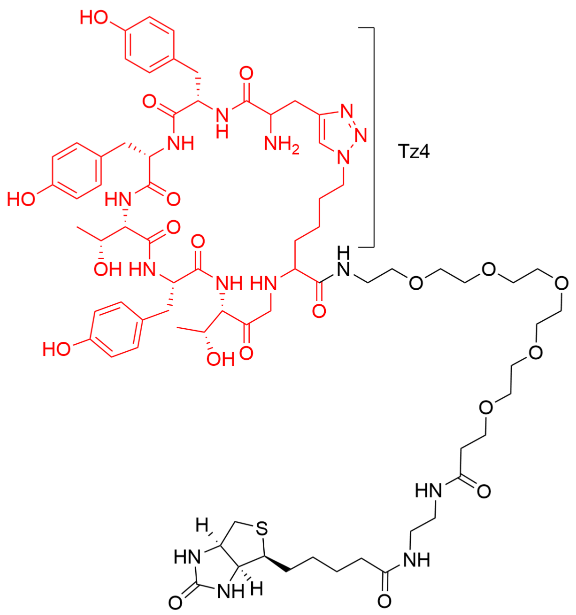

Figure S2: Binding assay for linear and cyclic versions of C_1 to target phospho-peptide demonstrate positive effect of cyclization on binding.

1.25 μM biotin conjugated cyclic peptide $\mathbf{C_1}$, with sequence Cy(YYTYT-Tz4), and corresponding linear peptide L-Pra-YYTYT-L-Az4 are immobilized on a streptavidin plate and treated with 2.5 μM target epitope His6–Peg2-ITPPDRYDSLGLLELDQRTH-FPQF(pS)YSAS-IRE. Binding is quantified by subsequent treatments with mouse His_6 antibody, mouse IgG–HRP antibody and HRP substrate. The cyclized sequence $\mathbf{C_1}$, binds significantly better than the linear non-cyclized version L-Pra-YYTYT-L-Az4 to the target phosphorylated chemical epitope.

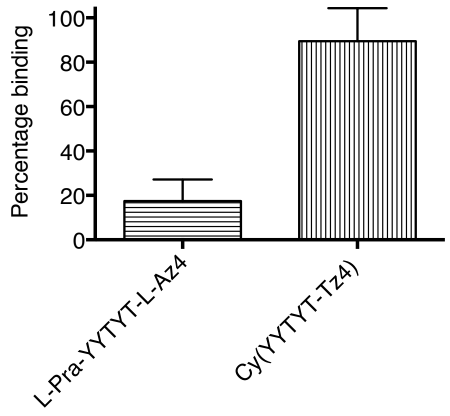

Figure S3: Decreasing ring size of C_1 reduces binding to target chemical epitope.

Efforts were made to synthesize variations of **C_1** with a smaller ring size by making the triazole functionality by CuAAC reaction between azidoalanine and propargylglycine (Tz1) and by CuAAC reaction between azidohomoalanine and propargylglycine (Tz2). The cyclic peptide (YYTYT-Tz1) could not be isolated from the synthesis. A. Molecular structure of Cy(YYTYTTz2) used in assay; B. 1.25 μM biotinylated cyclic peptides **C_1** and Cy(YYTYT-Tz2) are immobilized on plate and treated with 2.5 μM His_6 tagged target phosphorylated chemical epitope. Following antibody treatment and enzyme treatment for signal readout, absorbance at 450 nm is measured to quantitate binding. It is observed that C_1, with sequence Cy(YYTYT-Tz4) i.e. cyclized with a chain longer than Cy(YYTYT-Tz2) by two methylene units, is a better binder than the latter for the target phosphorylated chemical epitope.

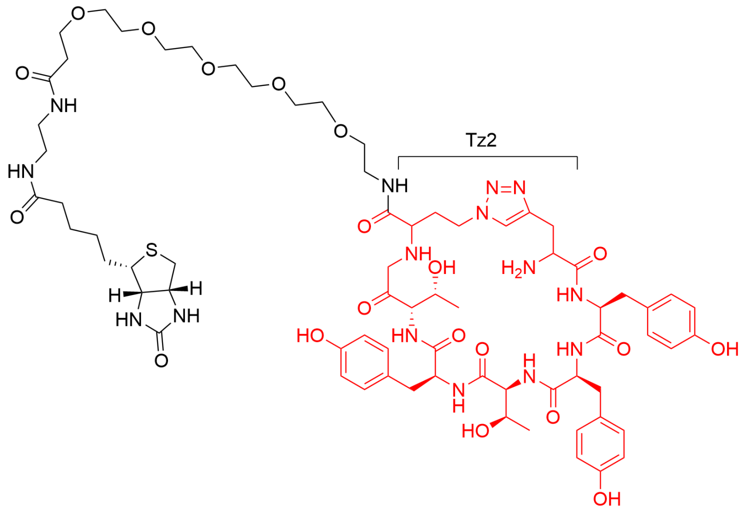

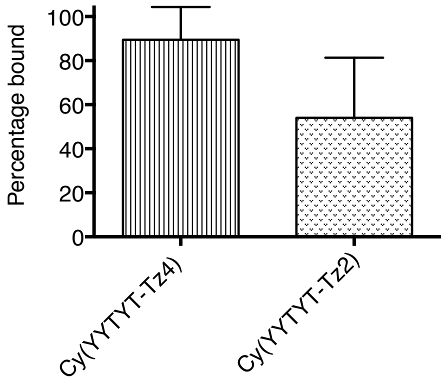

Figure S4: Variation of chemical nature of ring closing functionalities and ring size of cyclic hit C_1 causes variation of its Akt2 binding properties.

Biotin tagged ligand $\mathbf{C_1}$, sequence Cy(YYTYT-Tz4), click cyclized ligand Cy(YYTYTG-Tz4) and RCM cyclized ligand $\mathbf{C_2}$, sequence Cy(YYTYTG-rcm) are immobilized on Streptavidin plate, and treated with 25 nM full length pAkt2 protein. The binding is detected by antibody treatment, as described in the ELISA protocol, and measured by absorbance at 450 nm. While the ligand $\mathbf{C_1}$ binds to the target pAkt2 protein, the click cyclized ligand Cy(YYTYTG-Tz4) with an extra glycine does not, while the RCM cyclized ligand $\mathbf{C_2}$ with sequence Cy(YYTYTG*rcm*) binds with the highest signal.

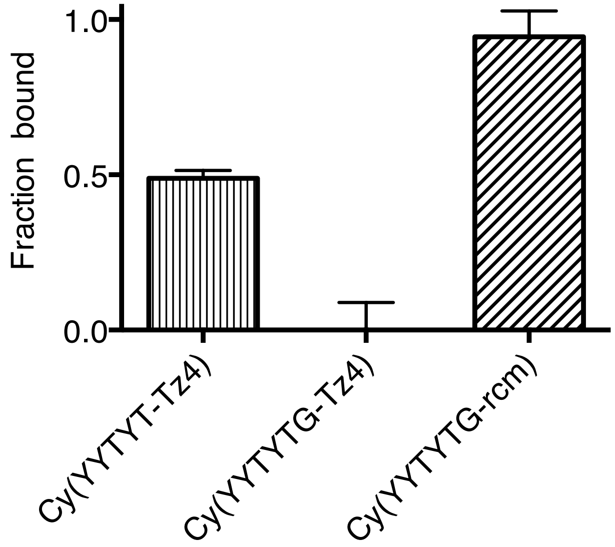

Figure S5: Structure and affinity (EC_{50}) of best macrocyclic ligand C_2 as determined by ELISA against full length pAkt2 protein.

Molecular structure of $\mathbf{C_2}$ B. Biotin conjugated $\mathbf{C_2}$ was immobilized on a streptavidin plate and treated with a serial dilution of His_6-pAkt2 overnight at 4°C. The wells were treated with anti 6X His mouse monoclonal antibody, goat anti-mouse antibody-Horse Radish Peroxide conjugate, TMB substrate and quenched, and the absorbance at 450 nm wavelength measured. From the absorbance values at 450 nm wavelength (A450) the corresponding blank A450 (Ac-gly-biotin immobilized, same concentration series of protein added) was subtracted to obtain the corrected absorbance values.

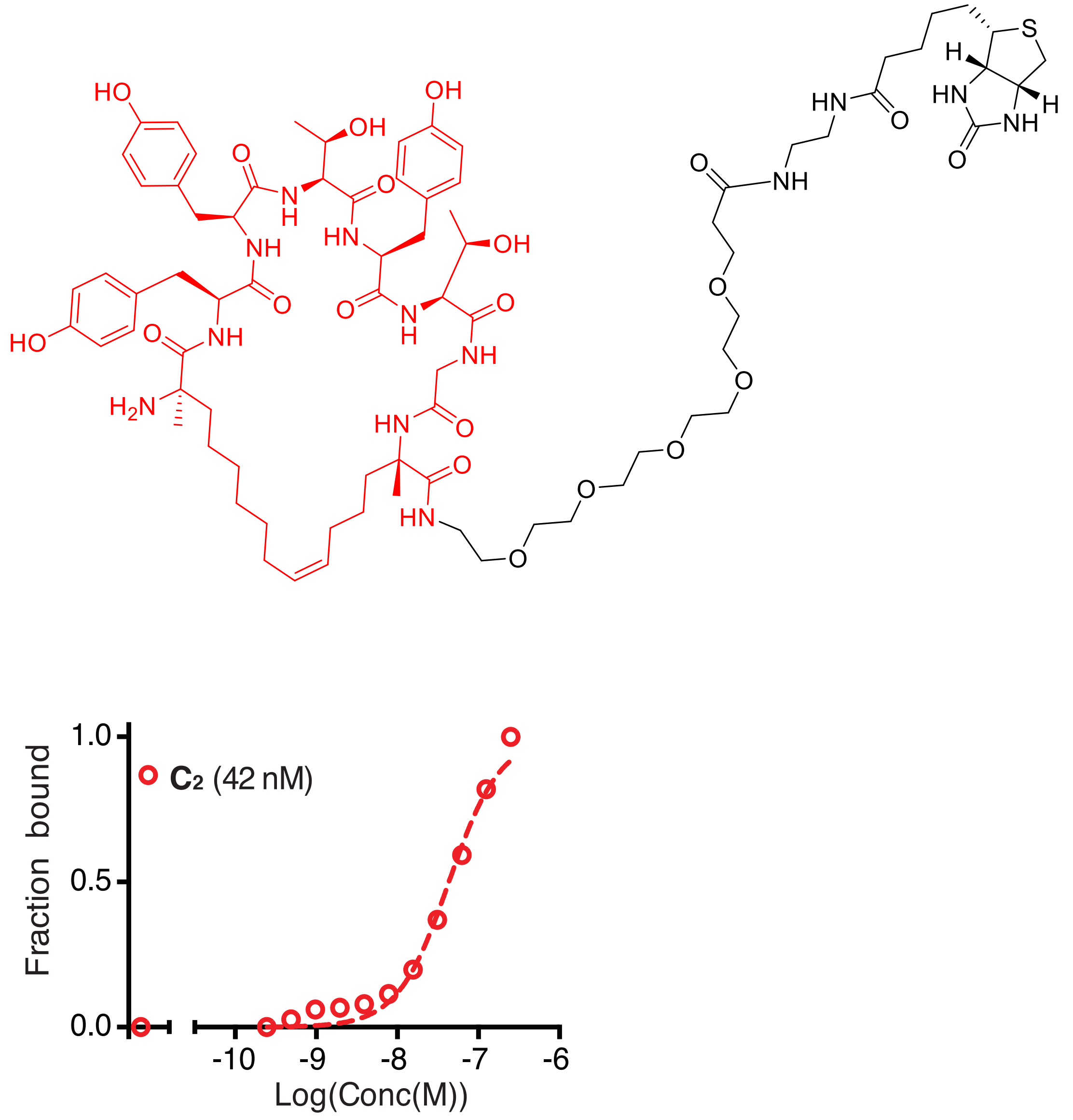

Figure S6: Comparison of selectivity of C_2 and C_1 to pAkt isoforms.

1.25 μM biotin conjugated cyclic peptides $\mathbf{C_1}$ and $\mathbf{C_2}$ are immobilized on streptavidin plates and treated with 25 nM His_6 -pAkt1, His_6 -pAkt2 and His_6 -pAkt3 protein respectively. Binding is quantified by subsequent treatments with mouse His_6 antibody, mouse IgG conjugate with HRP and HRP substrate and measuring absorbance at 450 nm wavelength. The y-axis scale is normalized, so that the strongest absorber is at 100% (with error bars). While both $\mathbf{C_1}$ and $\mathbf{C_2}$ are selective for pAkt2 over the other two isoforms pAkt1 and pAkt2, $\mathbf{C_2}$ shows higher selectivity.

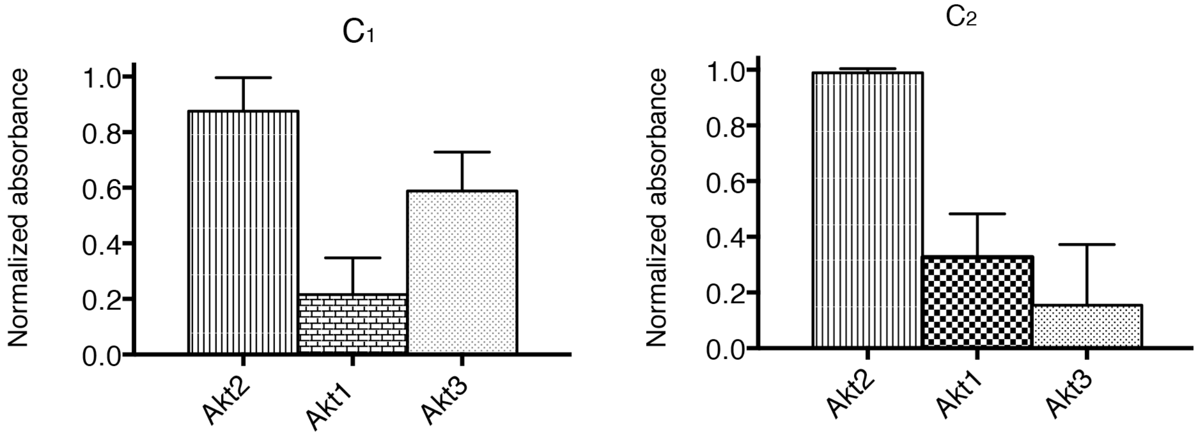

Figure S7: Molecular Structure of C_2-N_1 used in the *in situ* click screen.

In molecule **C_2-N_1**, an azide is appended to the C terminal of the cyclic ligand **C_2** though a 13-atom long chain and a biotin tag is also appended which is separated from the cyclic peptide and the azide by a long polyethylene glycol chain (PEG_5). Both the azide and biotin tags play essential roles in the *in situ* click screen.

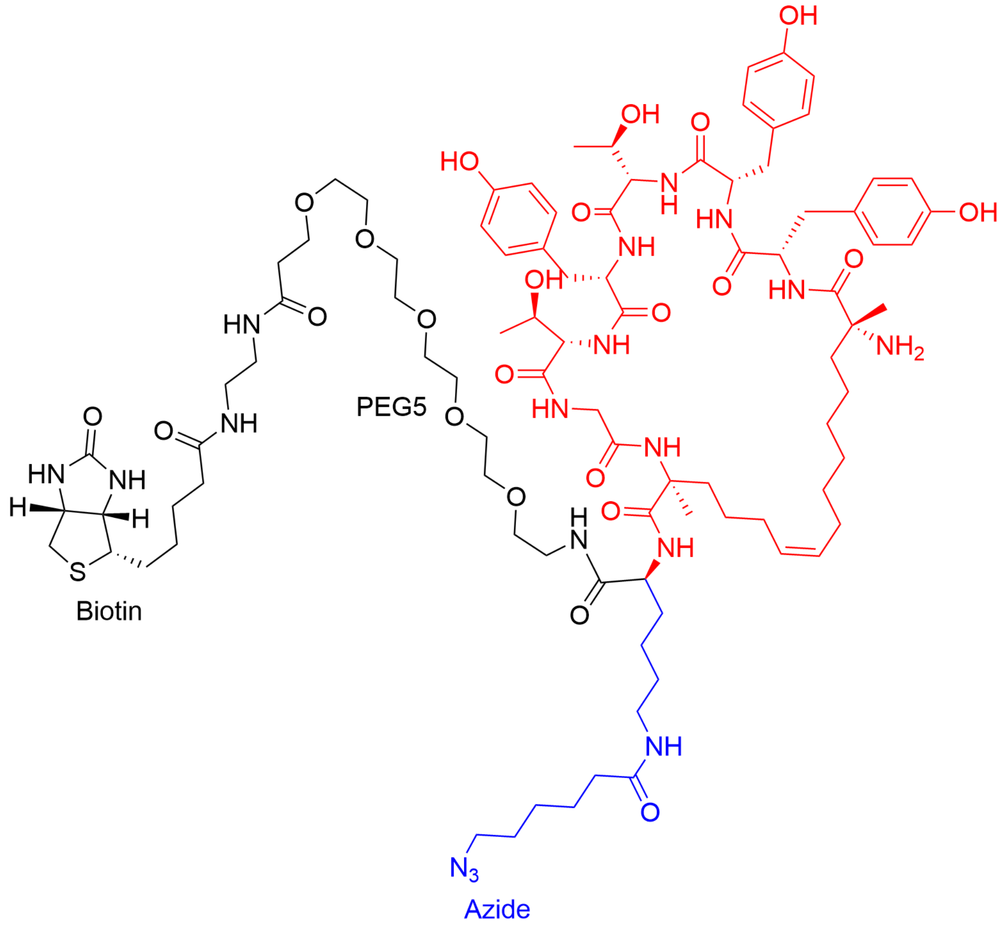

Scheme S2: Screen of full length phosphorylated Akt2 protein for biligand development.

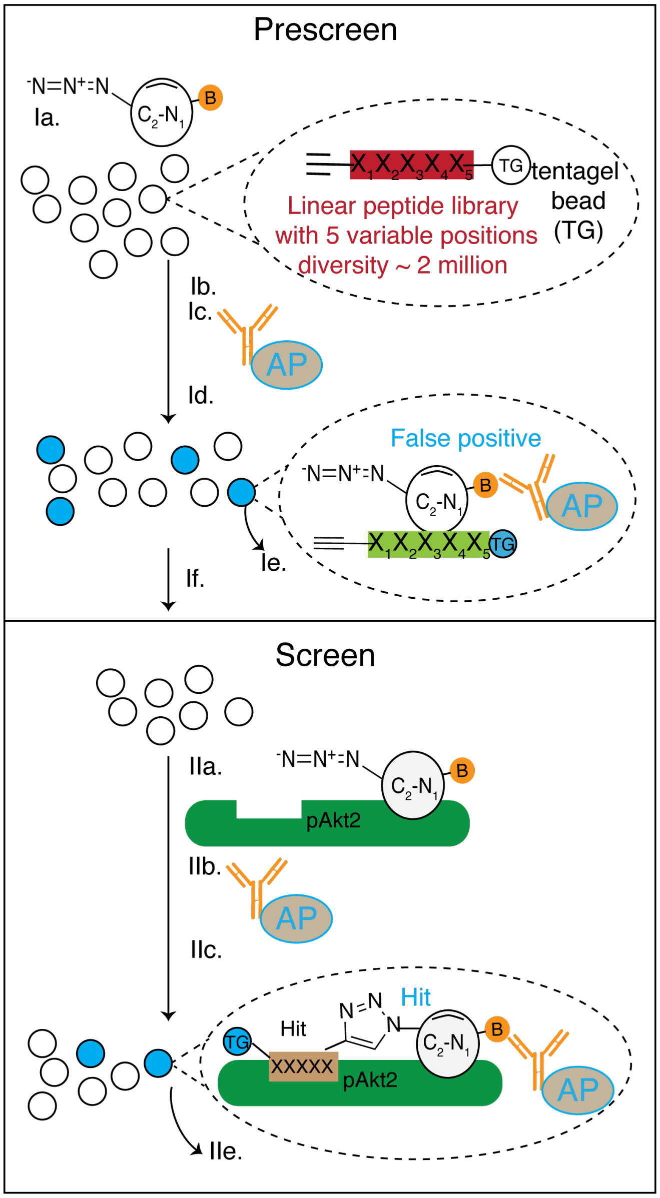

A. Prescreen: Biotinylated (orange circle labeled B) anchor **C₂-N₁** (black) is screened against approximately 1.18 million peptide beads (colored rectangles) from a comprehensive solid-phase hexamer library with an appended azide (black). The library is stripped of excess peptide and antibody and probed with an anti-biotin antibody (orange) conjugated to alkaline phosphatase (tan circle labeled AP) and AP substrate BCIP. False positive binders (blue circle) to **C_2-N_1** turn turquoise and are discarded. The cleared library is stripped of the antibody, kept in buffer overnight and screened against full length Akt2 (green) preincubated with anchor **C_2-N_1**. The library is then stripped of protein and hit beads which that bond to **C_2-N_1**, promoted by the scaffolding action of pAkt2, are detected by treating with an anti- biotin antibody (orange) conjugated to alkaline phosphatase (tan circle labeled AP). The hit beads are thoroughly washed and sequenced by the Edman Peptide Sequencer. The biligand prescreen resulted in hit frequencies between 5% and 10% of the beads in the library while the screen resulted in 0.003 % hit rates for sequencing.

Table S3: Detailed protocol of biligand screen as shown in Scheme S2.

	**Scheme S2 Prescreen**
Ia.	Incubate 500 mgs (approximately 1.145 million peptides) of a one bead one compound linear peptide library of sequence Propargylglycine-$X_1X_2X_3X_4X_5$ –TG (Xi: 18 amino acids except D-Met and D-Cys, TG: tentagel resin) with 500 nM biotinylated ligand C_1.
Ib.	Remove excess peptide with three five-minute washes with the screening buffer.
Ic.	Wash beads with 7.5 M guanidium hydrochloride (GuHCl), pH 2.0, for 2 hours, followed by five three-minute water washes with double distilled water (ddH2O), The beads are incubated with buffer for 8 hours.
Id.	Treat with 1:10,000 dilution of anti-biotin antibody conjugated with alkaline phosphate (mAb-AP) (Sigma A6561) for an hour at 4°C. then wash three times with screening buffer for five minutes each. Wash library three times for five minutes with wash buffer 1; three times for five minutes with wash buffer 2 and three times for five minutes with AP buffer.
Ie.	Treat with BCIP, when beads interacting with C_2-N_1 or mAb-AP turn blue
If.	Manually pick up and discard blue beads, which are false positives.
Ig.	After discarding blue beads, rest of library washed with DMF 2M GuHCl for two hours, then with DMF overnight. The cleared library is shaken for 8 hours in screening buffer.

	**Scheme S2 Screen**
IIa.	Incubate 50 nM pAkt2 with 25 μM ligand C2-N1 for 2 hours at 4°C. Screen this mixture against the cleared one bead one compound peptide library.
IIb.	Remove excess peptide with three five-minute washes with the screening buffer.
IIc.	Wash beads with 7.5 M GuHCl, pH 2.0, for 2 hours then with ddH2O for five times. The beads are incubated with the screening buffer for 8 hours.
IId.	Treat with /10,000 dilution of mouse anti-biotin antibody conjugated with alkaline phosphate (AP) (Sigma A6561) for an hour at 4°C. Remove excess antibody by three five-minute washes with screening buffer. Wash library three times for five minutes with wash buffer 1; three times for five minutes with wash buffer 2 and three times for five minutes with AP buffer.
IIe.	Treat with BCIP, when the hit beads turn blue.
IIf.	Manually pick up blue hit beads. The beads are washed with 2M GuHCl for two hours, followed by five three-minute water washes with ddH2O, kept in DMF overnight, and then washed ten times with double distilled water for three minutes each. Each bead is then loaded in one cartridge of the Edman Peptide Sequencer.

Table S4: Sequence of linear component hits from biligand screen

Linear Peptide Library: D-Pra-$X_1X_2X_3X_4X_5$ -TG
D-Pra = D-Propargylglycine
Xi = All 16 D amino acids except D-Met, D-Cys
TG = tentagel bead

Ligand name	D-Pra	X_1	X_2	X_3	X_4	X_5
L_1/A	D-Pra	K	y	y	I	r
B	D-Pra	K	y	y	F	k
C	D-Pra	Y	w	f	R	h
D	D-Pra	L	s	h	Y	r

Figure S8: Structure of biligands corresponding to hits $B_{2,2,1}$ (A) and hits B, C, D from screen

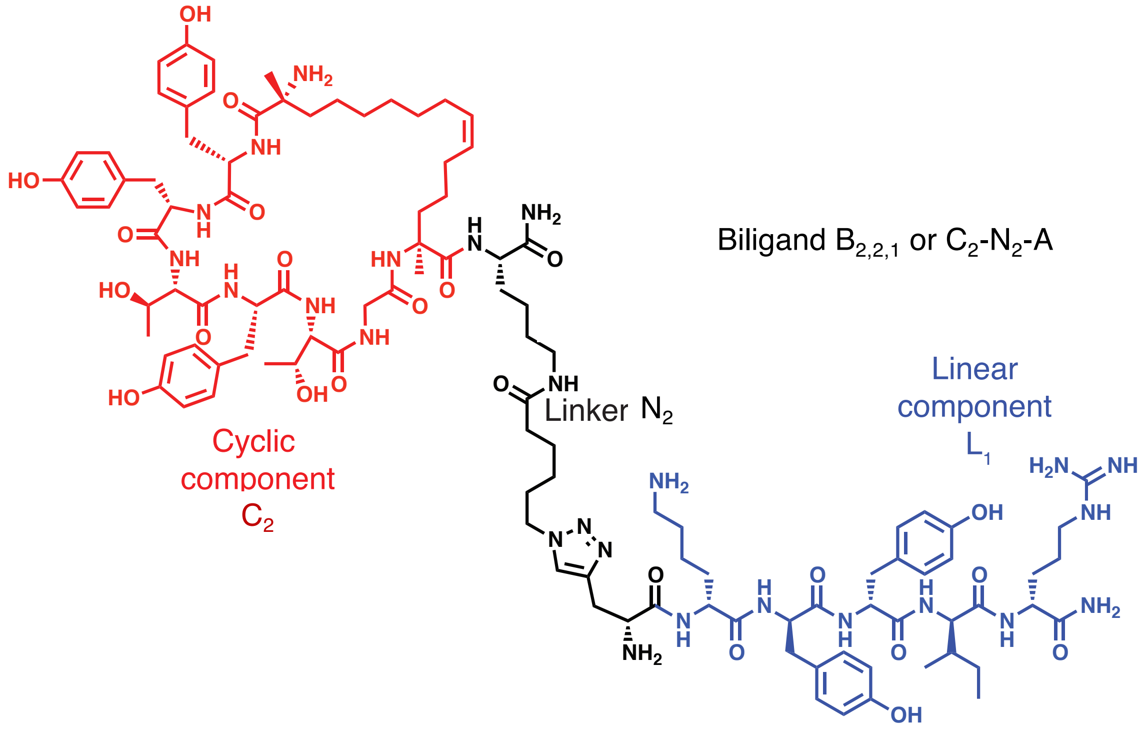

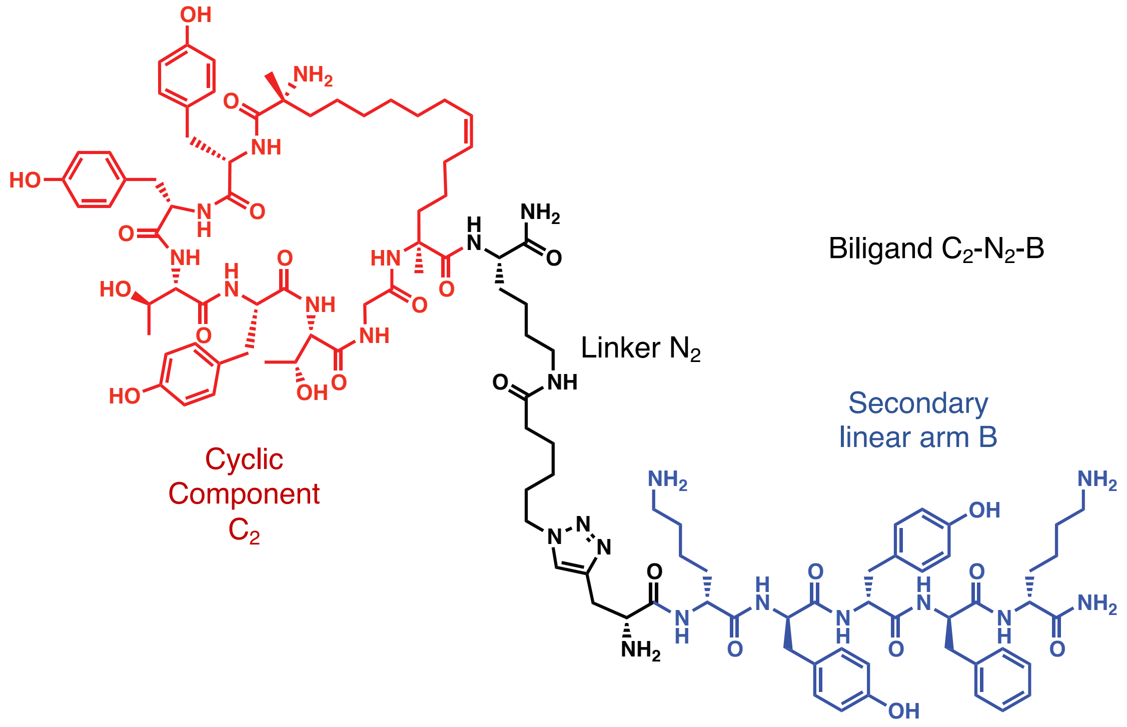

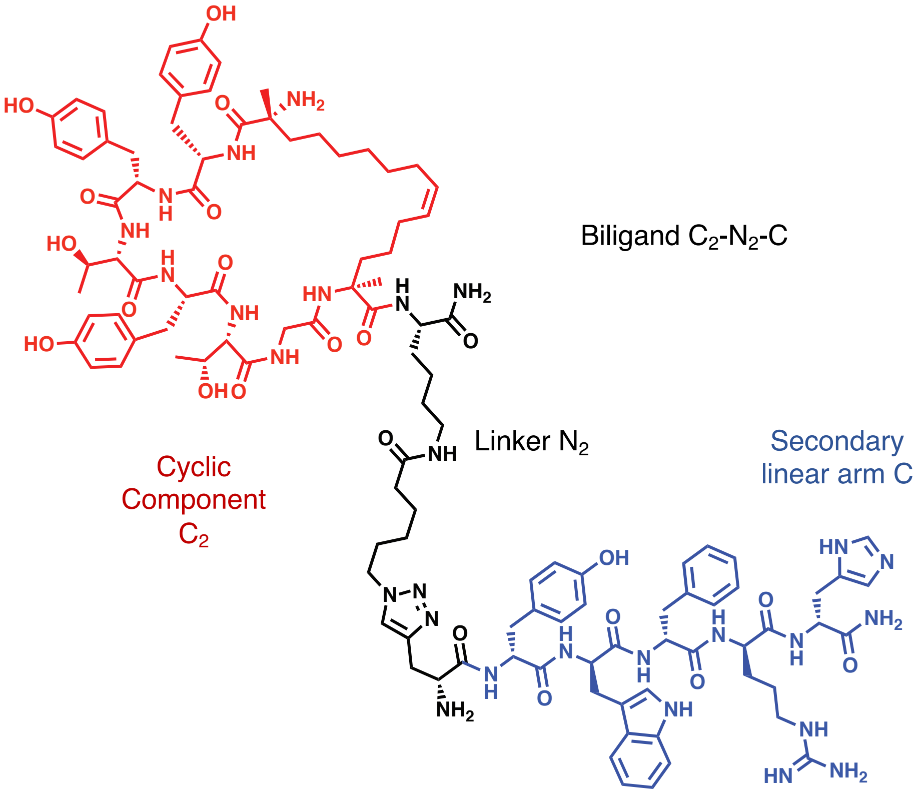
Biligand C2-N2-C
Linker N2
Cyclic Component C2
Secondary linear arm C

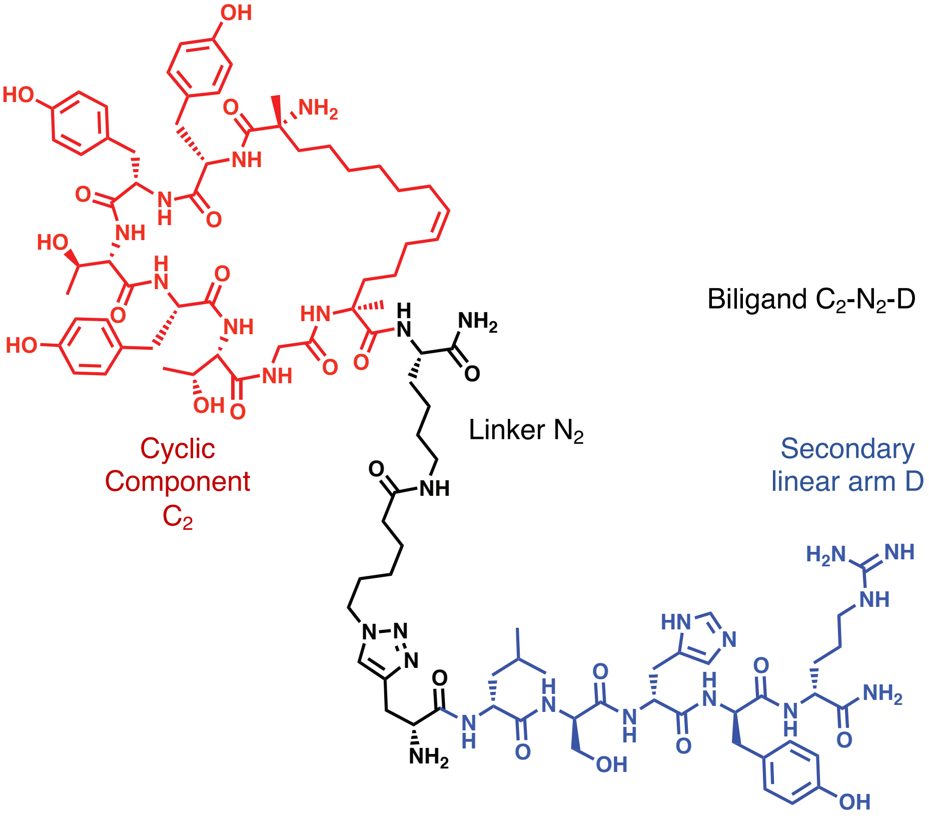
Biligand C2-N2-D
Linker N2
Cyclic Component C2
Secondary linear arm D

Figure S9: Biligand $B_{2,2,1}$ is selected by comparing ELISA binding assays of biligands to pAkt2 protein in buffer and in 1% serum.

1.25 μM of the biotin conjugated biligands were immobilized onto Streptavidin ELISA plates. A PEG_5-biotin was used as the no-ligand blank. The wells are treated with 25 nM His_6-Akt in buffer and in buffer with 1% bovine serum overnight. The wells are treated consecutively with mouse His_6 antibody, anti-mouse IgG conjugated to horse radish peroxidase (HRP) and HRP substrate TMB. The absorbance at 450 nm is measured to quantitate the binding. All four biligands $\mathbf{B_{2,2,1}}$ **(C_2-N_2-A)**, **C_2-N_2-B**, **C_2-N_2-C** and **C_2-N_2-D**, with secondary arms L_1, B, C and D bind with more signal to background ratio to pAkt2 protein compared to the best monoligand C_2. The biligand $\mathbf{B_{2,2,1}}$ is chosen as the best biligand based on the serum selectivity.

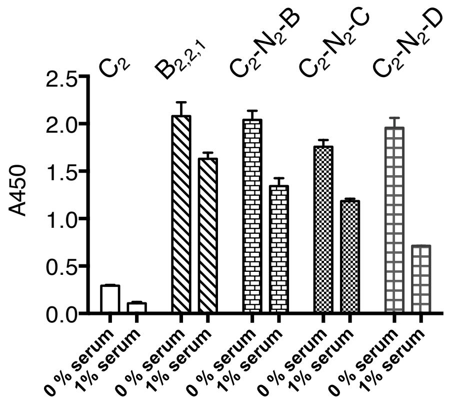

Figure S10: Binding affinity of biligand $B_{2,2,1}$ to pAkt2 protein determined by sandwich ELISA assay.

Biotinylated biligand $\mathbf{B_{2,2,1}}$ was immobilized on a High-Capacity Streptavidin 96 well plate (Thermo Scientific) Serial dilutions of active His6-tagged Akt2 were added per well the plate shaken overnight at 4°C. The wells were treated with a 1:1000 dilution of anti 6X His mouse monoclonal [HIS.H8] antibody (Abcam, ab18184), followed by1:10,000 dilution of goat anti-mouse antibody-Horse Radish Peroxide conjugate, treated with TMB substrate and quenched, and the absorbance at 450 nm wavelength measured. From the absorbance values at 450 nm wavelength (A450) for each protein concentration, the corresponding blank A450 (no ligand immobilized, same concentration of protein added) was subtracted.

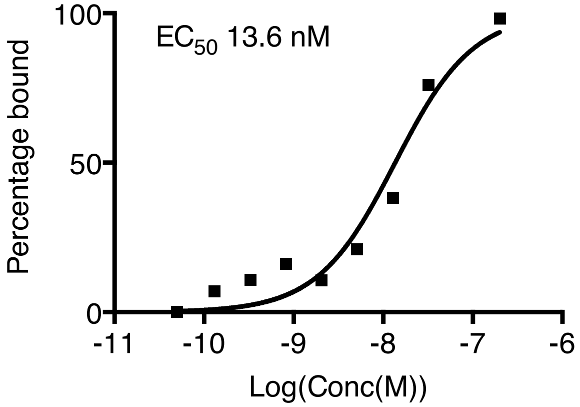

Figure S11: Biligand $B_{2,2,1}$ binds to p-S474 site on pAkt2.

2 μM biotinylated $\mathbf{B_{2,2,1}}$ or Ac-gly-biotin control was added to a streptavidin plate. After blocking, a series of solutions of 25 nM His_6-Akt2 and serial dilutions of the pS473 antibody (Cell Signaling) from 1/1000 to 1/20,000 was added to the plate and treated overnight. Following treatment with mouse anti-His6 antibody, goat anti-mouse antibody conjugated to Horse Radish Peroxide and TMB substrate, the reactions were quenched and absorbance values at 450 nm (A450) were measured. Net A450 was obtained by subtracting the corresponding absorbance values for the blank control (ac-gly-biotin, treated with same solutions of Akt and antibody dilutions) from the observed absorbance values. The graph demonstrates that biligand $\mathbf{B_{2,1,1}}$ competes for binding with the anti-pS473 Akt antibody (which detects Akt1, Akt2, and Akt3 protein) for binding to the pAkt2. Therefore, biligand $\mathbf{B_{221}}$ binds to the pS474 region of pAkt2.

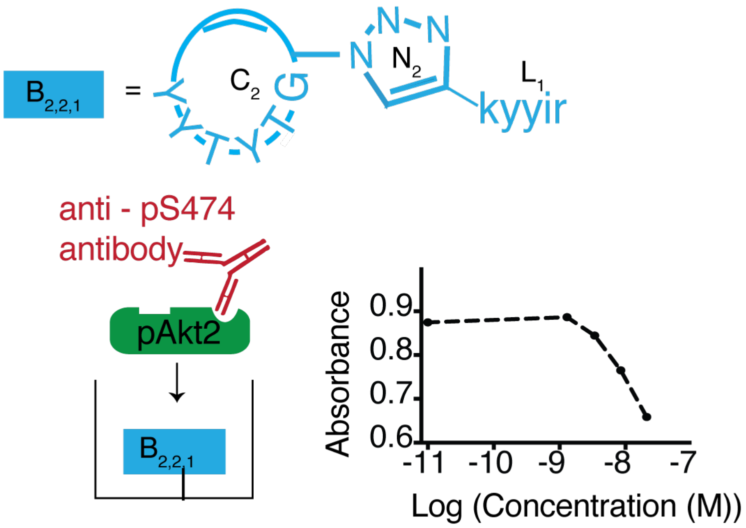

Figure S12: Biligand $B_{2,2,1}$ binds to pAkt2, almost similarly to pAkt1 and minimally to pAkt3 and inactive Akt2.

Biotinylated biligand $\mathbf{B_{2,2,1}}$ was immobilized on a Streptavidin plate. 25 nM solutions of active His6-tagged pAkt2, His6-tagged pAkt1, His6-tagged pAkt3 and inactive His_6-tagged pAkt2 were added and incubated overnight at 4°C. The wells were treated with anti 6X His mouse monoclonal antibody, goat anti-mouse antibody-Horse Radish Peroxide conjugate, TMB substrate and quenched, and the absorbance at 450 nm wavelength measured. From the absorbance values at 450 nm wavelength (A450) for each protein concentration, the corresponding blank A450 (Ac-gly-biotin immobilized, same concentration of protein added) was subtracted.

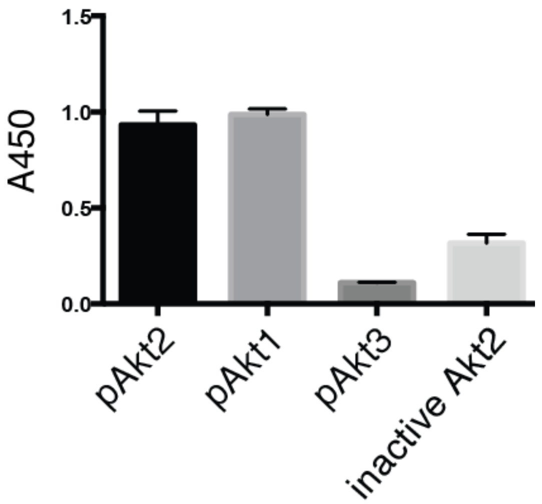

Figure S13: Inhibition of Akt kinase activity by biligand $B_{3,2,1}$.

A. Inhibition of Akt substrate GSK3 phosphorylation as determined by western blot. A dilution series of $\mathbf{B_{3,2,1}}$ mixed with pAkt2 in kinase reactions were set up using the protocol for the non–radioactive kinase assay kit for Akt2. Reactions were allowed to proceed for 30 minutes at 30°C, quenched, and gel electrophoresis was run on the denatured reaction solutions. Following the transfer of the gel to a nitrocellulose membrane, the membrane was treated with PhosphoGSK-3α/β Ser (21/9) rabbit antibody, mouse anti-rabbit –HRP antibody and ECL substrate and imaged on film; B. HTRF assay of Akt inhibition to determine IC_{50} of inhibition of Akt2 by ligand $\mathbf{B_{3,2,1}}$. Briefly, kinase, STK peptide substrate, ligand inhibitor, and ATP solutions were made per the given protocol for the HTRF kit. Following incubation at room temperature for a half hour, SaXL665 and STK Ab-cryptate were added, and fluorescence was measured at 620 nm (cryptate) and 665 nM (XL665). The A665/A620 ratio was calculated for each well.

A.

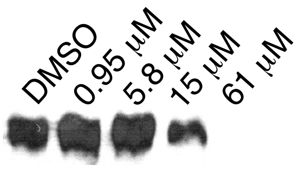

B.

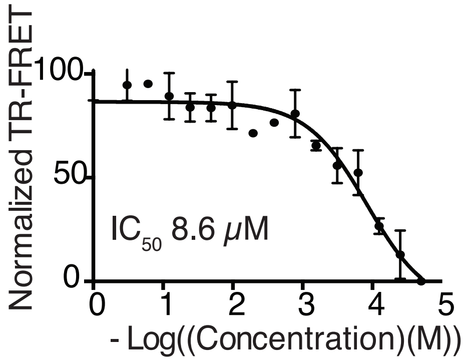

Figure S14: Alanine scan of cyclic monoligand C_2.

Biotinylated cyclic peptides with Ala replacing each amino acid sequentially– Cy(AYTYTG-rcm), Cy(YATYTG-rcm), Cy(YYATG-rcm), Cy(YYTATG-rcm), Cy(YYTYAG-rcm), Cy(YYTYTA-rcm) and biotinylated C2 were immobilized on a streptavidin plate and treated with 25 nM His6-pAkt2 overnight at 4°C. The wells were treated with anti 6X His mouse monoclonal antibody, goat anti-mouse antibody-Horse Radish Peroxide conjugate, TMB substrate and quenched, and the absorbance at 450 nm wavelength measured. From the absorbance values at 450 nm wavelength (A450) for each protein concentration, the corresponding blank A450 (Ac-gly-biotin immobilized, same concentration of protein added) was subtracted. Replacement of each tyrosine was found to significantly negate binding to the protein, indicating the important role Tyr place in protein-ligand binding. There was loss of binding on replacement of Thr and Ala, though to a lesser extent.

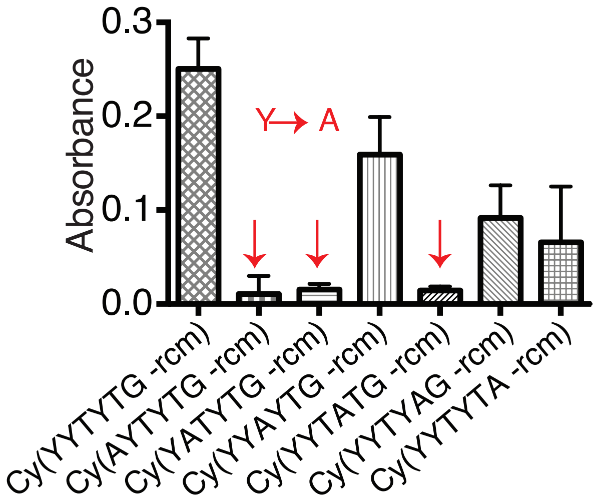

Figure S15: Effect of binding to Akt2 protein for 4-fluorophenylalanine substituted derivatives of C_2 (Cy(YYTYTG-rcm)).

Replacement of Tyrosine by 4-fluorophenylalanine at position 1, 2 and for 4 results in ligands Cy(4fF-YTYTG-rcm), Cy(-Y4fF-TYTG-rcm) and Cy(YYT-4fF-TG-rcm). The ligands, along with **C_2**, with sequence Cy(YYTYTGrcm), were immobilized on a streptavidin plate and treated with 25 nM His6-pAkt2 overnight at 4°C. The wells were treated with anti 6X His mouse monoclonal antibody, goat anti-mouse antibody-Horse Radish Peroxide conjugate, TMB substrate and quenched, and the absorbance at 450 nm wavelength was measured. From the absorbance values at 450 nm wavelength (A450), the corresponding blank A450 (Ac-gly-biotin immobilized, 25 nM protein added) was subtracted to obtain the corrected absorbance values. The first two ligands containing 4fF have a higher signal than C_2, whereas replacing the third Tyr by 4flF in the ligand decreases binding. The ligand Cy(4fF-4fF-TYTG-rcm) with dual substitution of tyrosines at positions 1 and 2 also has higher binding than **C_2**.

4fF:

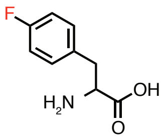

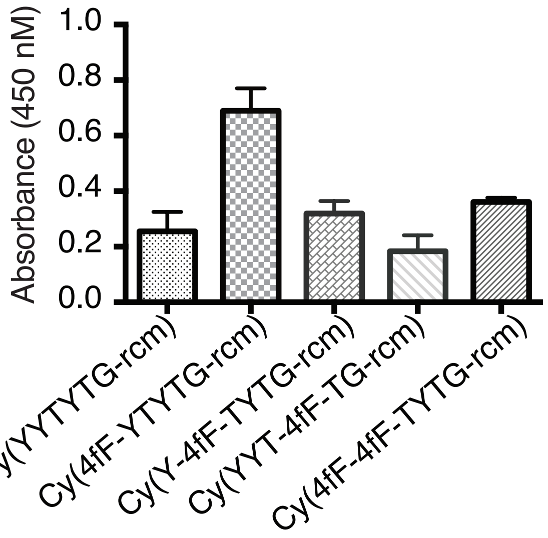

Figure S16: N-methylation of C_2 negates binding of the ligands to pAkt2.

Biotin-tagged ligands **C_2** and N-methylated cyclic ligands Cy(YYTY’T’G*rcm*) and Cy(Y’YTY’TG*rcm*) were immobilized on a streptavidin plate and treated with 25 nM His6-pAkt2 overnight at 4°C. The wells were treated with anti 6X His mouse monoclonal antibody, goat anti-mouse antibody-Horse Radish Peroxide conjugate, TMB substrate and quenched, and the absorbance at 450 nm wavelength was measured. From the absorbance values at 450 nm wavelength (A450), the corresponding blank A450 (Ac-gly-biotin immobilized, 25 nM protein added) was subtracted to obtain the corrected absorbance values. Both the N-methylated ligands show no binding to the pAkt2 ligand.

Y’:

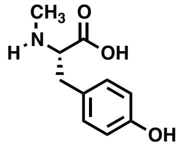

T’:

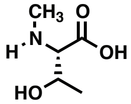

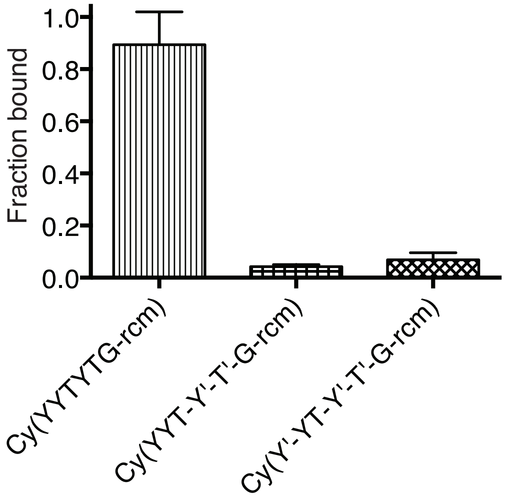

Figure S17: Replacement of linker Nj by polyalanine linker and α,α-disubstituted α-amino acid.

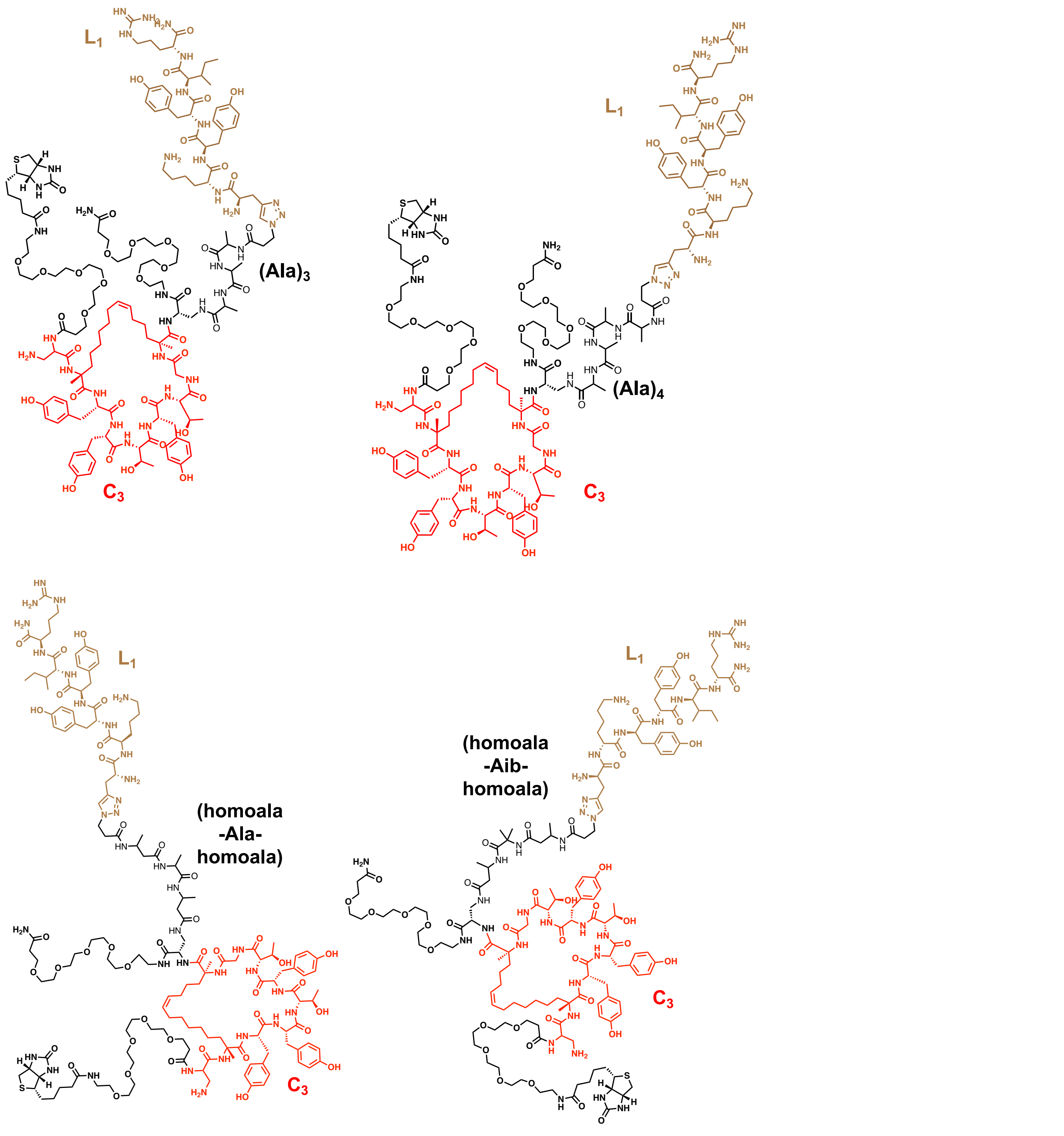

Figure S18: Molecular structures of biligands in which Nj is replaced by PEG linker or arginine is incorporated into Nj or rigid aromatic residues are incorporated in Nj.

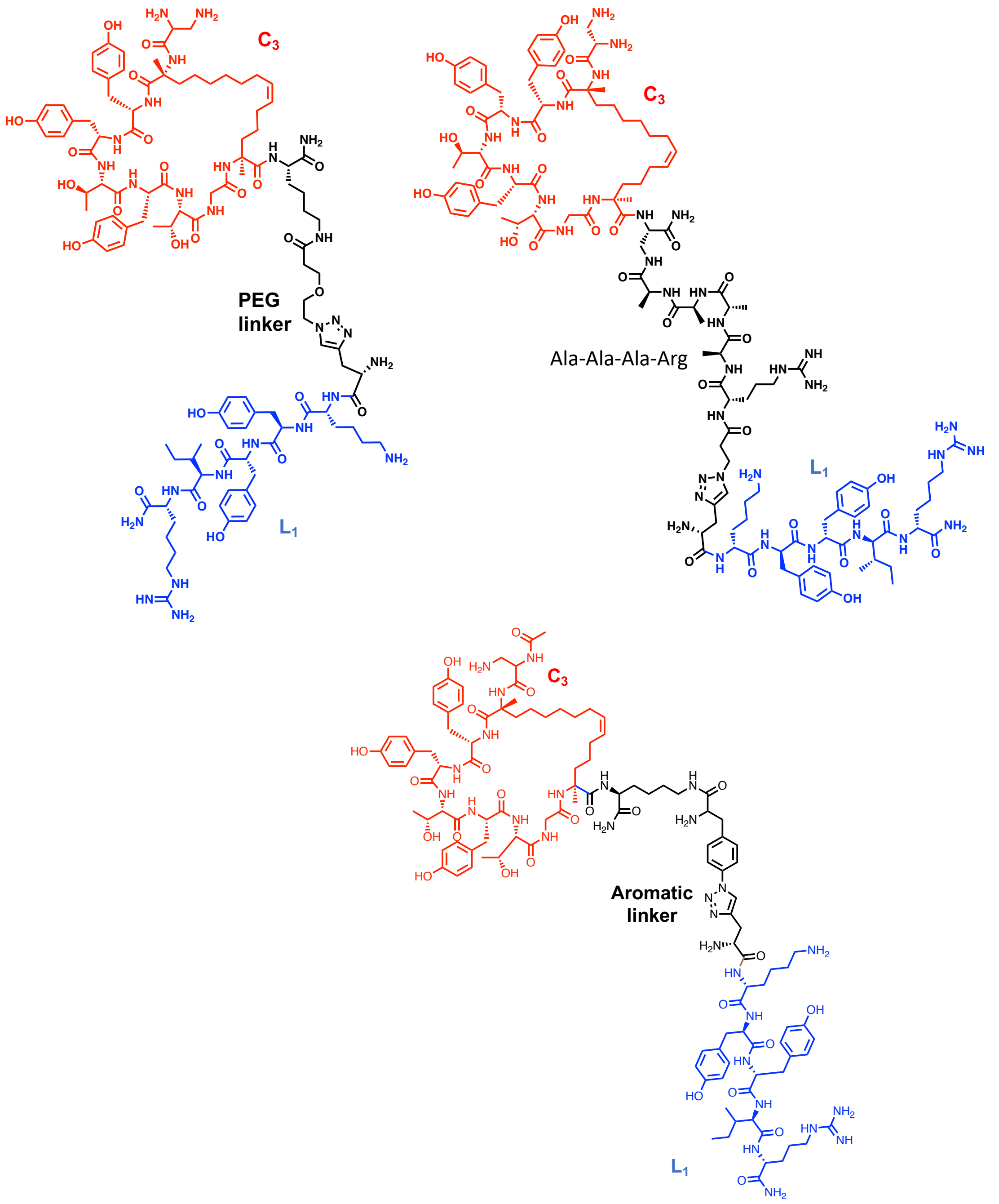

Figure S19: Molecular structures of N-methylated biligands $B_{3,2,2}$ – $B_{3,2,5}$.

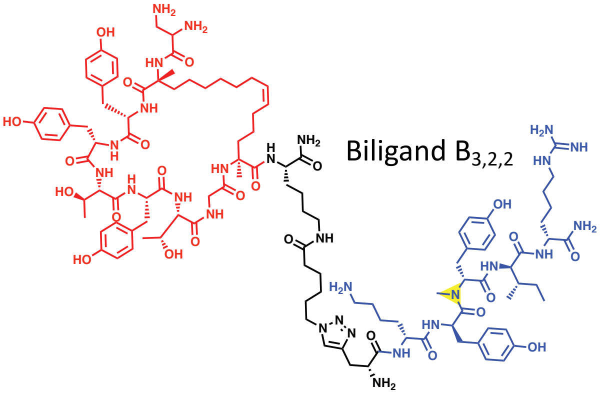

Biligand $B_{3,2,2}$

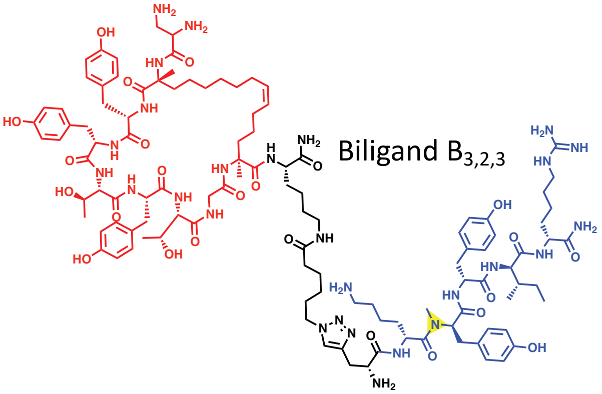

Biligand $B_{3,2,3}$

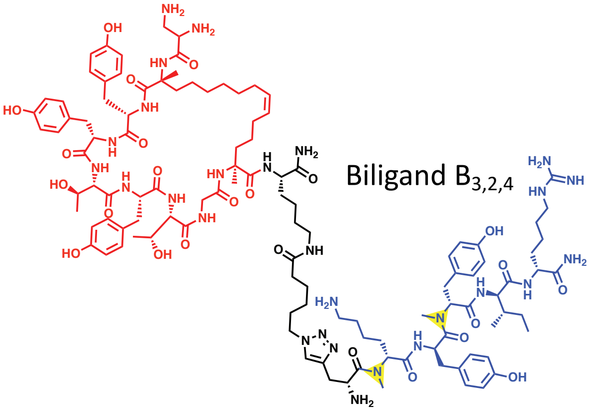

Biligand $B_{3,2,4}$

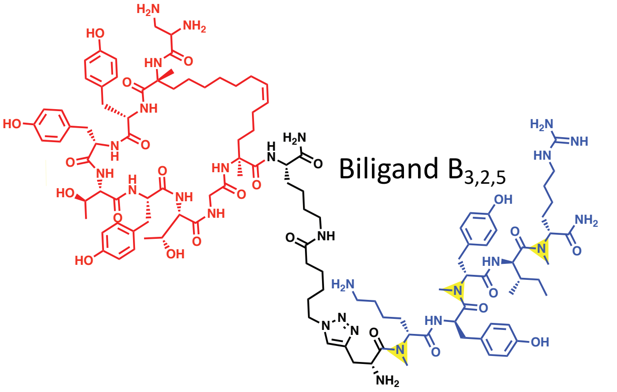

Biligand $B_{3,2,5}$

Figure S20: Biligands $B_{3,2,2}$ – $B_{3,2,5}$ which have N-methylated amino acids incorporated in the linear component, show binding to pAkt2.

Biotin-tagged biligands **$B_{3,2,2}$** – **$B_{3,2,5}$**, containing one, two or three N-methylated amino acids in the linear arm were immobilized on a streptavidin plate and treated with a serial dilution of His_6-pAkt2 overnight at 4°C. The wells were treated with anti 6X His mouse monoclonal antibody, goat anti-mouse antibody-Horse Radish Peroxide conjugate, TMB substrate and quenched, and the absorbance at 450 nm wavelength measured. From the absorbance values at 450 nm wavelength (A450) the corresponding blank A450 (Ac-gly-biotin immobilized, same concentration series of protein added) was subtracted to obtain the corrected absorbance values. While **$B_{3,2,2}$-$B_{3,2,5}$** all show binding to pAt2, **$B_{3,2,5}$** has minimal background binding, unlike the other N-methylated biligands.

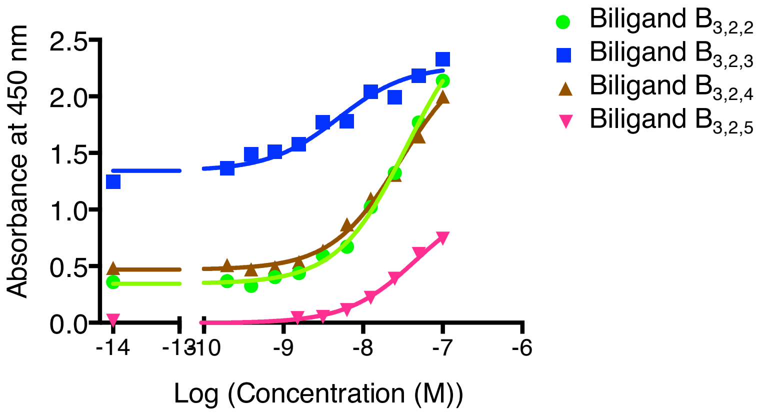

Figure S21: Molecular structure of biligand $B_{4,2,5}$.

Biligand $\mathbf{B_{4,2,5}}$ has triple N-methylation in the linear arm, incorporates fluorophenyl alanine instead of tyrosine twice in the cyclic component and has N-terminal DAP.

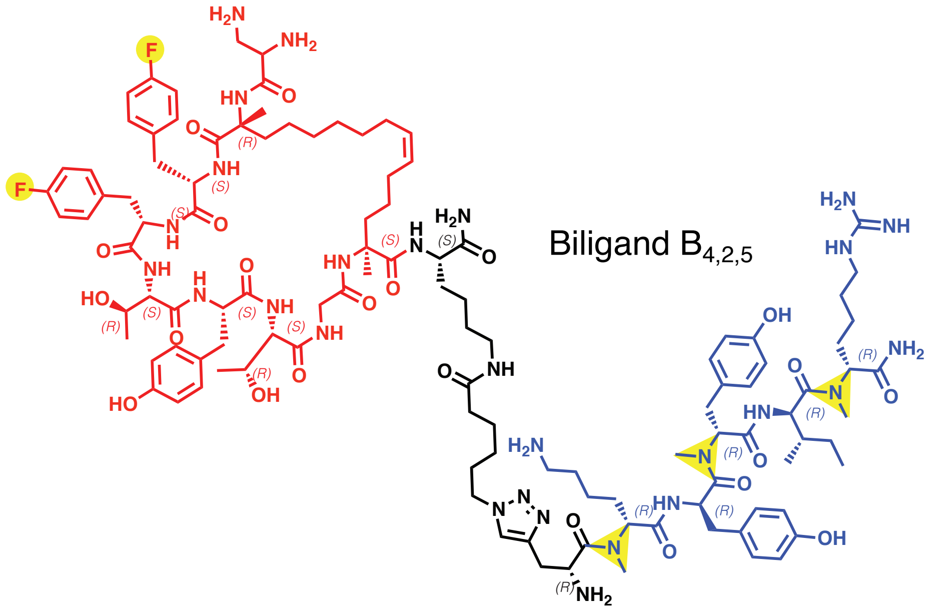

Figure S22: Biligand $B_{4,2,5}$ demonstrates good binding to pAkt2 protein (EC_{50} 60.6 nM)

Biotin-tagged biligand $\mathbf{B_{4,2,5}}$ was immobilized on a streptavidin plate and treated with a serial dilution of His_6-pAkt2 overnight at 4°C. The wells were treated with anti 6X His mouse monoclonal antibody, goat anti-mouse antibody-Horse Radish Peroxide conjugate, TMB substrate and quenched, and the absorbance at 450 nm wavelength measured. From the absorbance values at 450 nm wavelength (A450) the corresponding blank A450 (Ac-gly-biotin immobilized, same concentration series of protein added) was subtracted to obtain the corrected absorbance values.

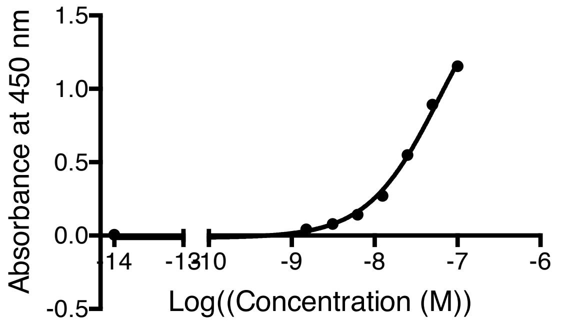

Figure S23: MD simulations of $B_{4,2,5}$.

The optimized conformation of the chiral centers of the biligands in solvents: n-hexane (A) and water (B). The analysis of non-bonded interactions between the chiral centers CC1 & CC2 in the macrocycle of the biligands (**$B_{7,3,5}$** (purple), **$B_{5,3,5}$** (green), and **$B_{4,2,5}$** (orange)) and solvents (n-hexane (C) and water (D)) obtained from MD simulations. All the presented distances are the averaged values over the course of MD simulations.

n-Hexane

Water

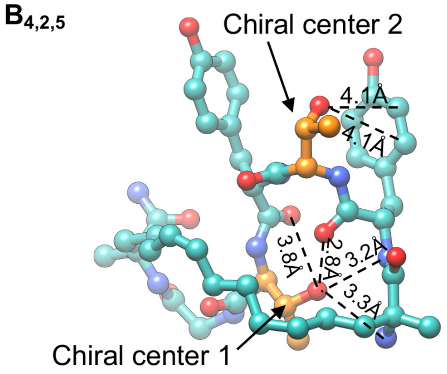

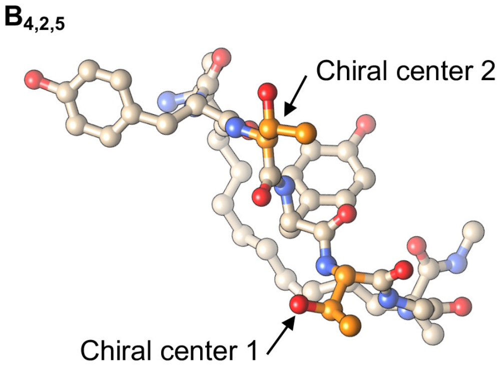

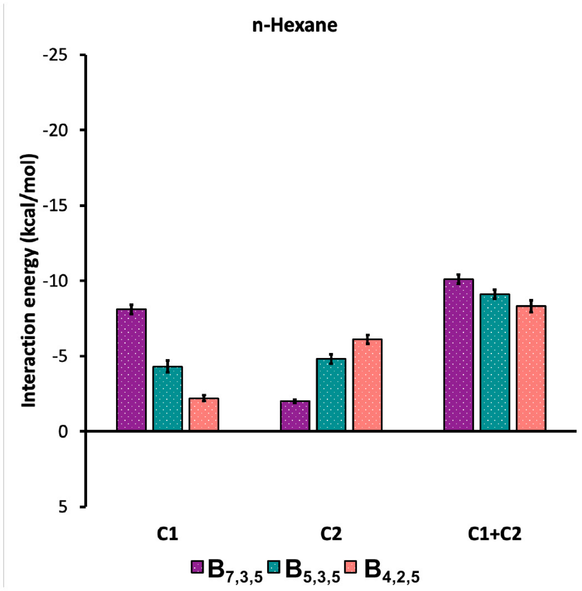

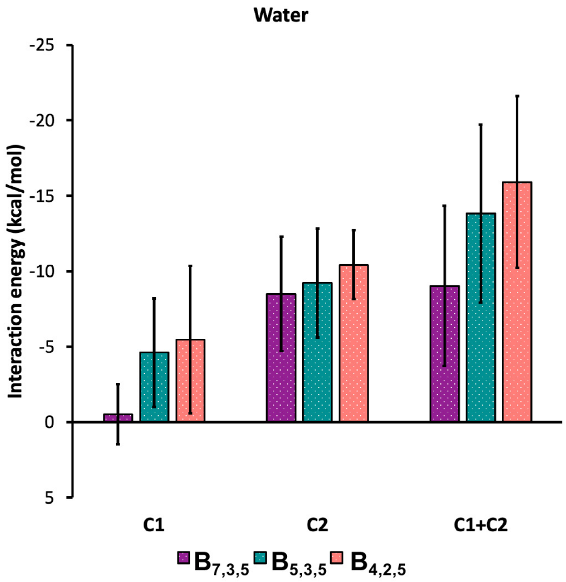

Figure S24: Understanding retro-inversion of the cyclic component C_5 to C_6

Invert chirality

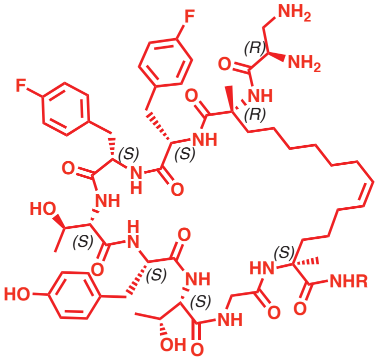

(D-dap)-Cy(4fF-4fF-TYTG-*rcm*)

rcm: alkenyl ring closure synthesized by Ruthenium catalyzed Ring Closing Metathesis reaction between ***(R)***- α-(7-Octenyl)alanine and ***(S)***-2-(4-pentenyl)alanine

(D-dap): D-diaminopropionic acid

4fF: 4-fluorophenylalanine

retro

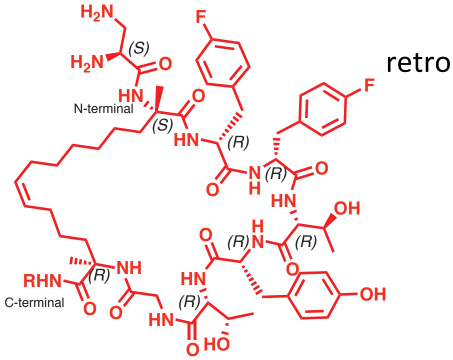

(L-Dap)-Cy(4fF-4fF-TYTG-*rcm'*)

rcm': alkenyl ring closure synthesized by Ruthenium catalyzed Ring Closing Metathesis reaction between ***(S)***- α-(7-Octenyl)alanine and ***(R)***-2-(4-pentenyl)alanine

(L-dap): L-diaminopropionic acid

D-4fF: D-4-fluorophenylalanine

Invert sequence

Retro-inverted

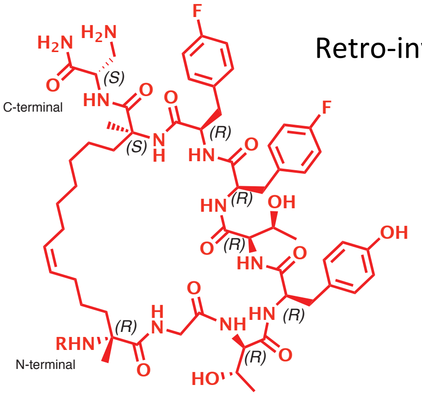

Cy(*rcm'*–Gtyt-(D-4fF)-(D-4fF))-L-Dap

Figure S25: Molecular structures of retro-inverted biligands $B_{5,3,4}$, $B_{5,3,5}$, $B_{6,3,4}$ and $B_{6,3,5}$.

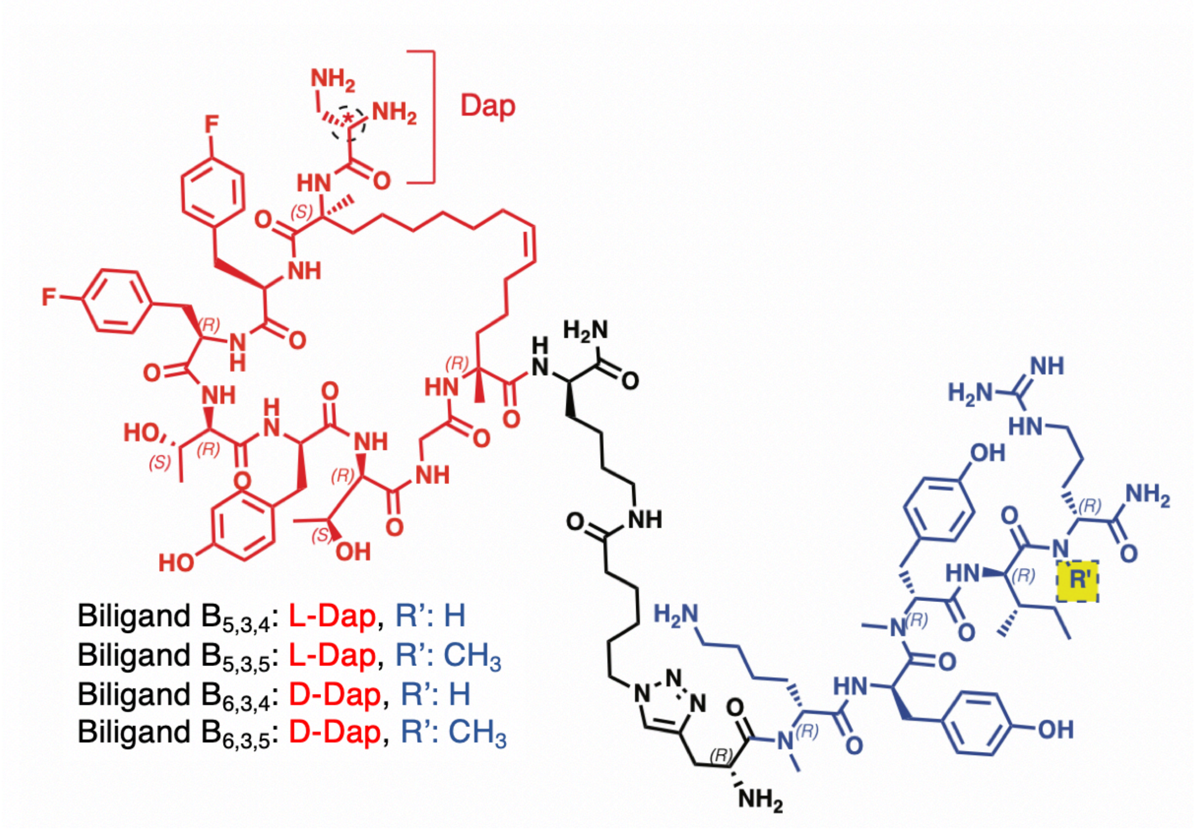

Figure S26: Retro-inverted biligands $B_{6,3,4}$ and $B_{6,3,5}$ demonstrate excellent binding characteristics (EC_{50} 10.8 and 7.05 nM) for pAkt2.

Retro-inverted biotin-tagged biligands $\mathbf{B_{6,3,4}}$ and $\mathbf{B_{6,3,5}}$ were immobilized on a streptavidin plate and treated with serial dilutions of His_6-pAkt2 overnight at 4°C. The wells were treated with anti 6X His mouse monoclonal antibody, goat anti-mouse antibody-Horse Radish Peroxide conjugate, TMB substrate and quenched, and the absorbance at 450 nm wavelength was measured. From the absorbance values at 450 nm wavelength (A450), the corresponding blank A450 (Ac-gly-biotin immobilized, same concentration series of protein added) was subtracted to obtain the corrected absorbance values.

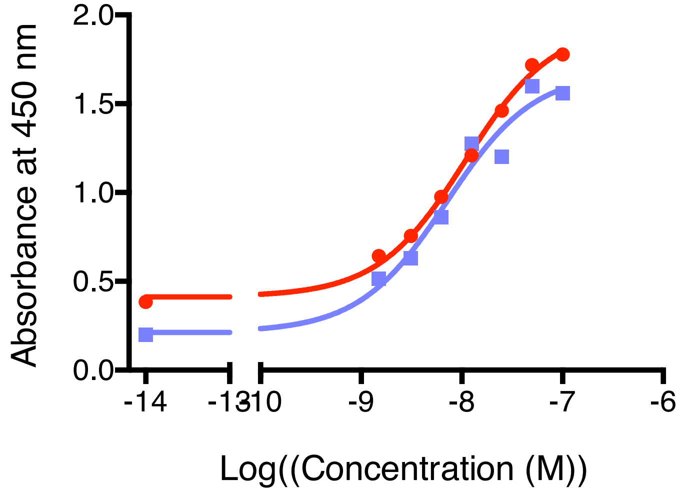

Figure S27: Biligand $B_{7,3,5}$, containing D-(2R,3R) threonines and biligand $B_{5,3,5}$, containing D-(2S,3R) threonines, have similar affinities (EC_{50} 11.80 nM and 15.06 nM respectively) for pAkt2 protein.

Biotin-tagged biligands retro-inverso $\mathbf{B_{5,3,5}}$ and $\mathbf{B_{7,3,5}}$ was immobilized on a streptavidin plate and treated with serial dilutions of His_6-pAkt2 overnight at 4°C. The wells were treated with anti 6X His mouse monoclonal antibody, goat anti-mouse antibody-Horse Radish Peroxide conjugate, TMB substrate and quenched, and the absorbance at 450 nm wavelength measured. From the absorbance values at 450 nm wavelength (A450) the corresponding blank A450 (Ac-gly-biotin immobilized, same concentration series of protein added) was subtracted to obtain the corrected absorbance values.

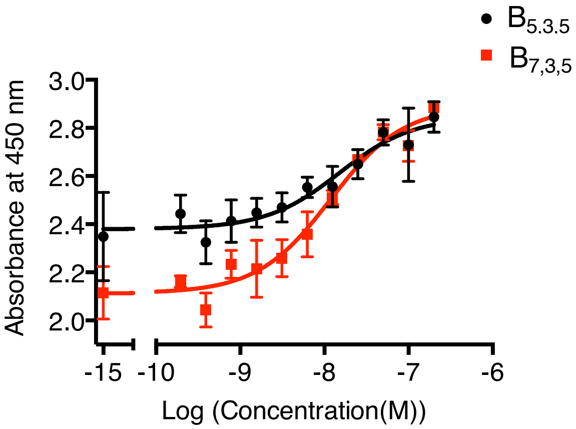

Figure S28: Pulldown using retro-inverted ligands $\mathbf{B}_{5,3,5}$ and $\mathbf{B}_{7,3,5}$ as immunoprecipitation reagents.

Biotin-tagged $\mathbf{B}_{5,3,5}$ and $\mathbf{B}_{7,3,5}$ ligands were immobilized on Sa-agarose beads and treated with non-stimulated or EGF-stimulated OVCAR3 cell lysate. Biotinylated glycine (gly-biotin) was used as the blank control ligand. SDS-PAGE gels were run on the immunoprecipitated proteins, and the gels were treated with Coomassie stain or subjected to western blot. Akt has a mass of 60 kD. The Coomassie stain shows that the proteins immunoprecipitated by biotin-tagged $\mathbf{B}_{5,3,5}$ and $\mathbf{B}_{7,3,5}$ are similar to that precipitated by the blank control beads immobilized with glycine-biotin.

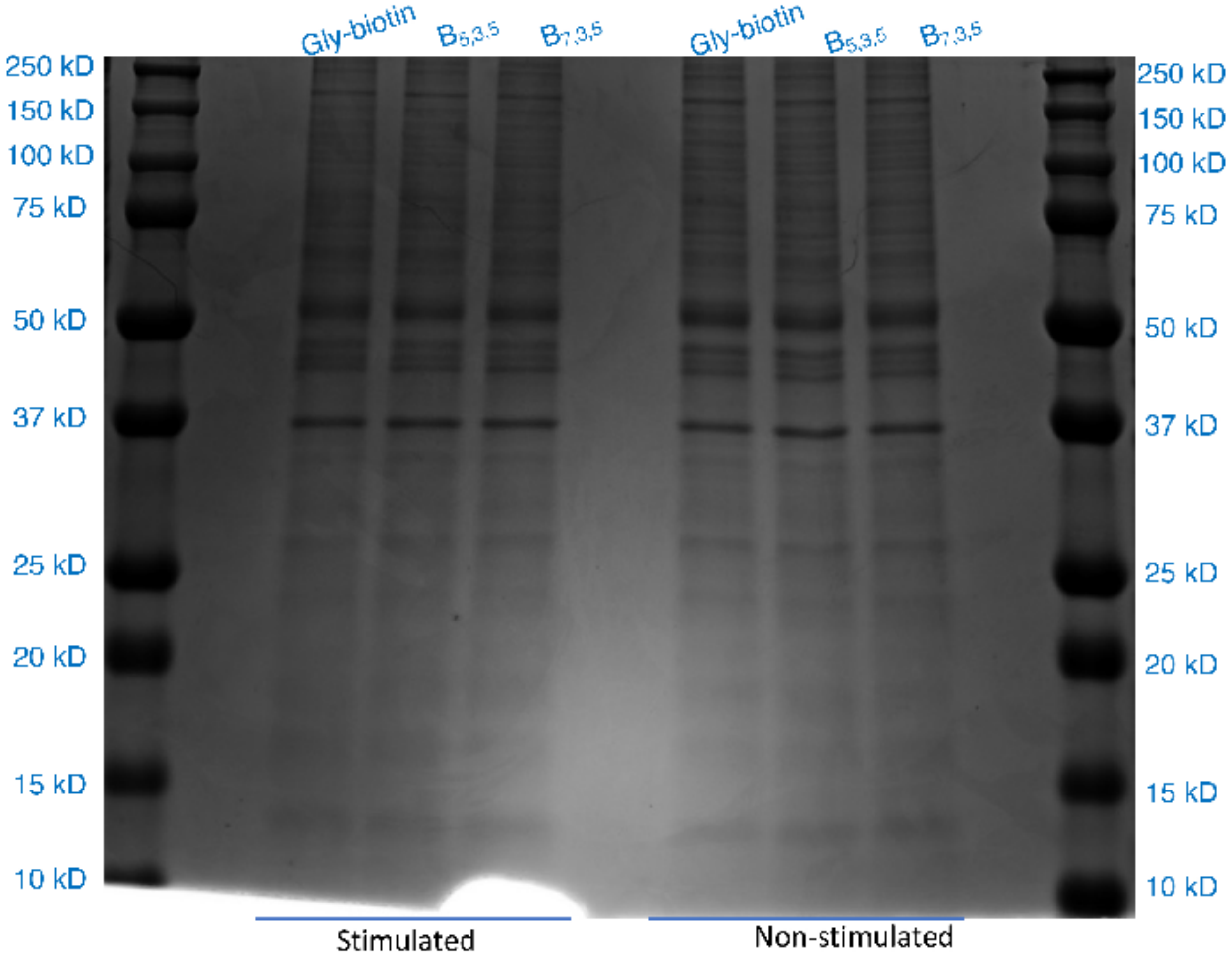

Figure S29: Fluorescence microscopy of non-stimulated OVCAR3 cells with fluorescein labeled $B_{7,3,5}$

OVCAR3 cells were serum starved, treated with 100 nM fluorescein-labeled $\mathbf{B_{7,3,5}}$ for 12 hours at 37°C, washed with PBS and Accutase to remove extra ligand, treated with PI3K inhibitor Ly294002 for 30 minutes, and imaged in a chamber with 5% CO_2.

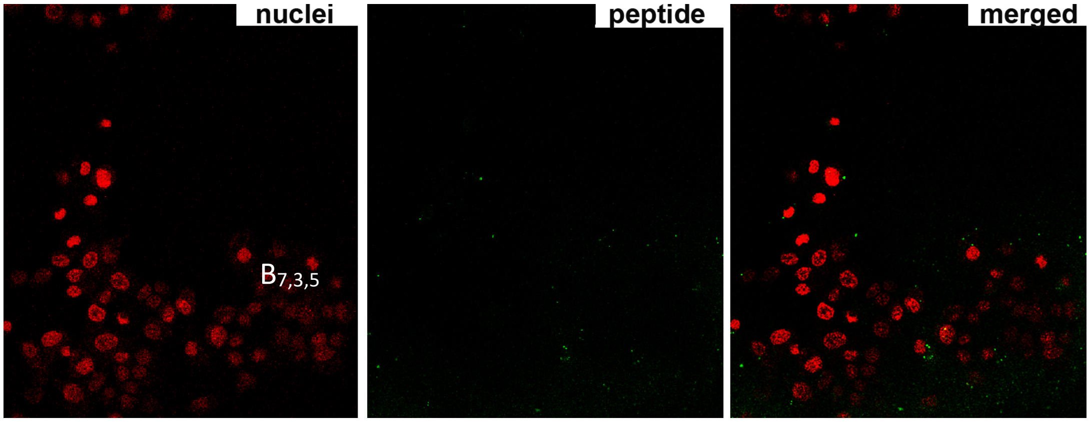

Figure S30: Solution-diffusion mechanism of the biligands, $B_{5,3,5}$ and $B_{7,3,5}$, cell penetrating characteristics.

(a) The optimized representative snapshots for the $\mathbf{B_{7,3,5}}$ biligand, showing the detailed steps in accord with the solution-diffusion mechanism that $\mathbf{B_{7,3,5}}$ takes to penetrate across the lipid membrane. (b) MetaD free energy profile for the solution mechanism of the biligands. Here, the collective variable is the distance between the center of mass of macrocycle to the center of mass of lipid membrane. (c) MetaD free energy profile for the diffusion mechanism of the biligands. Here, the collective variable is the distance between the center of mass of macrocycle to the center of mass of lipid membrane. The change in the free energy difference with time was calculated to monitor the free energy convergence. The weighted averages and the standard deviations were calculated after convergence.

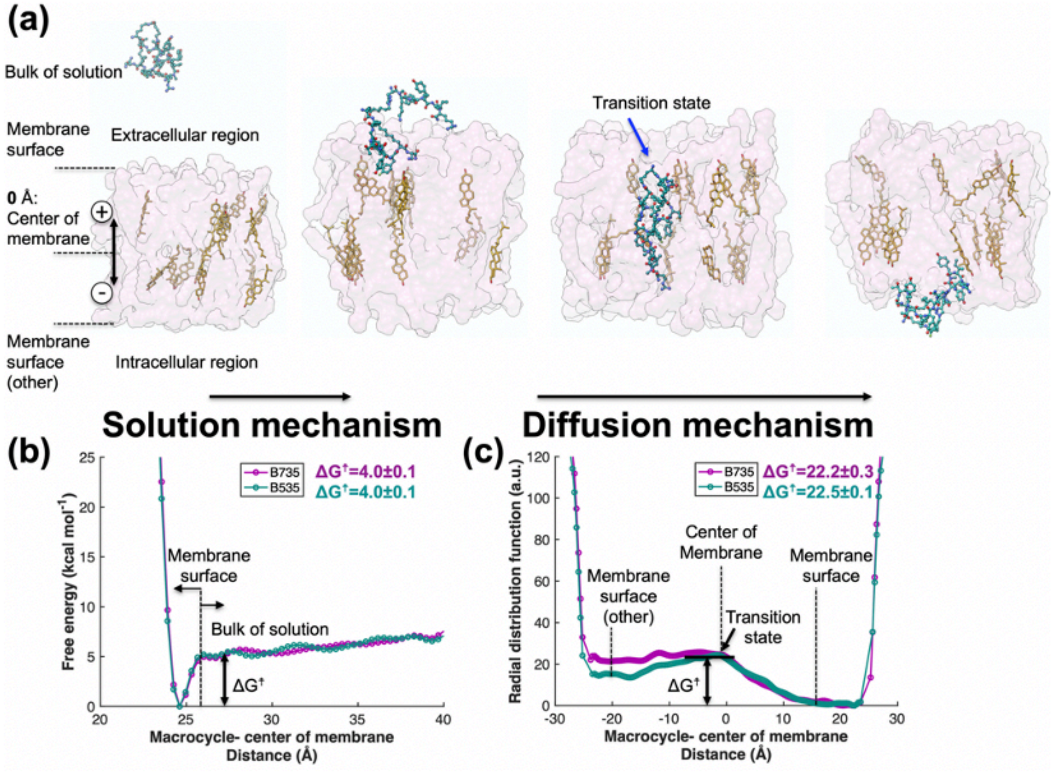

Figure S31: The analysis of non-bonded interactions between the macrocycle of the biligands ($B_{5,3,5}$ and $B_{7,3,5}$) and the components of the lipid membrane (POPC and cholesterol).

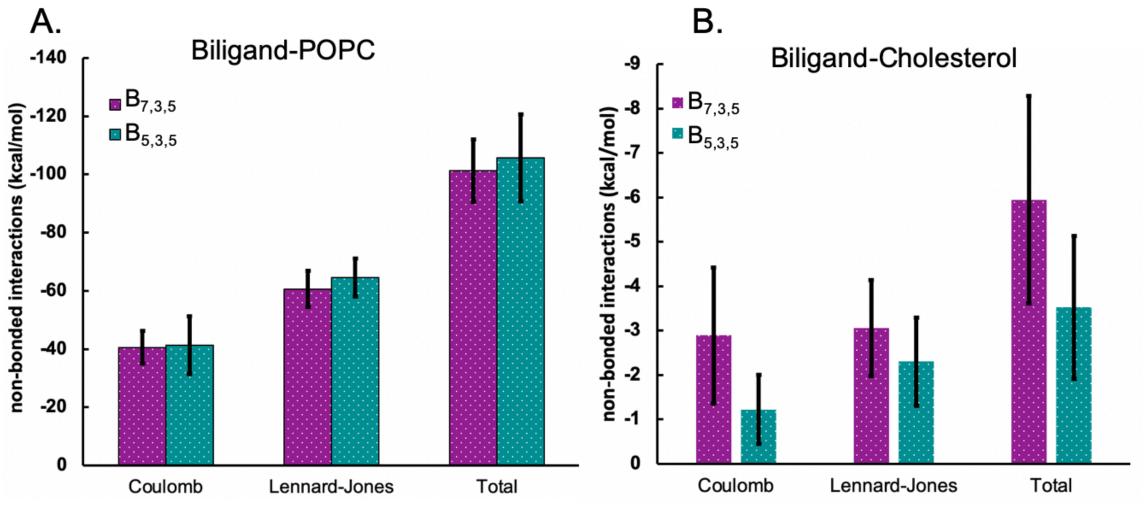

Figure S32: Flow cytometry gating strategy of NIH OVCAR3 cells treated with FITC-$B_{7,3,5}$.

Following the gating of NIH OVCAR3 cells in FSC vs. SSC dot blot, the populations were further gated for the elimination of cell dimers and aggregates. Gating of FITC+ cells was based on the staining of 7AAD only stained cells control and unstained cells control. The percentage of FITC+ cells was based on the frequency of the total.

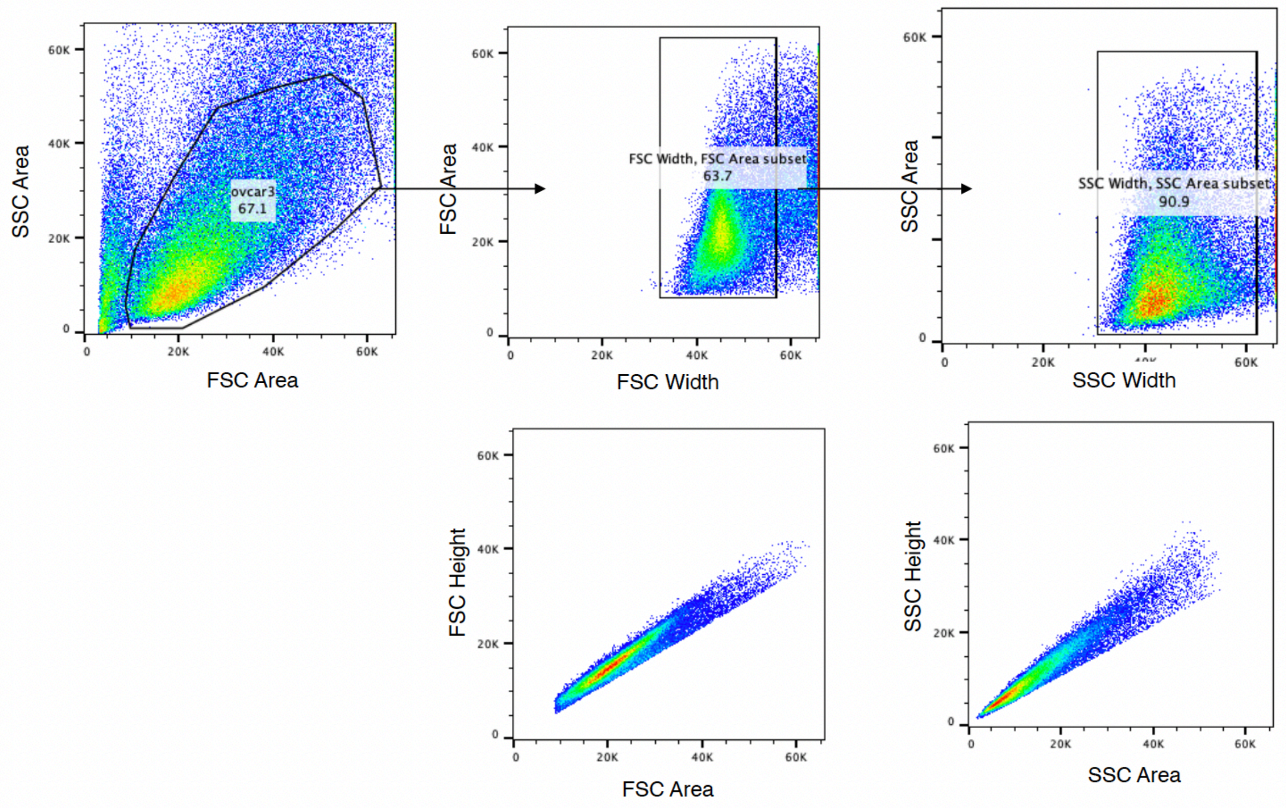

Mass spectroscopic characterization of peptides:

Mass spectrometry of target epitope His_6–Peg_2-ITPPDRYDSLG LLELDQRTH FPQF (pS)YSASIRE:

(figure S. 1) Calculated mass: 4858.1; Observed mass: 4858.1; ESI –TOF MS: m/z; 608.18 (M + 8H) $^{8+}$/8, 694.92 (M + 7H) $^{7+}$/7, 810.57 (M+6H) $^{6+}$/6, 972.67 (M+5H) $^{5+}$/5, 1215.35 (M+4H) $^{4+}$/4

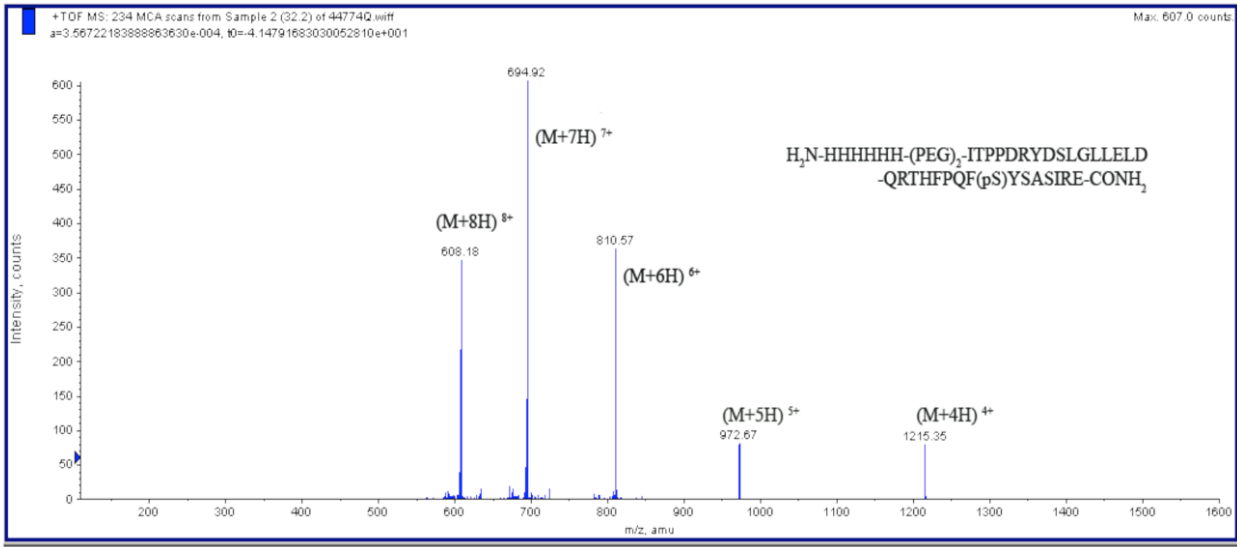

	Mass (avg.)	Mass (mono.)	Apex Mass	Area	Start Scan	Stop Scan	Score	Evidence
1	4858.1707	4855.4097	4858.3882	7506.0000	1	1	1.0000	IC

Mass spectrometric analysis of biotin tagged C_2:

(figure S.1–S.4, figures S.14-S.16) Expected mass: 1617.84; Observed mass:1617.88, m/z 809.94 $(M+2H^+)/2$

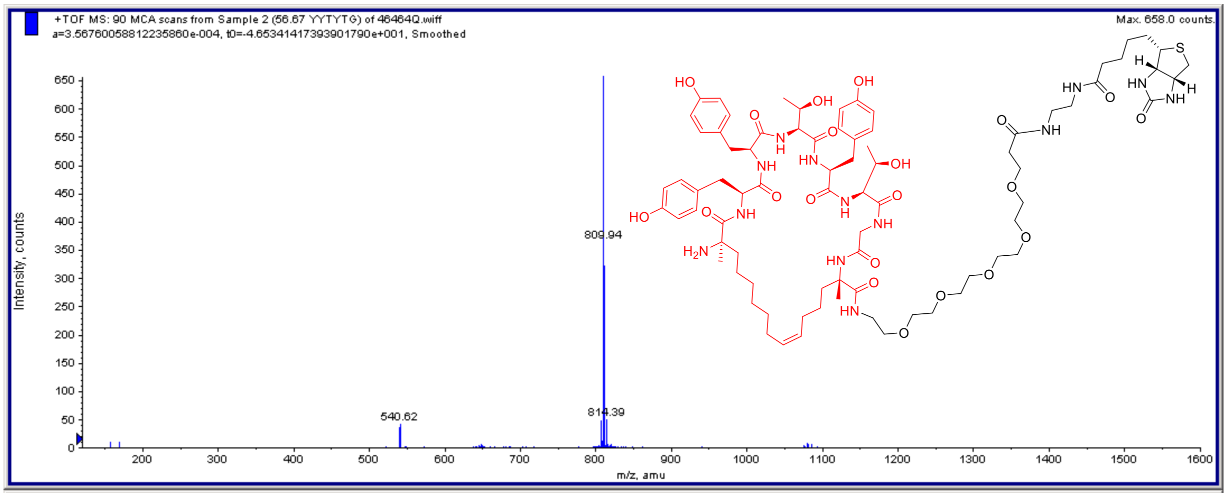

	Mass (avg.)	Mass (mono.)	Apex Mass	Area	Start Scan	Stop Scan	Score	Evidence
1	1618.8086	1617.8476	1618.8339	10003.0000	1	1	1.0000	IC

Mass spectrometric analysis of C_2-N_1:

(Scheme SC.2, figure S.7) Expected mass: 1885.1; Calculated mass:1885.06, m/z 943.53 $(M+2H^+)/2$, 629.36 $(M+3H^+)/3$

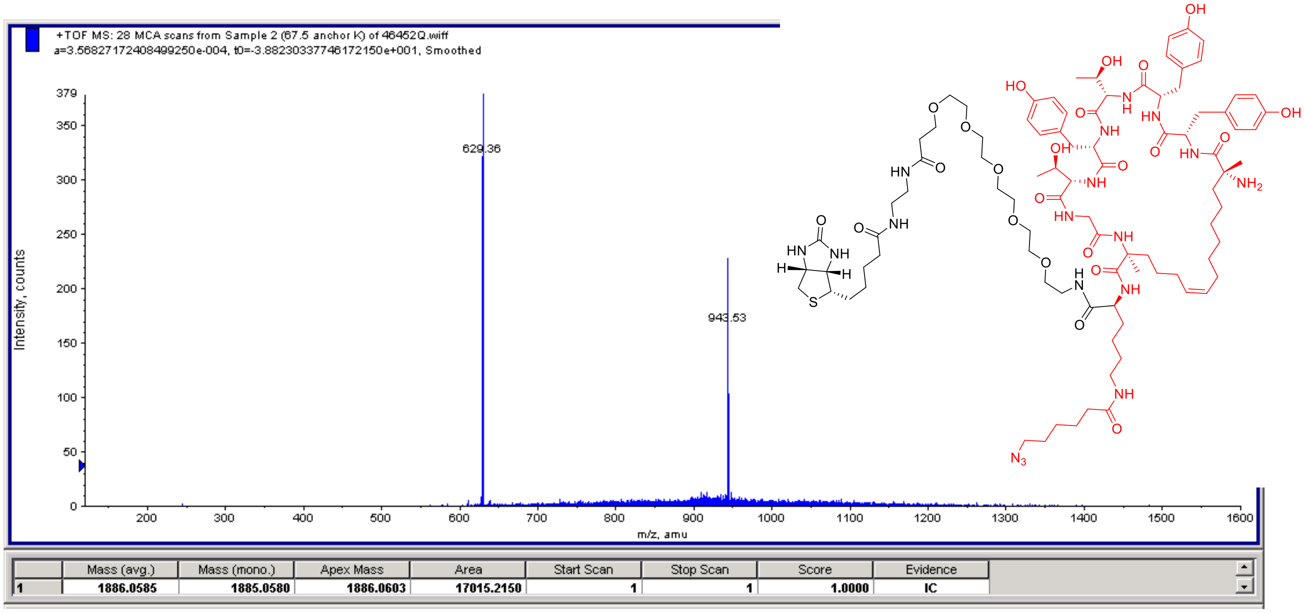

Mass spectrometric analysis of $B_{2,2,1}$:

(figure S.8-S.12) Expected mass:2722.49; Observed mass: 2722.02, m/z; 908.3 $(M + 3H)^{3+}/3$, 681.5 $(M + 4H)^{4+}/4$

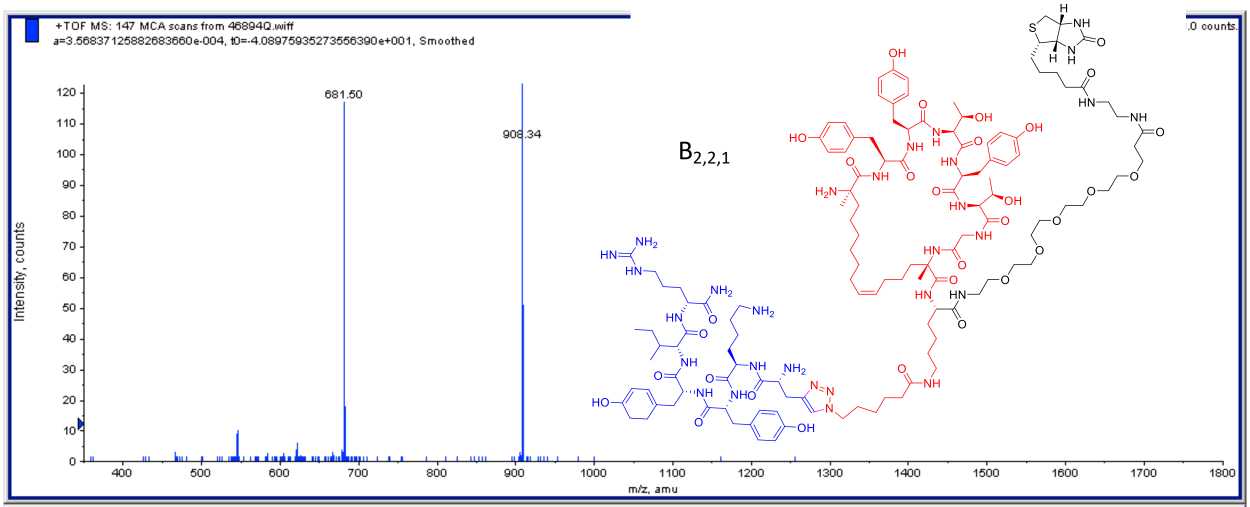

	Mass (avg.)	Mass (mono.)	Apex Mass	Area	Start Scan	Stop Scan	Score	Evidence
1	2722.7163	2720.9971	2722.9713	6766.2401	1	1	1.0000	IC

Mass spectrometric analysis of biligand C2-N2-B:

(figure S.8, figure S.9) Expected mass:2728; Observed mass: 2728.2, m/z 1365.1 $(M + 2H)^{2+}/2$. 910.2 $(M + 3H)^{3+}/3$

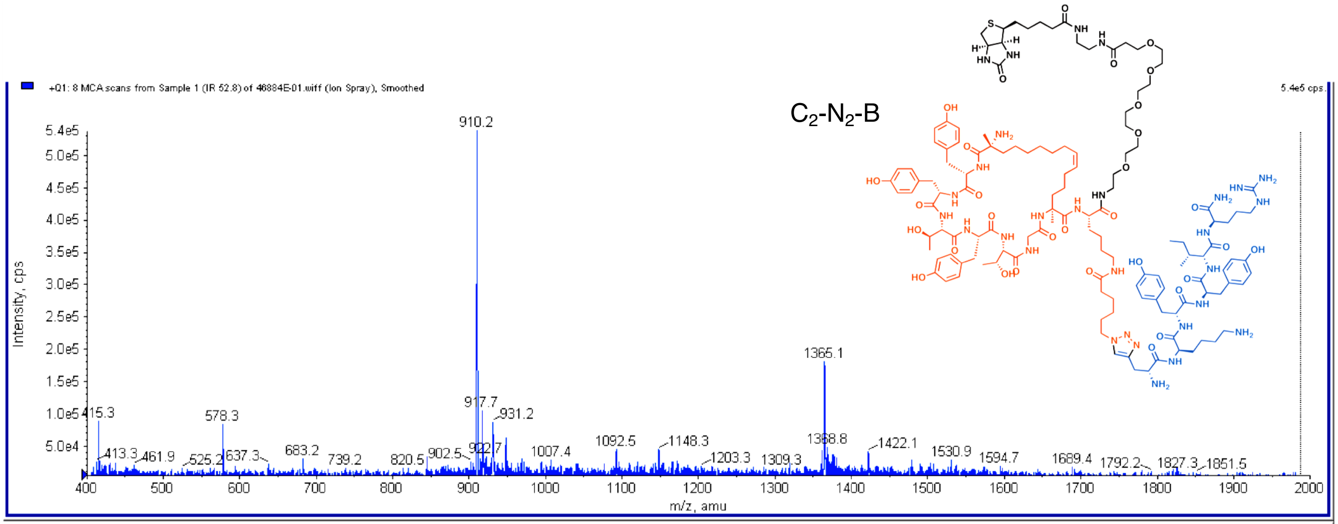

	Mass (avg.)	Mass (mono.)	Apex Mass	Area	Start Scan	Stop Scan	Score	Evidence
1	2728.6654		2727.5778	9759609.9375	1	1	1.0000	C

Mass spectrometric analysis of biligand C_2-N_2-C:

figure S.8, figure S.9) Expected mass: 2787.42, Observed mass: 2787.99, m/z; 930.33; $(M + 3H)^{3+}/3$, 698.0; $(M + 4H)^{4+}/4$ 558.83

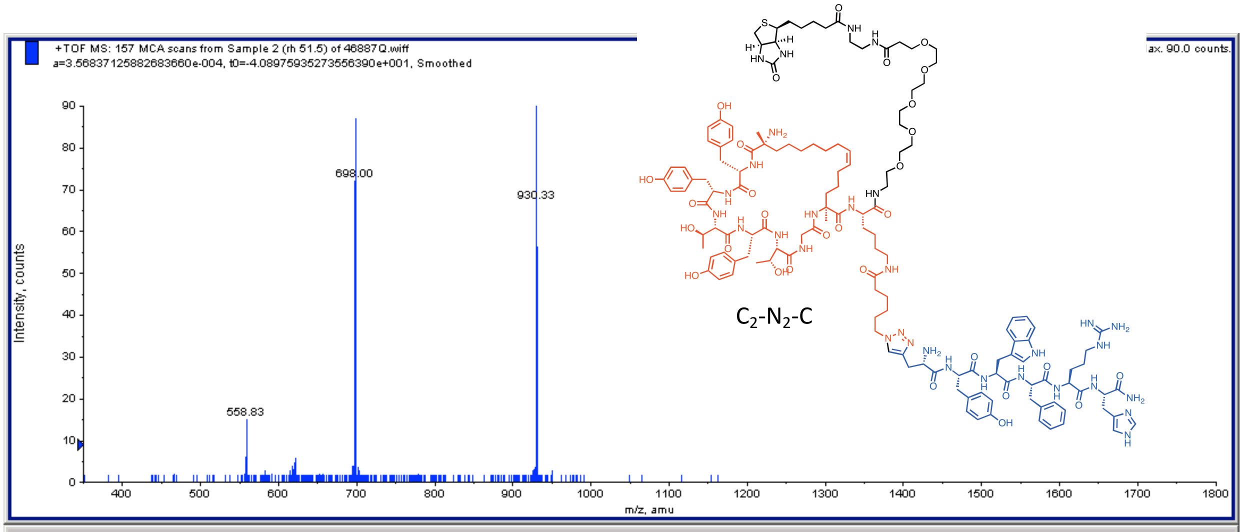

	Mass (avg.)	Mass (mono.)	Apex Mass	Area	Start Scan	Stop Scan	Score	Evidence
1	2789.0336	2787.9766	2788.9624	3402.0000	1	1	1.0000	IC

Mass spectrometric analysis of C_2-N_2-D:

(figure S.8, figure S.9) Expected mass: 2654.39 Observed mass: 2654.7 m/z; 1328.2 $(M + 2H)^{2+}/2$, 885.9 $(M + 3H)^{3+}/3$

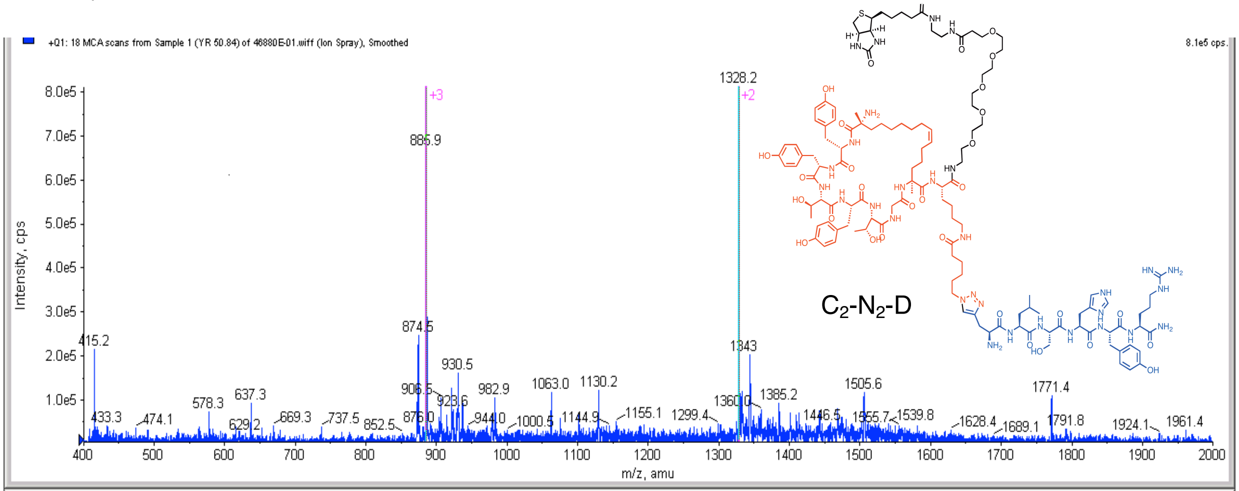

	Mass (avg.)	Mass (mono.)	Apex Mass	Area	Start Scan	Stop Scan	Score	Evidence
1	2654.7841		2654.7008	8862553.8750	1	1	1.0000	C
2	2686.1172		2686.1175	2544074.2500	1	1	0.0000	U

Mass spectrometric analysis of fluorescein conjugated biligand $B_{3,2,1}$

(figure 1B) Calculated mass:3389.96; Observed mass: 3389.64

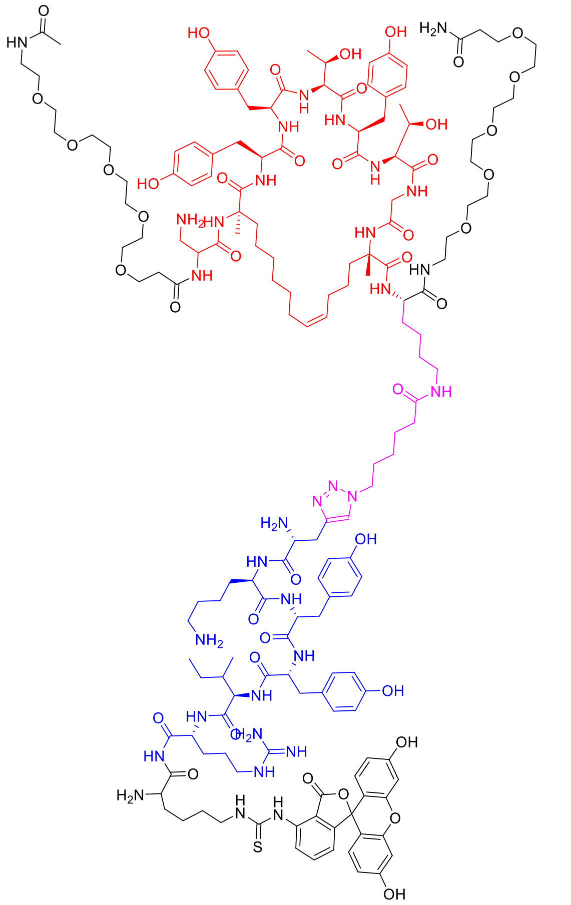

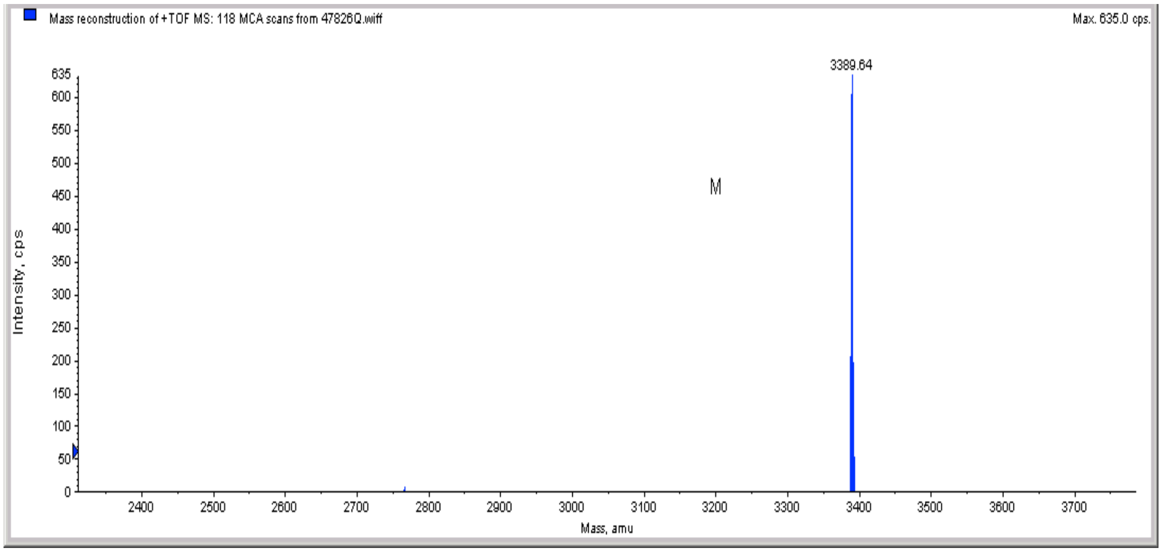

Mass spectrometric analysis of biotin tagged $B_{3,2,1}$

(figure 1C) Calculated mass:3054.65; Observed mass: 3055.83

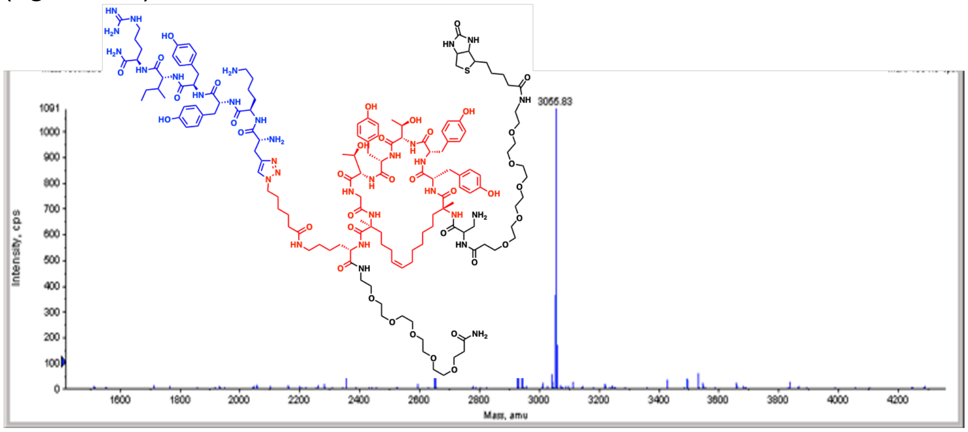

Mass spectrometric analysis of Cy(AYTYTG-rcm)

(figure S.14) Expected mass:1525.81, Observed mass:1525.77, m/z 763.89 (M+2H$^+$)/2; 509.60, (M+3H$^+$)/3

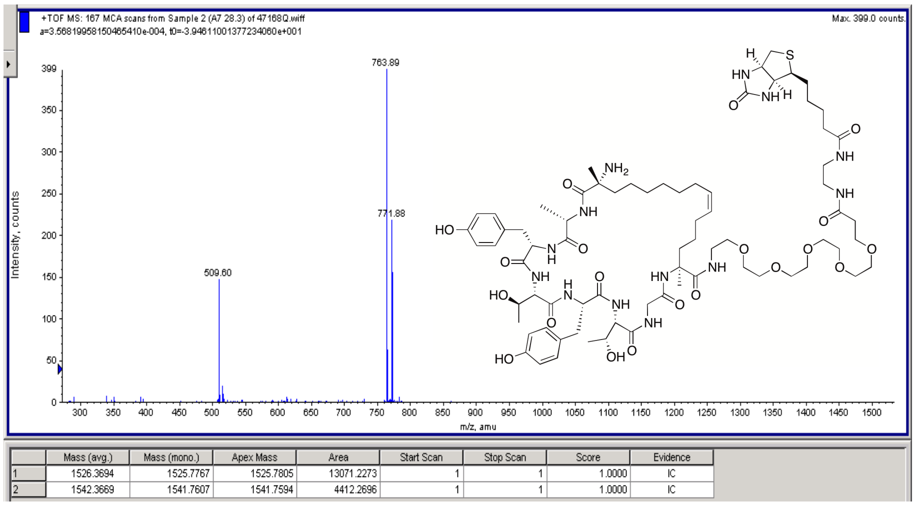

	Mass (avg.)	Mass (mono.)	Apex Mass	Area	Start Scan	Stop Scan	Score	Evidence
1	1526.3694	1525.7767	1525.7805	13071.2273	1	1	1.0000	IC
2	1542.3669	1541.7607	1541.7594	4412.2696	1	1	1.0000	IC

Mass spectrometric analysis of Cy(YATYTG-rcm)

figure S.14) Expected mass:1525.81, Observed mass:1525.79, m/z 763.90 (M+2H+)/2

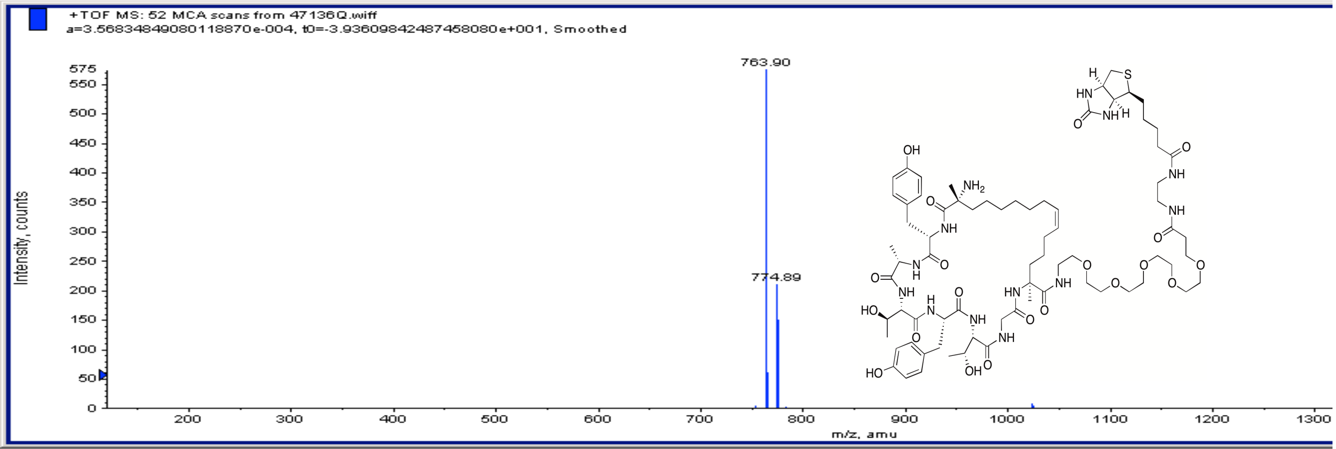

	Mass (avg.)	Mass (mono.)	Apex Mass	Area	Start Scan	Stop Scan	Score	Evidence
1	1526.2994	1525.7902	1525.7901	11513.0000	1	1	1.0000	I
2	1548.2202	1547.7682	1547.7677	3881.0000	1	1	1.0000	I

Mass spectrometric analysis of Cy(YYAYTG-rcm)
(figure S.14) Expected mass: 1587.83; Observed mass:1587.8, m/z (M+Na+)/1 1610.42;

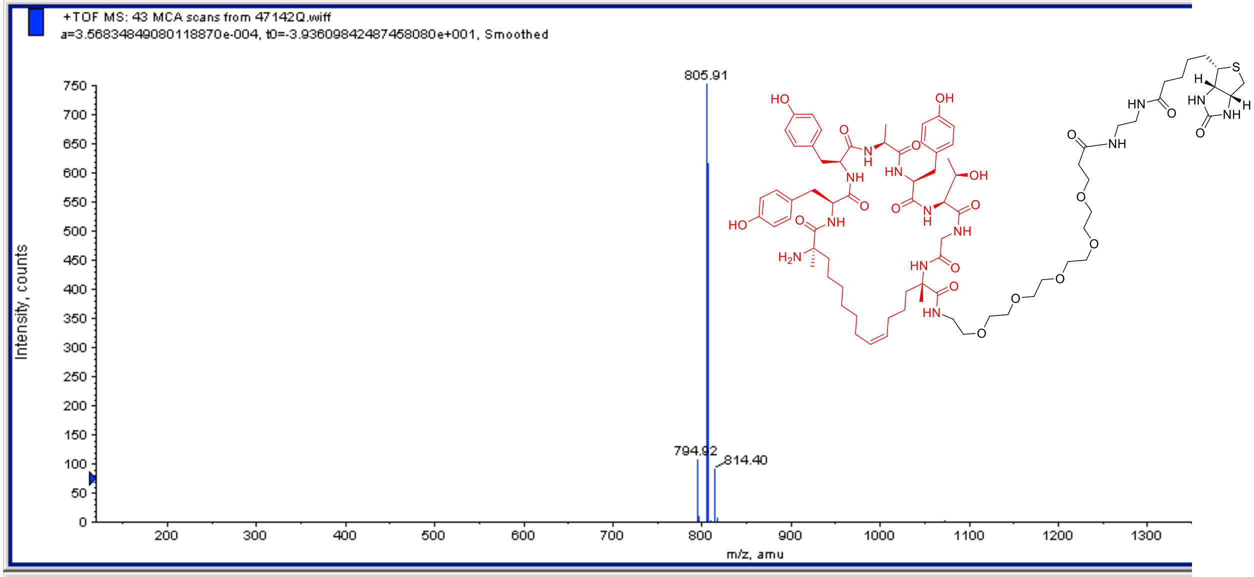

	Mass (avg.)	Mass (mono.)	Apex Mass	Area	Start Scan	Stop Scan	Score	Evidence
1	1610.4266	1609.8156	1609.8125	15700.0000	1	1	1.0000	I
2	1588.3010	1587.8282	1587.8288	1779.0000	1	1	1.0000	I
3	1626.2913	1625.7880	1626.7933	1689.0000	1	1	0.9999	I

Mass spectrometric analysis of Cy(YYTATG-rcm) (figure S.14)
Expected mass (M): 1525.81; Observed mass:(M) 1525.82, m/z 763.92 (M+2H+)/2

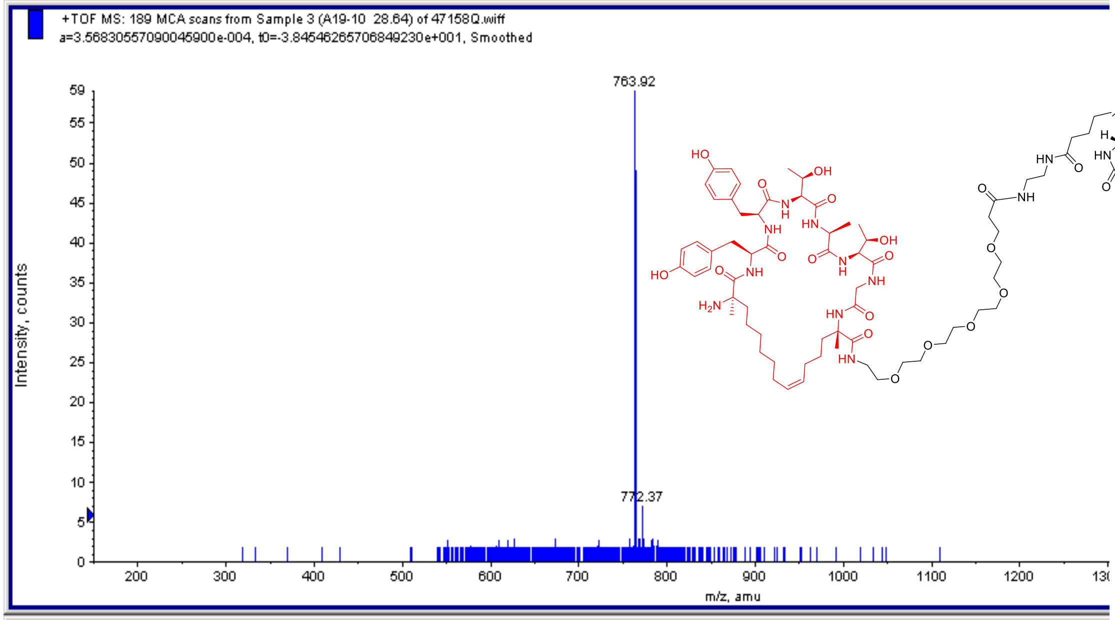

	Mass (avg.)	Mass (mono.)	Apex Mass	Area	Start Scan	Stop Scan	Score	Evidence
1	1526.3808	1525.8226	1525.8269	971.0000	1	1	0.9999	I

Mass spectrometric analysis of Cy(YYTYAG-rcm):

(figure S.14) Expected mass (M): 1587.83; Observed mass: 1587.84, m/z 794.92 (M+2H+)/2; 805.24 (M+H+Na+)/2

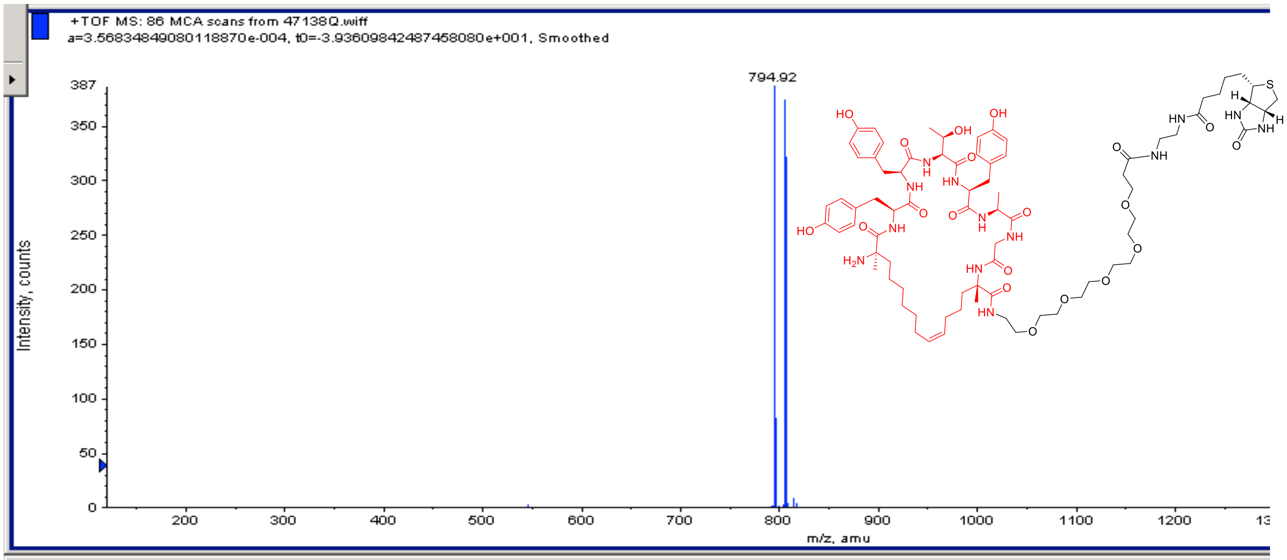

	Mass (avg.)	Mass (mono.)	Apex Mass	Area	Start Scan	Stop Scan	Score	Evidence
1	1588.4135	1587.8168	1587.8196	9300.0000	1	1	1.0000	I
2	1610.4974	1609.7966	1609.7993	9058.0000	1	1	1.0000	I

Mass Spectrometric analysis of cy(4fFYTYTG-rcm):

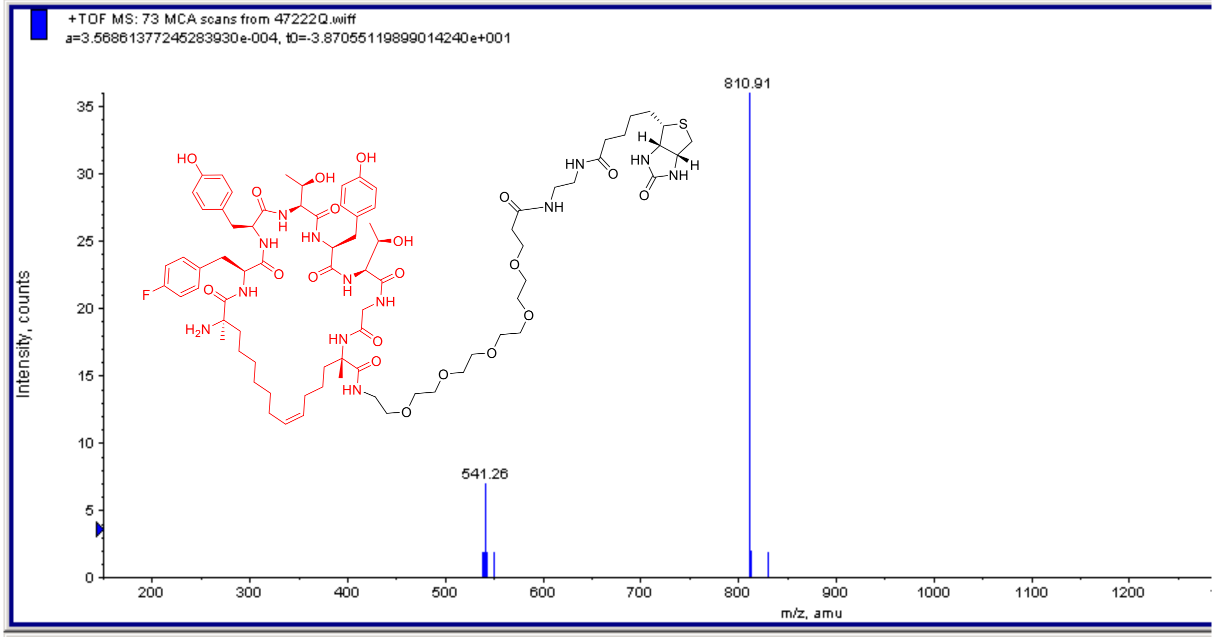

	Mass (avg.)	Mass (mono.)	Apex Mass	Area	Start Scan	Stop Scan	Score	Evidence
1	1620.5186	1619.7972	1620.7834	568.0000	1	1	1.0000	IC

Mass spectrometric analysis of cy(4fFYTYTG-rcm):

(figure S.15) Expected mass (M): 1619.83; Observed mass: 1619.82, m/z 810.91 (M+2H+)/2

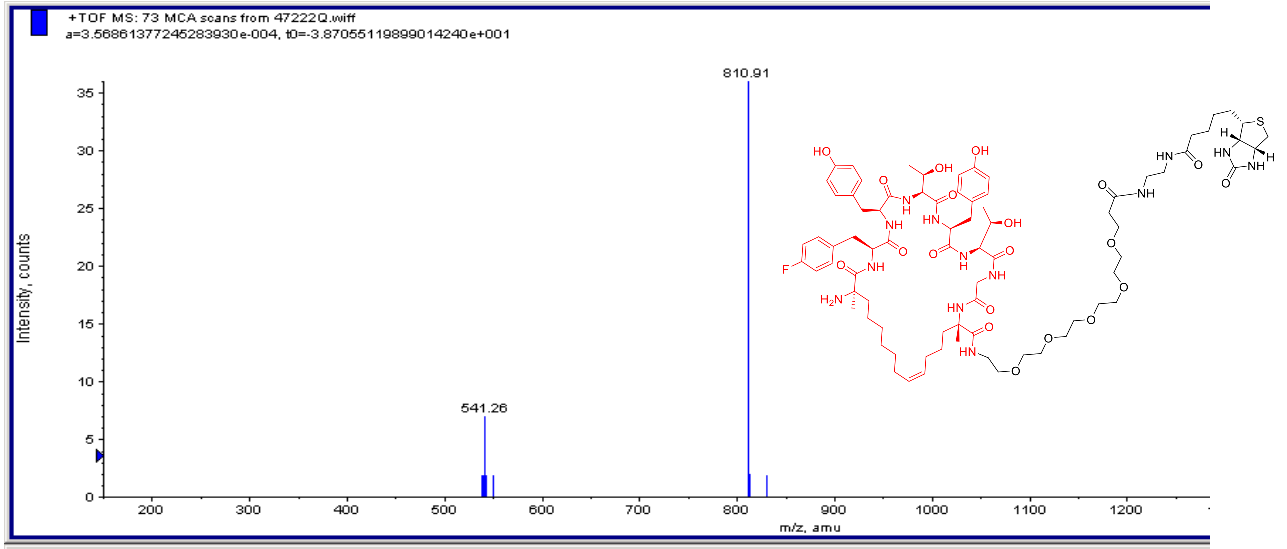

	Mass (avg.)	Mass (mono.)	Apex Mass	Area	Start Scan	Stop Scan	Score	Evidence
1	1620.5186	1619.7972	1620.7834	568.0000	1	1	1.0000	IC

Mass spectrometric analysis of Cy(Y4fFTYTG-rcm):

(figure S.15) Expected mass (M): 1619.83; Observed mass: 1619.80, 810.90 $(M+2H^{+})/2$

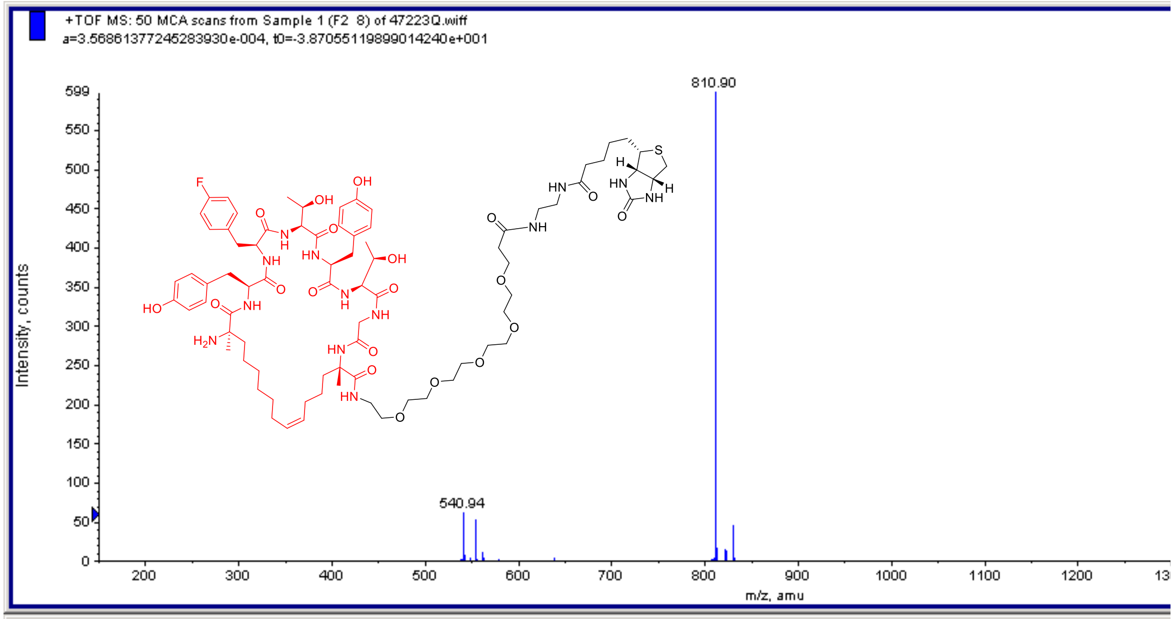

	Mass (avg.)	Mass (mono.)	Apex Mass	Area	Start Scan	Stop Scan	Score	Evidence
1	1620.5438	1619.7991	1620.8057	12069.0000	1	1	1.0000	IC

Mass Spectrometric analysis of Cy(YYT4fFTG-rcm): (figure S.15)

Expected mass (M): 1619.83; Observed mass: 1619.89, m/z 810.95 $(M+2H^+)/2$

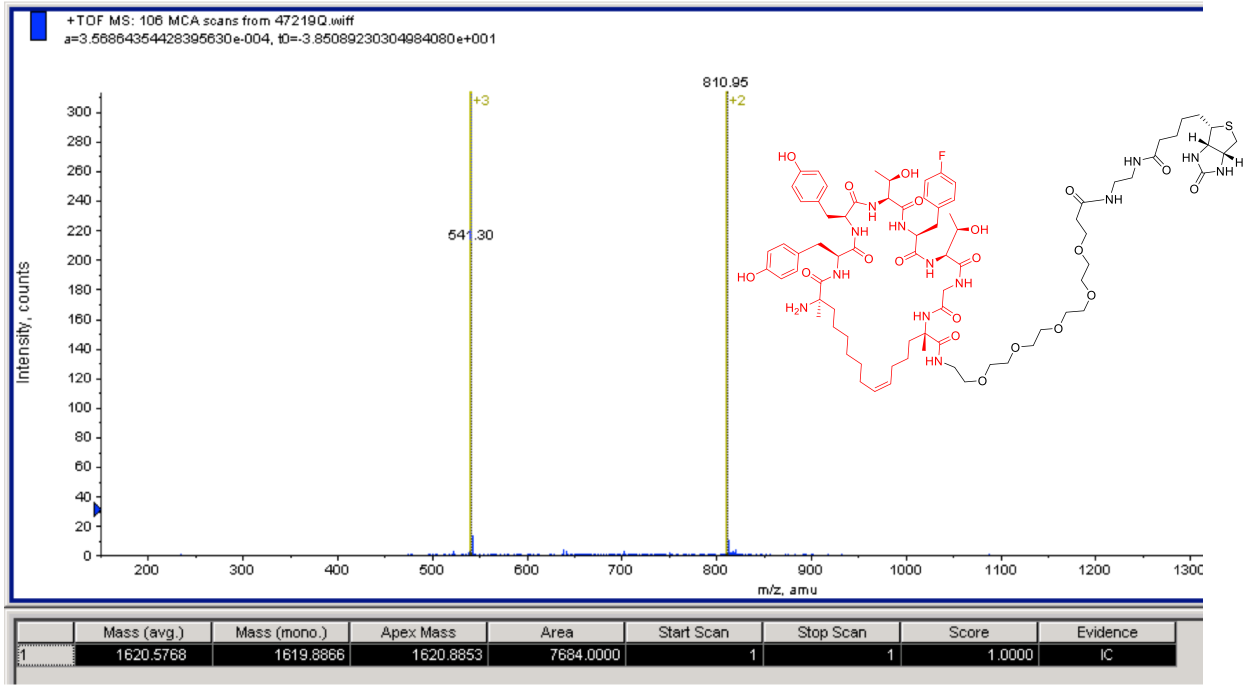

Mass Spectrometric analysis of Cy(4fF4fFTYTG-rcm): (figure S.15)
Expected mass (M): 1621.83; Observed mass:1621.84, m/z $(M+ H^++Na^+)/2$

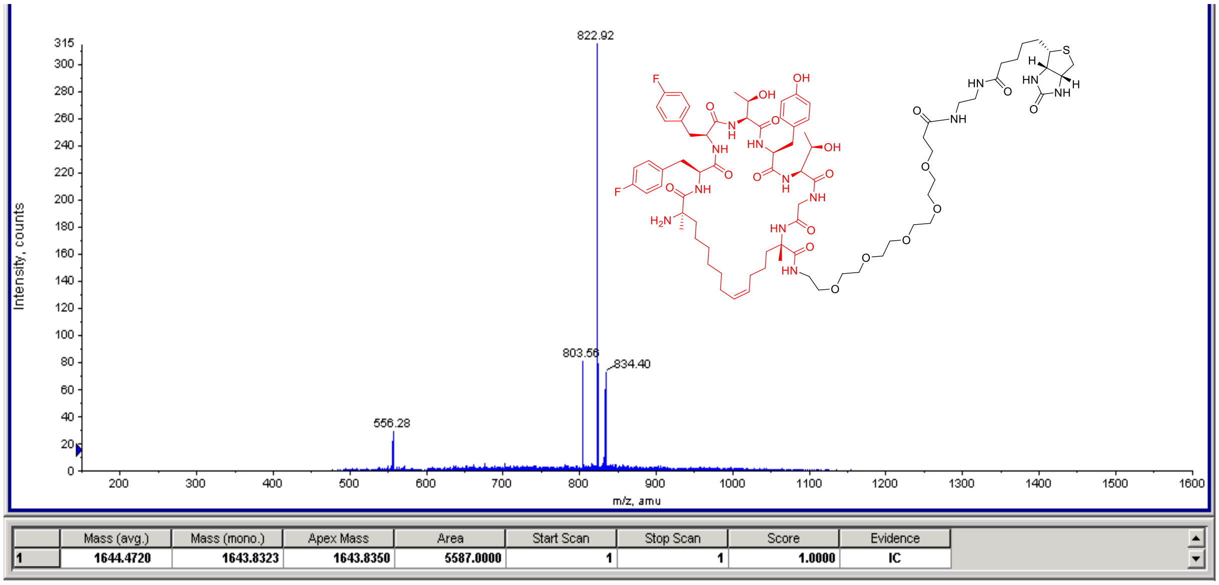

Mass Spectrometric analysis of Cy(YYTY’T’G-rcm):

(figure S.15) Y’ -N-Me Tyr; T’: N-Me Thr; Expected mass (M): 1645.87; Observed mass: 1645.87

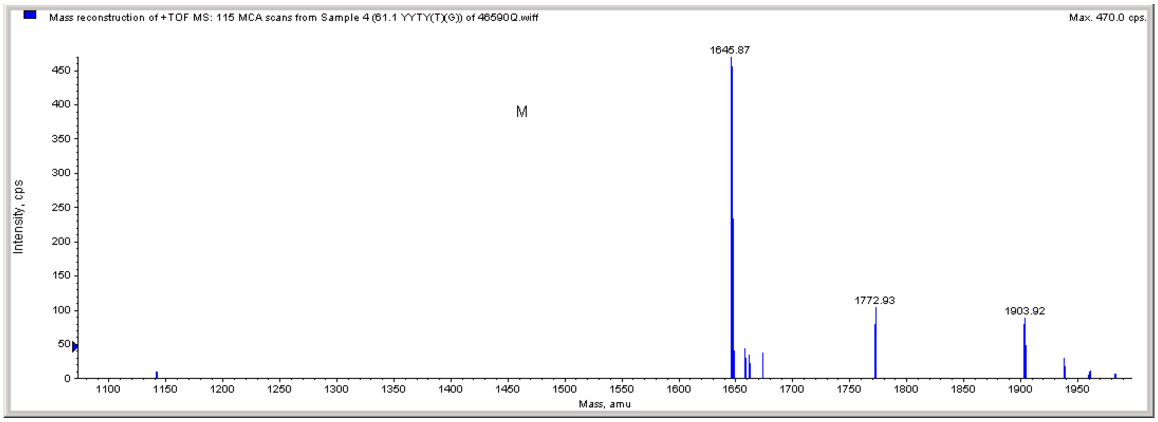

Mass Spectrometric analysis of Cy(Y’YTY’T’G-rcm):

(figure S.15) Y’: N-Me Tyr; T’: N-Me Thr; Expected mass: 1659.88; Observed mass:1659.9, m/z 830.95 $(M+2H^+)/2$

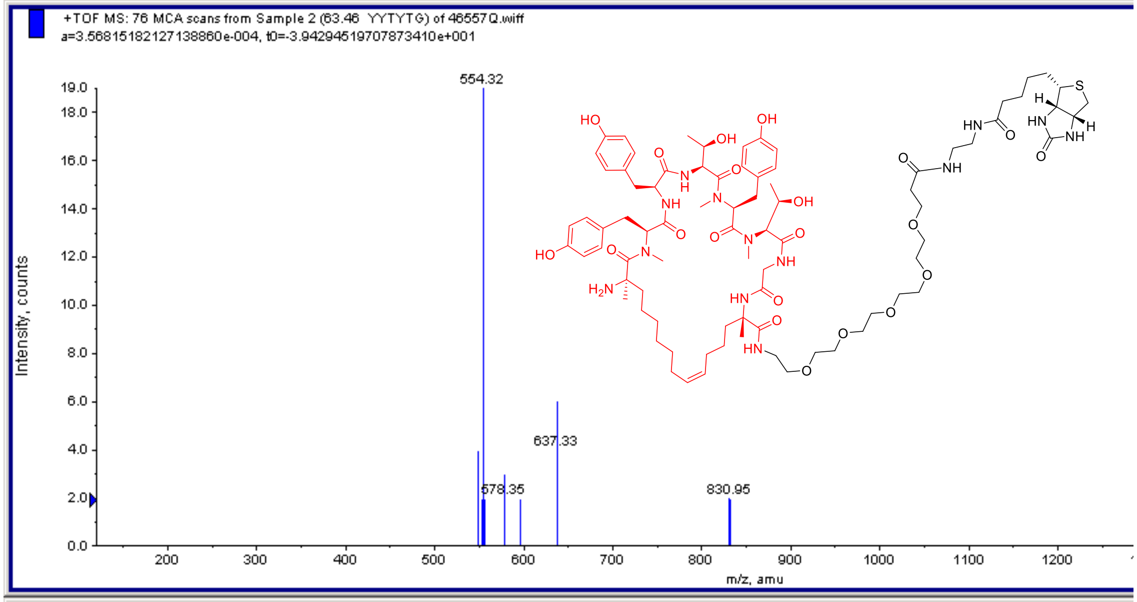

	Mass (avg.)	Mass (mono.)	Apex Mass	Area	Start Scan	Stop Scan	Score	Evidence
1	1660.4927	1659.9358	1659.9386	181.0000	1	1	1.0000	IC

Mass Spectrometric analysis of $B_{3,2,5}$:

(Figure S.19) Calculated mass: 3098.69 Observed mass: 3098.57

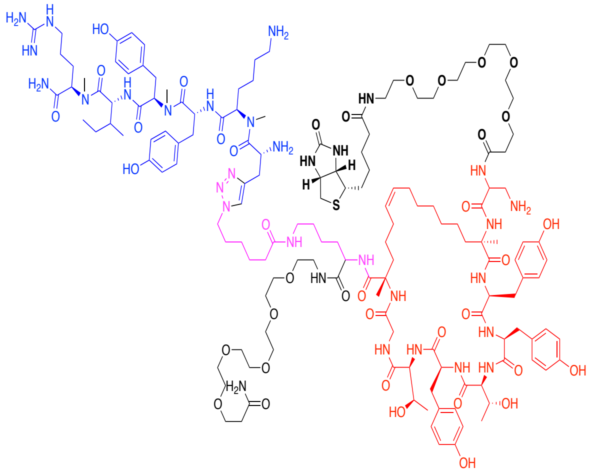

Molecular Weight: 3098.69

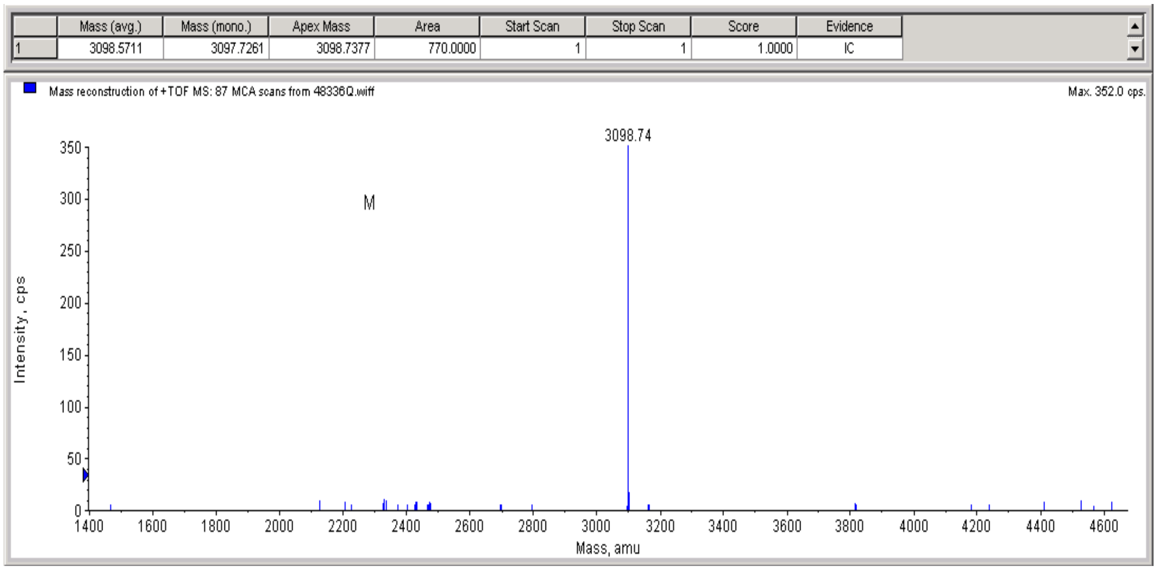

Mass spectrometric analysis of B4,2,5:

(Figure S. 20) Calculated mass: 3100.69 Observed mass: 3100.80

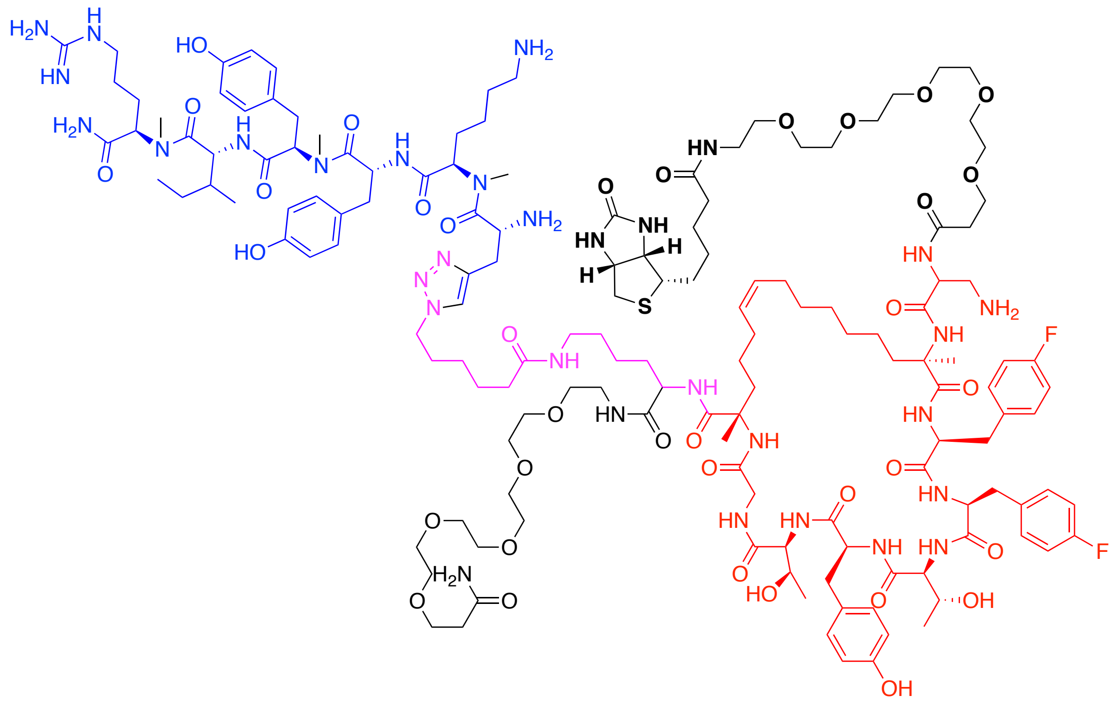

Molecular Weight: 3102.68

	Mass (avg.)	Mass (mono.)	Apex Mass	Area	Start Scan	Stop Scan	Score	Evidence
1	3100.6811	3098.8056	3100.8022	13013.2807	1	1	1.0000	IC

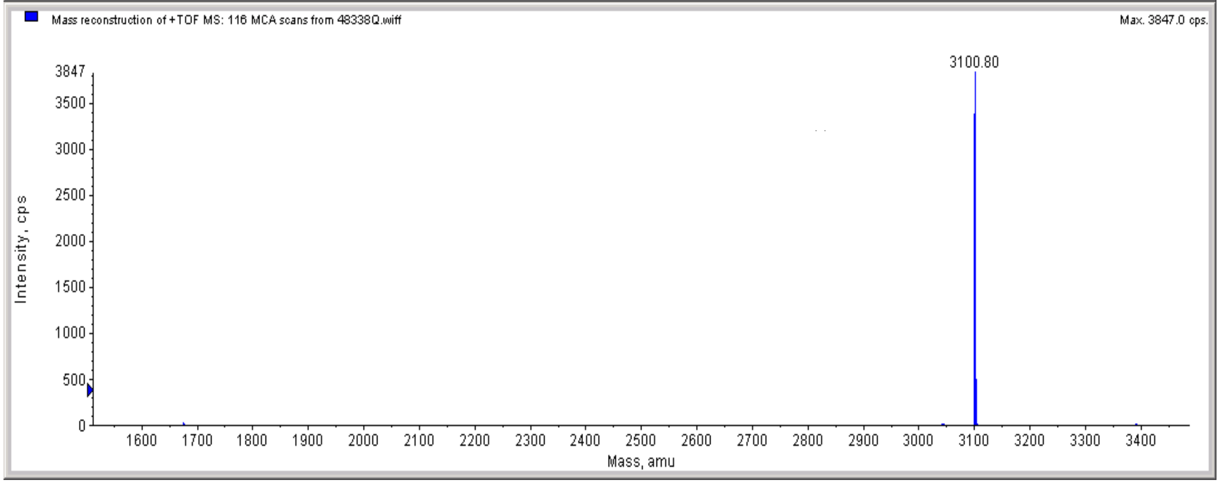

Mass Spectrometric analysis of $B_{6,3,5}$

(Figure 4A) Calculated mass:2334.29; Observed mass:2334.28

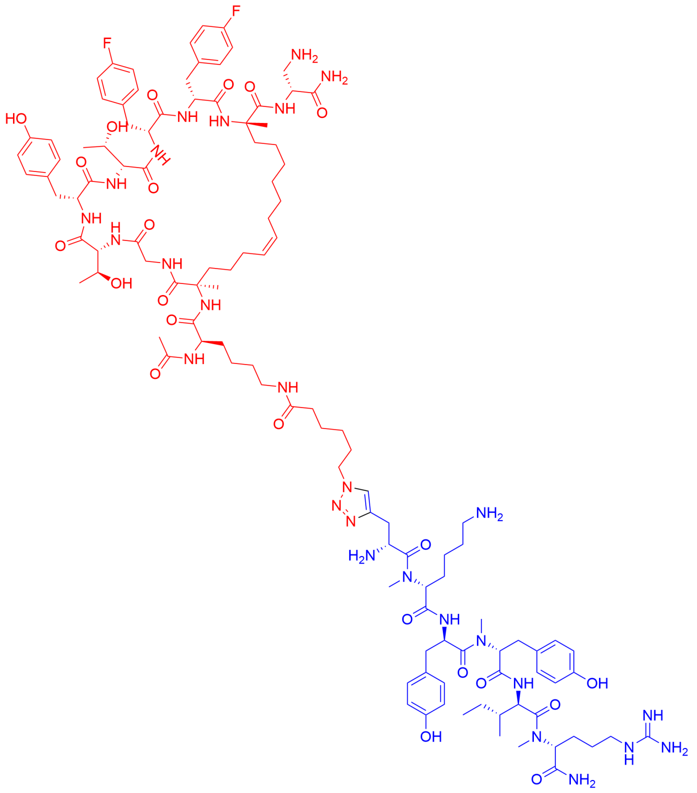

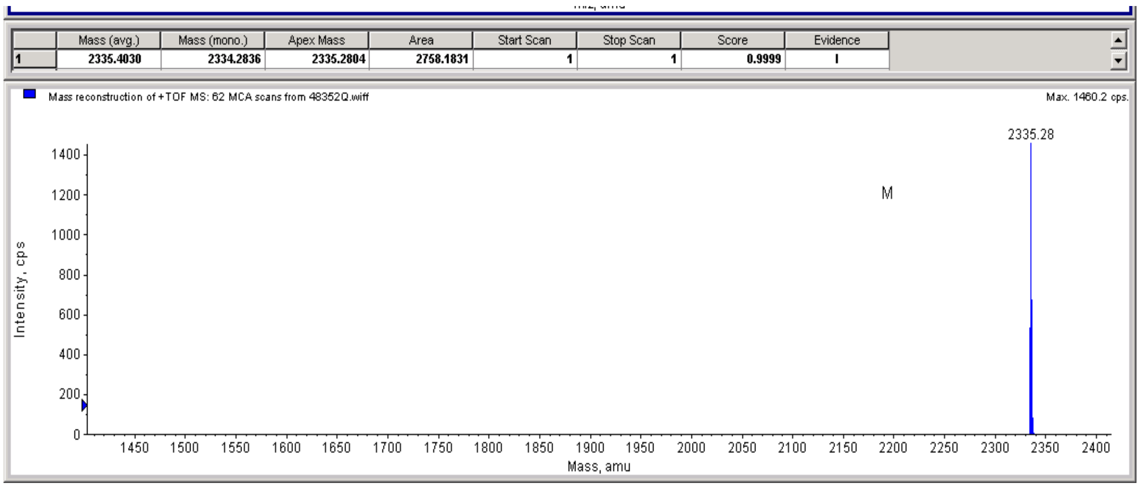

Mass Spectrometric analysis of $B_{5,3,4}$

(figure 4A) Calculated mass: 2320.27; Observed mass: 2321.31, m/z 774.77 $(M+3H^+)/3$

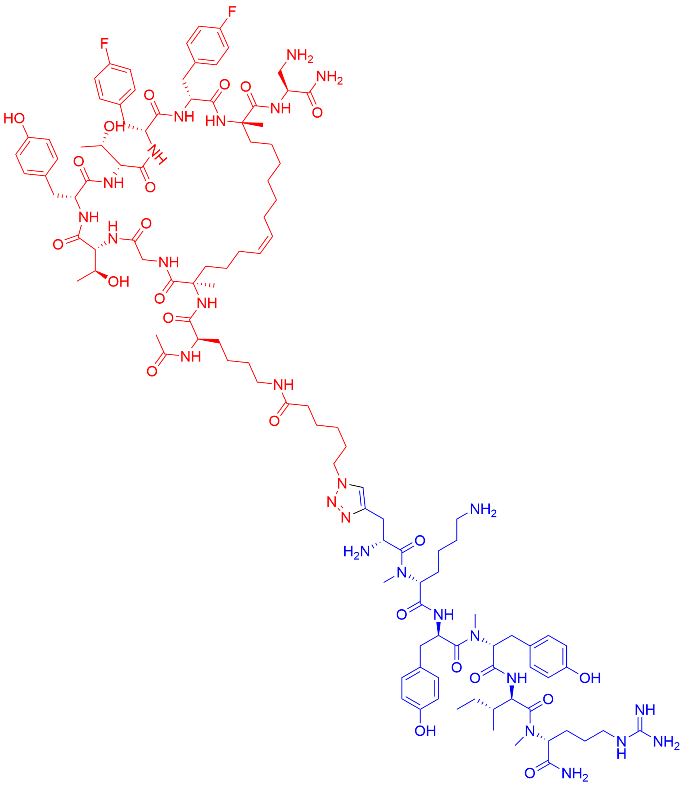

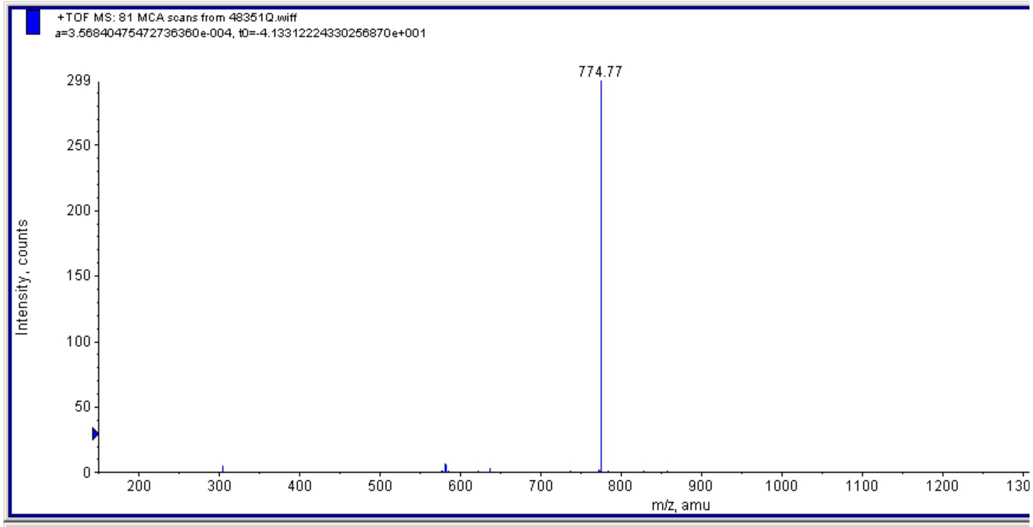

	Mass (avg.)	Mass (mono.)	Apex Mass	Area	Start Scan	Stop Scan	Score	Evidence
1	2321.3681	2320.2800	2321.2775	2750.0000	1	1	1.0000	IC

Mass Spectrometric analysis of $B_{5,3,5}$

(figure 4A) Calculated mass: 2334.29; Observed mass: 2334.25

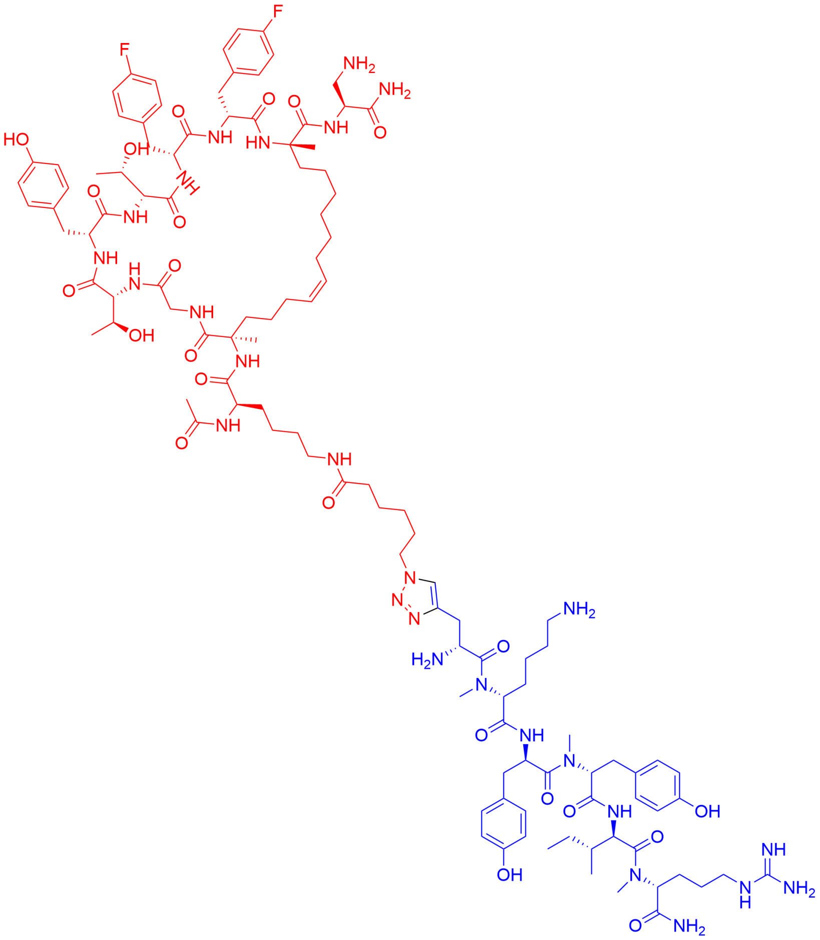

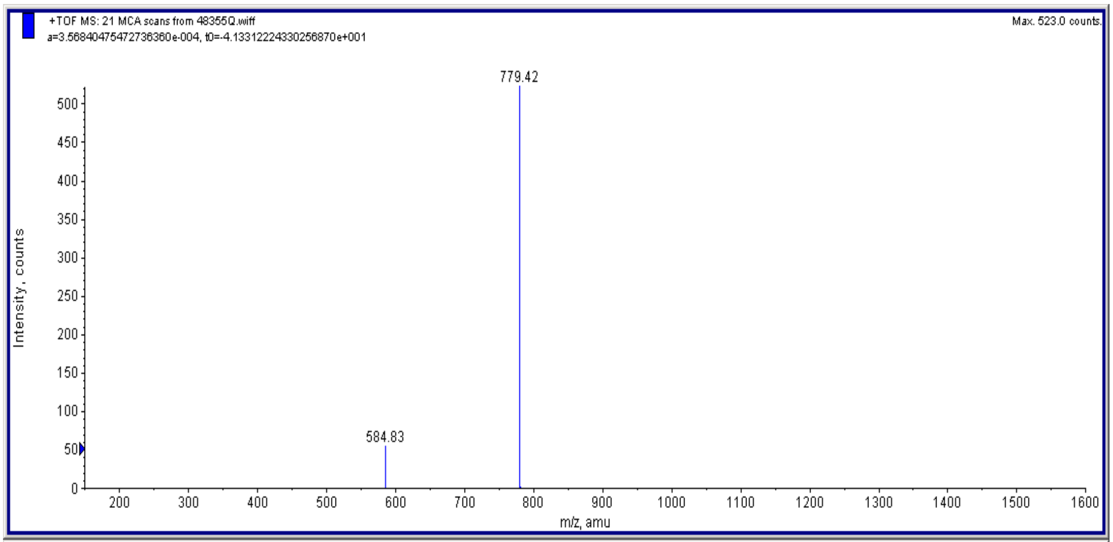

	Mass (avg.)	Mass (mono.)	Apex Mass	Area	Start Scan	Stop Scan	Score	Evidence
1	2335.3112	2334.2514	2335.2517	5921.0000	1	1	1.0000	I

Mass Spectrometric analysis of fluorescein conjugated $B_{5,3,5}$

(Figure 4B) Calculated mass: 2752.35; Observed mass: 2753.28, m/z 918.76 $(M+3H^+)/3$, 689.06 $(M+4H^+)/4$

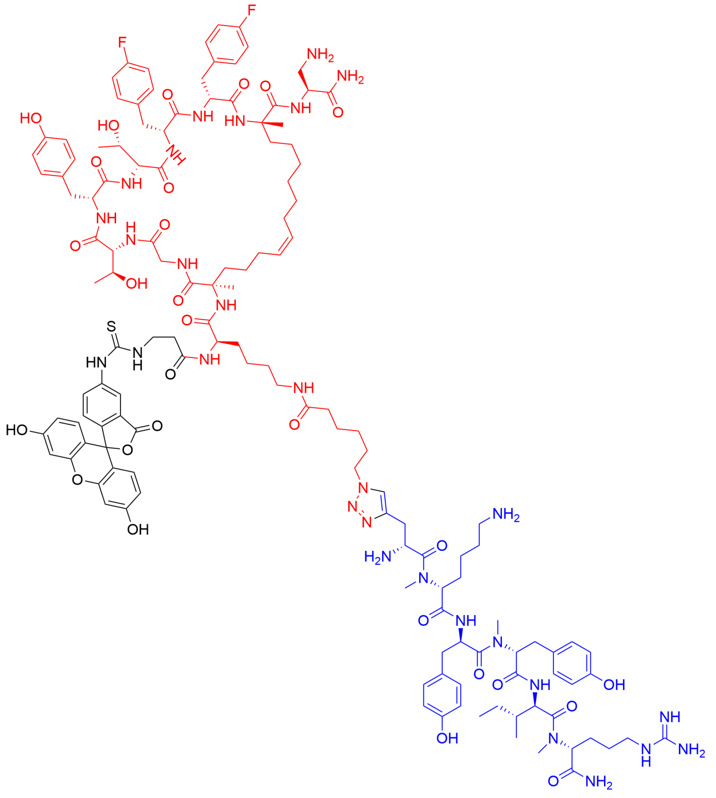

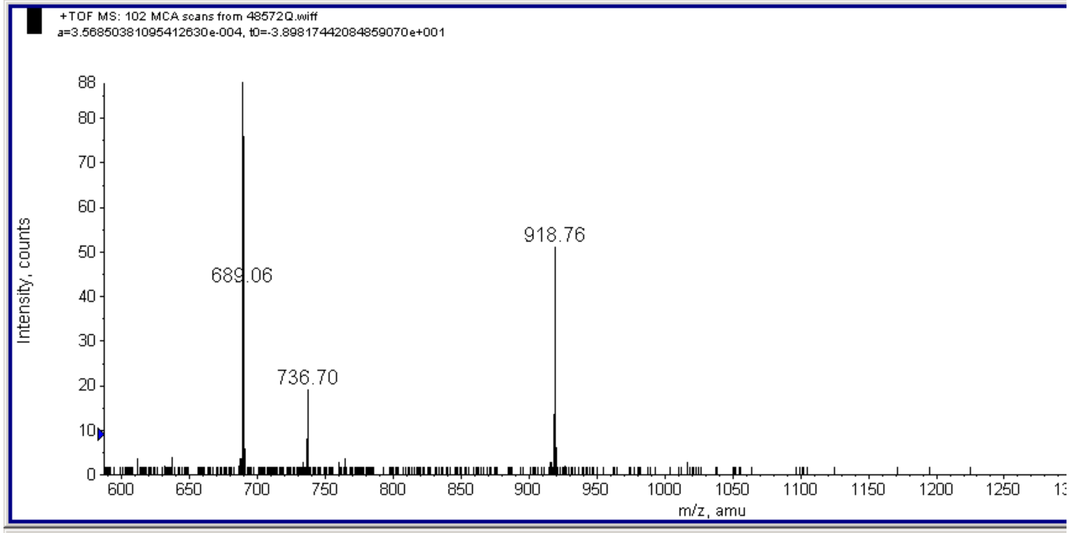

	Mass (avg.)	Mass (mono.)	Apex Mass	Area	Start Scan	Stop Scan	Score	Evidence
1	2753.6316	2752.2439	2753.2328	1331.0000	1	1	1.0000	IC

Mass Spectrometric analysis of fluorescein conjugated $B_{7,3,5}$

(Figure 4B) Calculated mass: 2752.35; Observed mass: 2754.91, m/z 2755.91 ($M+H^+$)

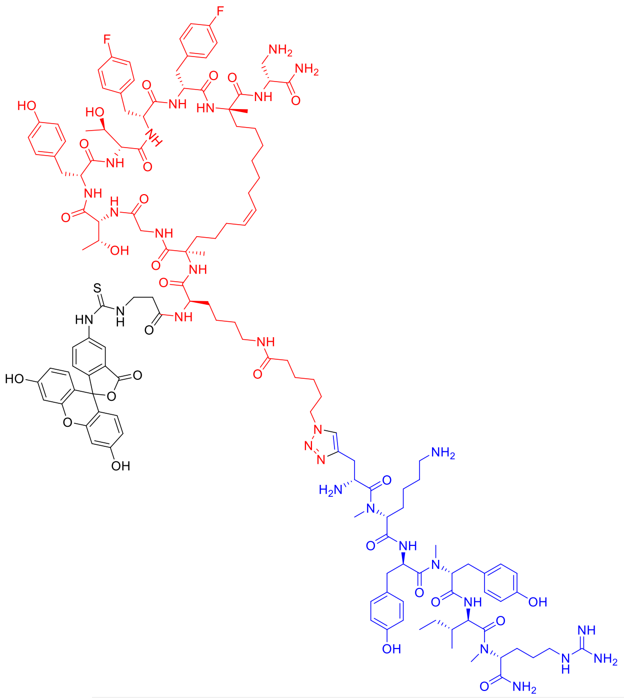

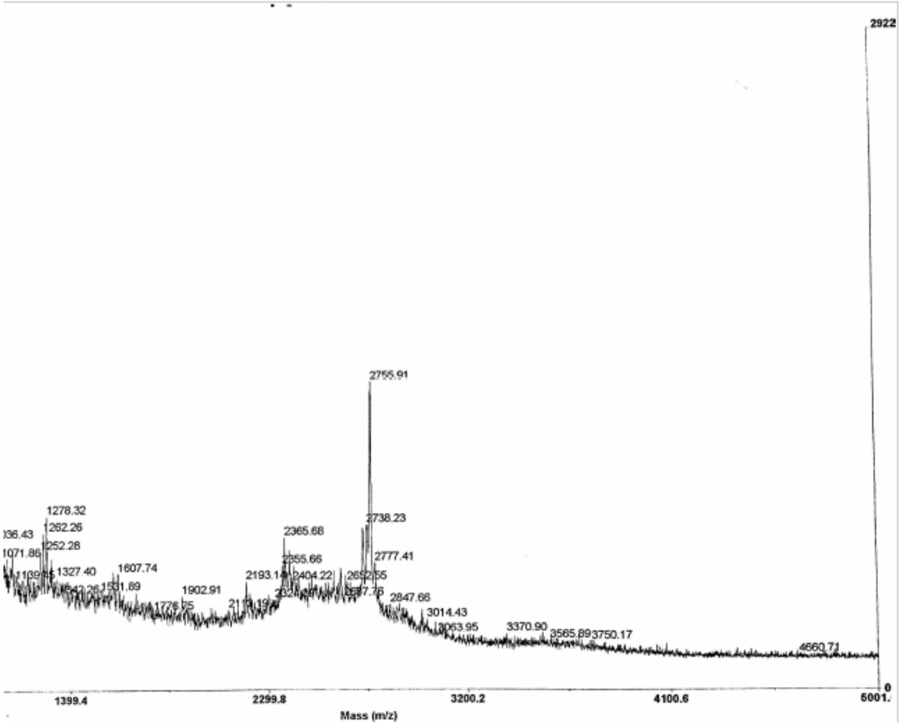

Mass Spectrometry of Biotin-tagged $B_{5,3,5}$

Expected mass: 2809.52; Observed mass:2811.06

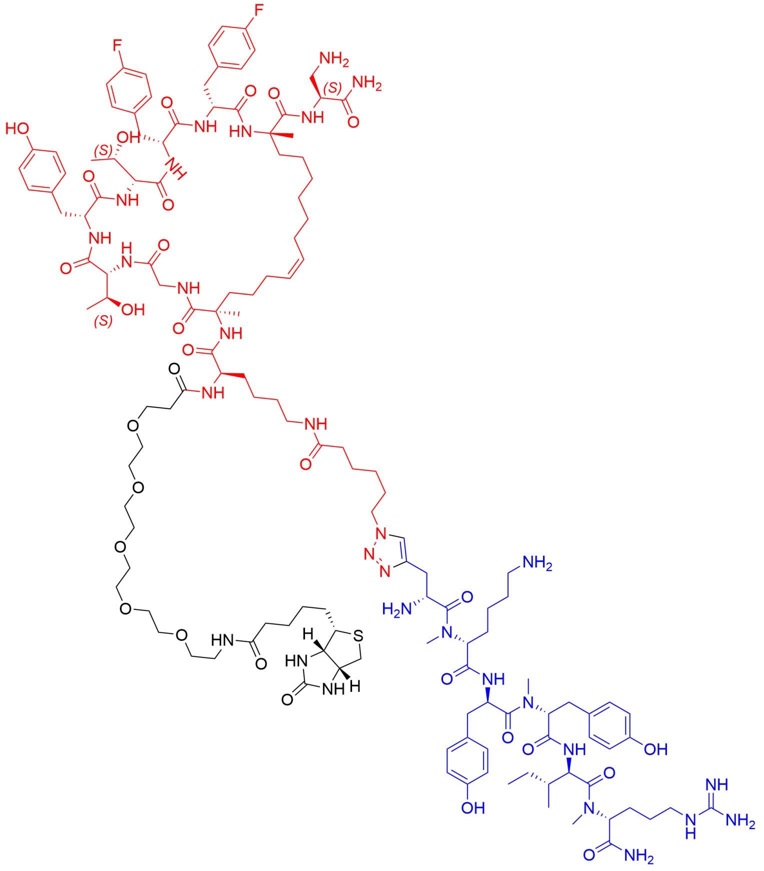

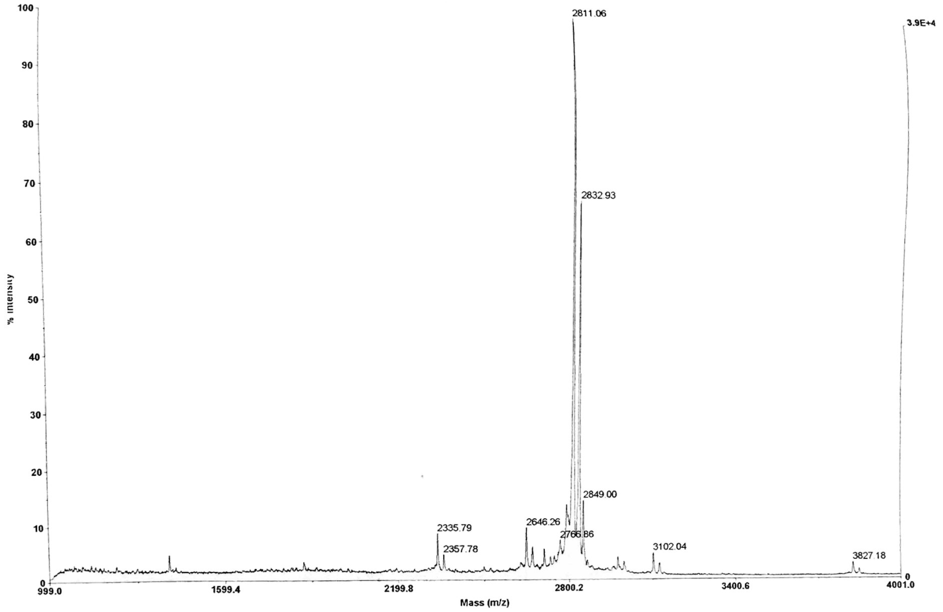

Mass spectrometric analysis of Biotin tagged $B_{6,3,5}$

(Figure 4D) Expected mass: 2809.52; Observed mass:2811.06

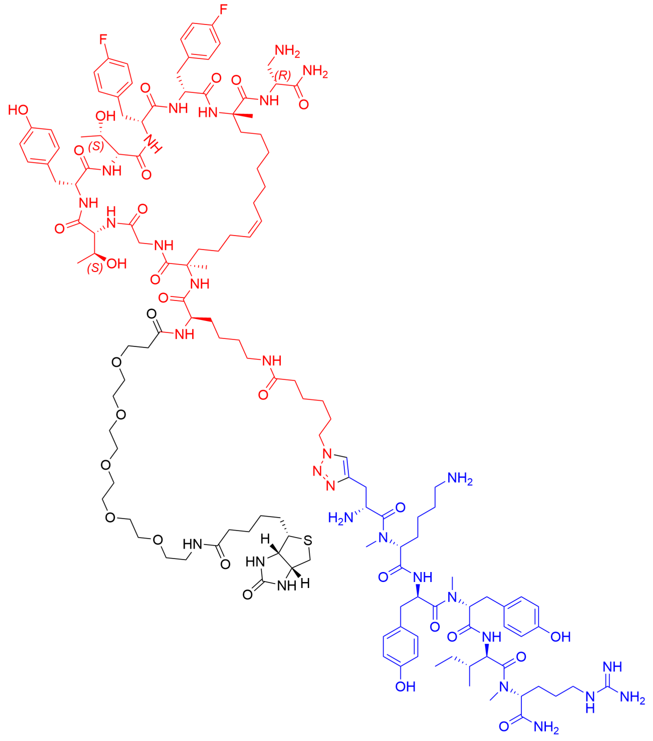

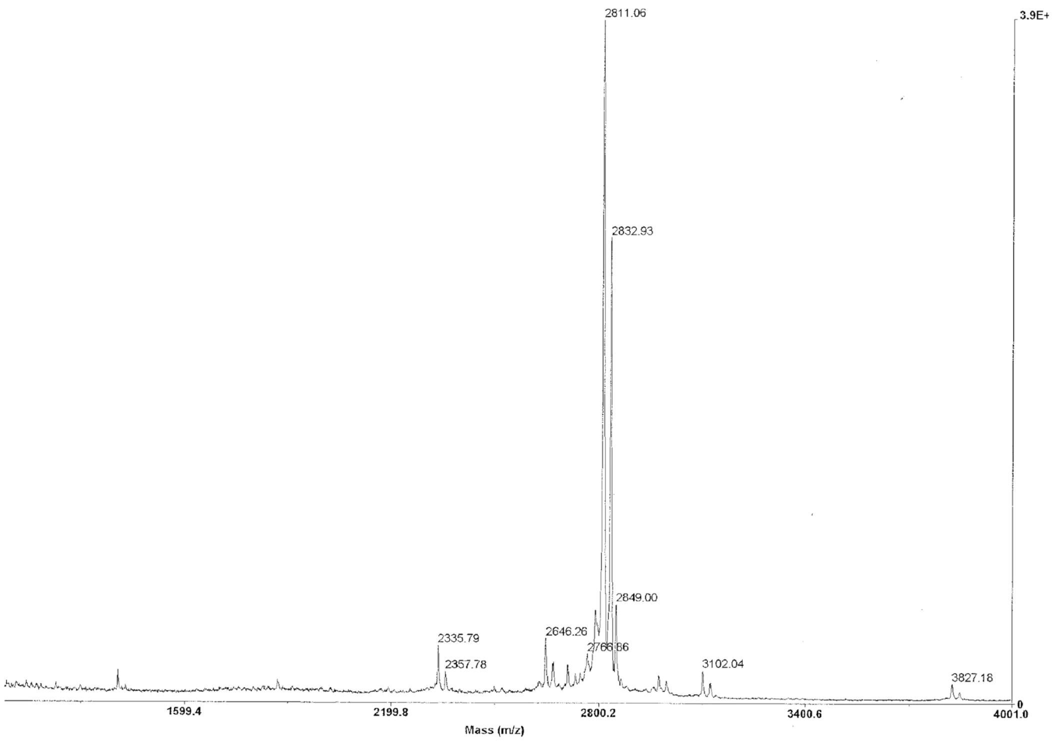

Mass spectrometric analysis of Biotin tagged $B_{6,3,4}$

(Figure 4D) Expected mass:2795.50; Observed mass:2796.53

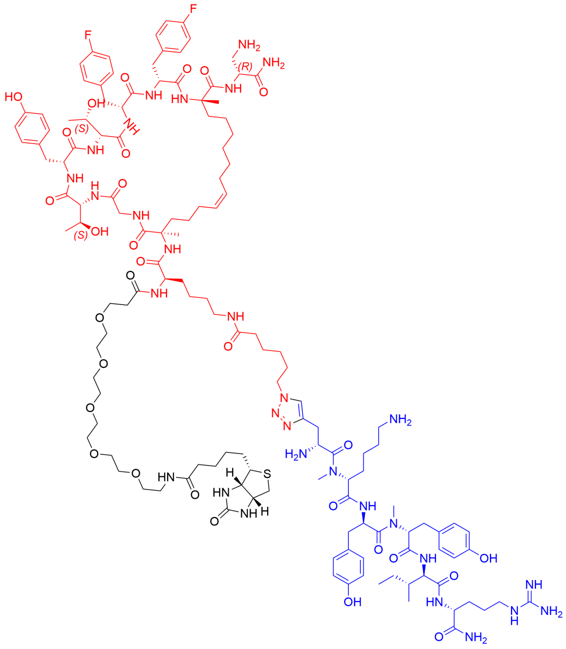

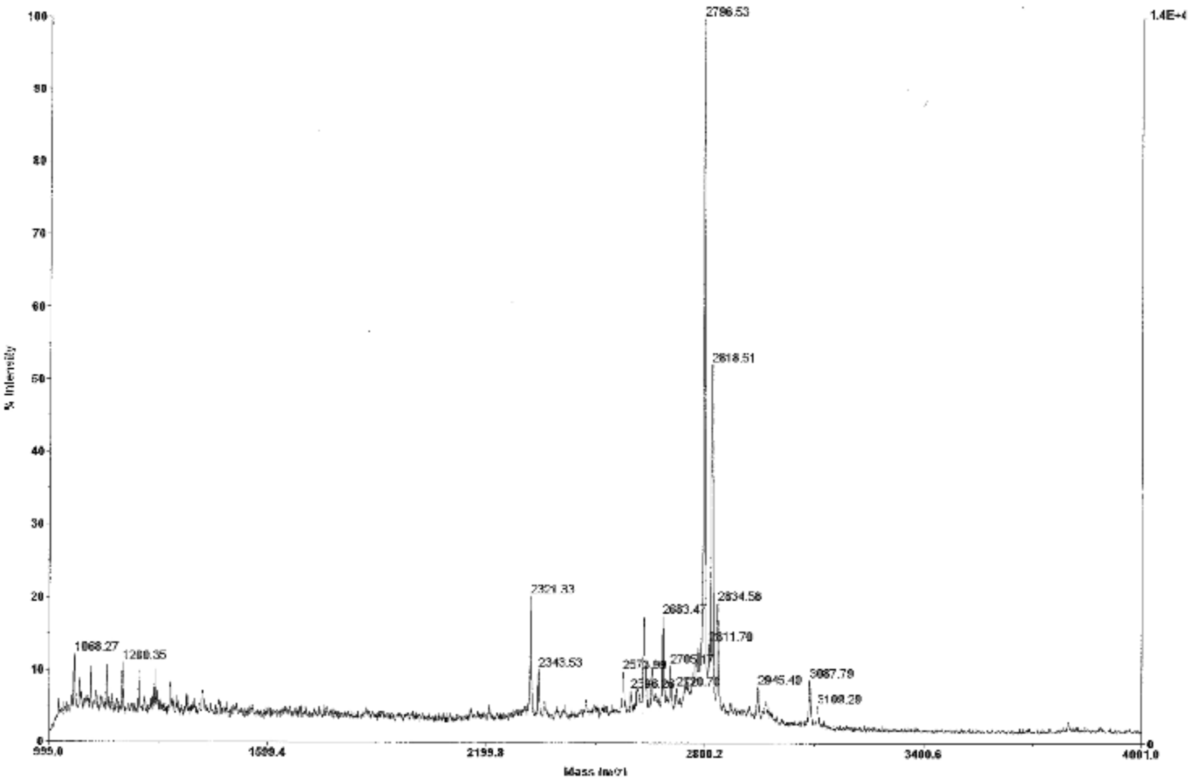

Mass spectrometric analysis of Biotin tagged $B_{7,3,5}$

(Figure 4D) Expected mass: 2809.52; Observed mass:2808.15

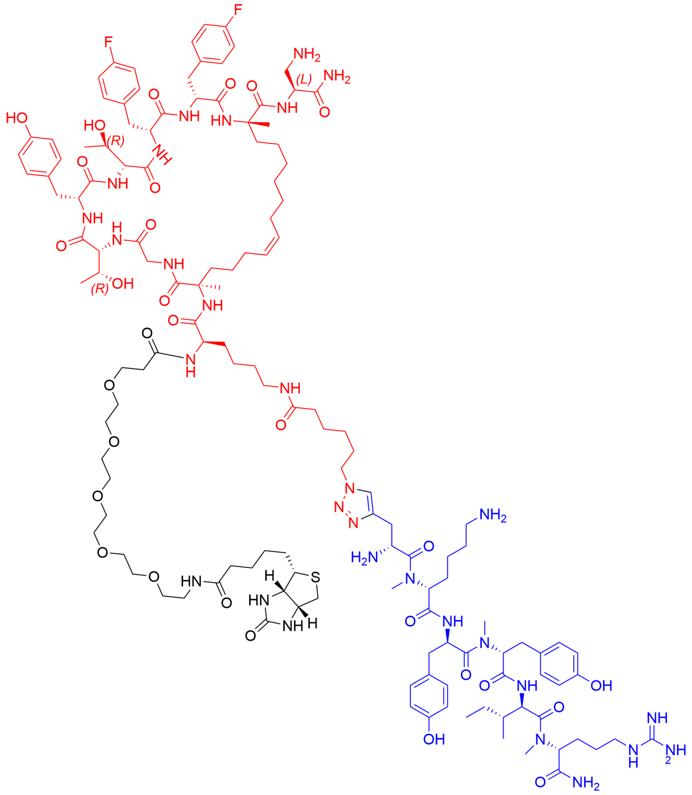

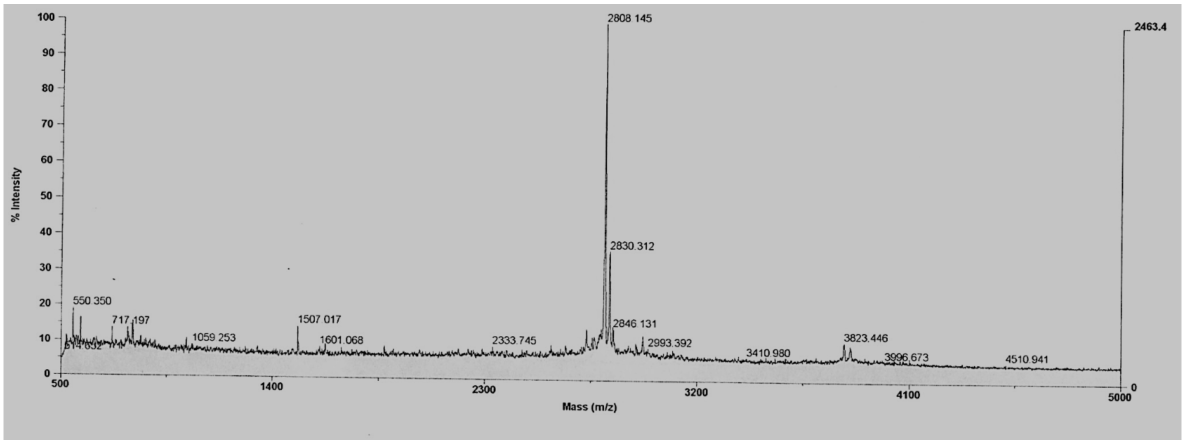

Supplement: Supplementary file 1 — Supplementary Information [file 42004_2023_890_MOESM1_ESM.pdf]
