# Supplementary material for: Stereochemical engineering yields a multifunctional peptide macrocycle inhibitor of Akt2 by fine-tuning macrocycle-cell membrane interactions
Source: Commun Chem. 2023 May 18;6:95. doi: 10.1038/s42004-023-00890-w (PMC10195864; doi:10.1038/s42004-023-00890-w)
Supplement: Supplementary file 2 — Reporting Summary [file 42004_2023_890_MOESM2_ESM.pdf]

nature portfolio

Corresponding author(s): James R. Heath

Last updated by author(s): Apr 20, 2023

Reporting Summary

Nature Portfolio wishes to improve the reproducibility of the work that we publish. This form provides structure for consistency and transparency in reporting. For further information on Nature Portfolio policies, see our Editorial Policies and the Editorial Policy Checklist.

Statistics

For all statistical analyses, confirm that the following items are present in the figure legend, table legend, main text, or Methods section.

n/a	Confirmed	
☐	☒	The exact sample size (*n*) for each experimental group/condition, given as a discrete number and unit of measurement
☐	☒	A statement on whether measurements were taken from distinct samples or whether the same sample was measured repeatedly
☒	☐	The statistical test(s) used AND whether they are one- or two-sided<br>*Only common tests should be described solely by name; describe more complex techniques in the Methods section.*
☒	☐	A description of all covariates tested
☒	☐	A description of any assumptions or corrections, such as tests of normality and adjustment for multiple comparisons
☐	☒	A full description of the statistical parameters including central tendency (e.g. means) or other basic estimates (e.g. regression coefficient) AND variation (e.g. standard deviation) or associated estimates of uncertainty (e.g. confidence intervals)
☒	☐	For null hypothesis testing, the test statistic (e.g. *F*, *t*, *r*) with confidence intervals, effect sizes, degrees of freedom and *P* value noted<br>*Give P values as exact values whenever suitable.*
☒	☐	For Bayesian analysis, information on the choice of priors and Markov chain Monte Carlo settings
☒	☐	For hierarchical and complex designs, identification of the appropriate level for tests and full reporting of outcomes
☒	☐	Estimates of effect sizes (e.g. Cohen's *d*, Pearson's *r*), indicating how they were calculated

Our web collection on statistics for biologists contains articles on many of the points above.

Software and code

Policy information about availability of computer code

Data collection	no code or specialized software was used for data collection. Standard widely avaialble software such as was used for flow cytometry analysis.
Data analysis	no code or specialized software was used for data analysis. Standard widely avaialble software such as FlowJo software 10.4.1 (Treestar) was used for flow cytometry analysis. Prism 6.0h (GraphPad) was used to generate graphs and perform statistical analysis

For manuscripts utilizing custom algorithms or software that are central to the research but not yet described in published literature, software must be made available to editors and reviewers. We strongly encourage code deposition in a community repository (e.g. GitHub). See the Nature Portfolio guidelines for submitting code & software for further information.

Data

Policy information about availability of data

All manuscripts must include a data availability statement. This statement should provide the following information, where applicable:

- Accession codes, unique identifiers, or web links for publicly available datasets
- A description of any restrictions on data availability
- For clinical datasets or third party data, please ensure that the statement adheres to our policy

Provide your data availability statement here.

Human research participants

Policy information about studies involving human research participants and Sex and Gender in Research.

Reporting on sex and gender	Not applicable
Population characteristics	Not applicable
Recruitment	Not applicable
Ethics oversight	Not applicable

Note that full information on the approval of the study protocol must also be provided in the manuscript.

Field-specific reporting

Please select the one below that is the best fit for your research. If you are not sure, read the appropriate sections before making your selection.

☒ Life sciences ☐ Behavioural & social sciences ☐ Ecological, evolutionary & environmental sciences

For a reference copy of the document with all sections, see nature.com/documents/nr-reporting-summary-flat.pdf

Life sciences study design

All studies must disclose on these points even when the disclosure is negative.

Sample size	Samples were prepared in triplicate and measured during an experiment
Data exclusions	No data was excluded from the analysis
Replication	Important experiments included in the main figure were done using samples in triplicate.
Randomization	Not applicable
Blinding	Not applicable

Reporting for specific materials, systems and methods

We require information from authors about some types of materials, experimental systems and methods used in many studies. Here, indicate whether each material, system or method listed is relevant to your study. If you are not sure if a list item applies to your research, read the appropriate section before selecting a response.

Materials & experimental systems

n/a	Involved in the study	
☐	☒	Antibodies
☐	☒	Eukaryotic cell lines
☒	☐	Palaeontology and archaeology
☒	☐	Animals and other organisms
☒	☐	Clinical data
☒	☐	Dual use research of concern

Methods

n/a	Involved in the study	
☒	☐	ChIP-seq
☐	☒	Flow cytometry
☒	☐	MRI-based neuroimaging

Antibodies

Antibodies used	Mouse anti-biotin antibody-Alkaline Phosphatase conjugate, Sigma Aldrich, catalog number A6561; Anti His6 mouse antibody, Abcam, catalog number ab18184 ; goat anti mouse IgG - Alkaline Phosphatase, Abcam, catalog number ab97020; Akt (pan) rabbit antibody (11E7), Cell Signaling Technology, catalog number 4685; Biotinylated mouse PhosphoS473 (pS473) Akt antibody, Cell Signaling Technology, catalog number; PhosphoAkt (S473) antibody, Cell Signaling Technology, catalog number 4060S; Anti-rabbit antibody (HRP conjugate), Cell Signaling Technology, catalog number 7074S; anti-biotin antibody (HRP conjugate), Cell Signaling Technology, catalog number 7075S, beta-actin mouse antibody, Cell Signaling Technology,3700S; pGSK3 antibody, Cell Signaling Technology catalog number 9327L; Alexa 790 conjugated goat anti-rabbit antibody, Abcam, ab186697; Alexa 700 conjugated goat anti-mouse antibody, Invitrogen, A21036

Validation	Primary antibodies with validation statements on manufacturer website were used as per manufacturer's specifications.

Eukaryotic cell lines

Policy information about cell lines and Sex and Gender in Research

Cell line source(s)	NIH OVCAR3 cells used in the experiments were bought from ATCC
Authentication	The cell lines used were not authenticated
Mycoplasma contamination	cell lines were not tested for mycoplasma contamination
Commonly misidentified lines (See ICLAC register)	No commonly misidentified cell lines were used

Flow Cytometry

Plots

Confirm that:

☒ The axis labels state the marker and fluorochrome used (e.g. CD4-FITC).

☒ The axis scales are clearly visible. Include numbers along axes only for bottom left plot of group (a 'group' is an analysis of identical markers).

☒ All plots are contour plots with outliers or pseudocolor plots.

☒ A numerical value for number of cells or percentage (with statistics) is provided.

Methodology

Sample preparation	NIH OVCAR3 cells, passage 3. Details are provided in the Methods section under the title Flow Cytometry
Instrument	Aria II (BD Biosciences)
Software	FlowJo LLC was used to analyze the data.
Cell population abundance	*Describe the abundance of the relevant cell populations within post-sort fractions, providing details on the purity of the samples and how it was determined.*
Gating strategy	Gating strategy provided in supplementary figure and described in the methods section under the title Flow Cytometry

☒ Tick this box to confirm that a figure exemplifying the gating strategy is provided in the Supplementary Information.
